# Heterogeneity and dynamics of DENV-specific CD8 + T cells in dengue infection

**DOI:** 10.1038/s41467-026-73491-5

**Published:** 2026-06-03

**Authors:** Sirawit Srikor, Waradon Sungnak, Chawinya Trakoolsoontorn, Tiraput Poonpanichakul, Natnicha Jiravejchakul, Damita Jevapatarakul, Narita Thungsatianpun, Anunya Opasawatchai, Lisa Dratva, Lorenz Kretschmer, DENFREE Thailand, Anavaj Sakuntabhai, Pratap Singhasivanon, Swangjit Suraamornkul, Tawatchai Yingtaweesak, Khajohnpong Manopwisedjaroen, Nada Pitabut, Sasikanya Thaloengsok, Pattarakul Pakchotanon, Oranart Matangkasombut, Gavin Screaton, Wanwisa Dejnirattisai, Thaneeya Duangchinda, Jongeun Park, Kerstin Meyer, Juthathip Mongkolsapaya, Varodom Charoensawan, Sarah A. Teichmann, Ponpan Matangkasombut

**Affiliations:** 1 Department of Microbiology, Faculty of Science, Mahidol University, Bangkok, Thailand; 2 Integrative Computational BioScience (ICBS) Center, Mahidol University, Nakhon Pathom, Thailand; 3 Single-cell Omics and Systems Biology of Diseases (scSyBiD) Research Unit, Faculty of Science, Mahidol University, Bangkok, Thailand; 4 Chakri Naruebodindra Medical Institute, Faculty of Medicine Ramathibodi Hospital, Mahidol University, Samut Prakan, Thailand; 5 Research Department, Faculty of Medicine Siriraj Hospital, Mahidol University, Bangkok, Thailand; 6 Department of Oral Microbiology, Faculty of Dentistry, Mahidol University, Bangkok, Thailand; 7 Cambridge Stem Cell Institute, Jeffrey Cheah Biomedical Centre, Cambridge Biomedical Campus, University of Cambridge, Cambridge, UK; 8 Department of Medicine, University of Cambridge, Cambridge, UK; 9 Department of Microbiology and Center of Excellence on Oral Microbiology and Immunology, Faculty of Dentistry, Chulalongkorn University, Bangkok, Thailand; 10 Research Laboratory of Biotechnology, Chulabhorn Research Institute, Bangkok, Thailand; 11 CAMS Oxford Institute, Chinese Academy of Medical Sciences & Peking Union Medical College, University of Oxford, Oxford, UK; 12 Centre for Human Genetics, Nuffield Department of Medicine, University of Oxford, Oxford, UK; 13 Division of Emerging Infectious Disease, Research Department, Faculty of Medicine Siriraj Hospital, Mahidol University, Bangkoknoi, Bangkok, Thailand; 14 Molecular Biology of Dengue and Flaviviruses Research Team, National Center for Genetic Engineering and Biotechnology, National Science and Development Agency, NSTDA, Pathum Thani, Thailand; 15 Division of Dengue Hemorrhagic Fever Research, Faculty of Medicine, Siriraj Hospital, Mahidol University, Bangkok, Thailand; 16 Graduate School of Medical Science and Engineering, KAIST, Daejeon, Republic of Korea; 17 Wellcome Sanger Institute, Wellcome Trust Genome Campus, Hinxton, Cambridge, UK; 18 Mahidol-Oxford Tropical Medicine Research Unit, Faculty of Tropical Medicine, Mahidol University, Bangkok, Thailand; 19 Centre for Tropical Medicine and Global Health, Nuffield Department of Medicine, University of Oxford, Oxford, UK; 20 Division of Medical Bioinformatics, Research Department, Faculty of Medicine Siriraj Hospital, Mahidol University, Bangkok, Thailand; 21 Department of Biochemistry, Faculty of Medicine Siriraj Hospital, Mahidol University, Bangkok, Thailand; 22 Department of Biochemistry, Faculty of Science, Mahidol University, Bangkok, Thailand; 23 Siriraj Center of Research Excellence in Dengue and Emerging Pathogens, Faculty of Medicine Siriraj Hospital, Mahidol University, Bangkok, Thailand; 24 Siriraj Genomics, Faculty of Medicine Siriraj Hospital, Mahidol University, Bangkok, Thailand; 25 School of Chemistry, Institute of Science, Suranaree University of Technology, Nakhon Ratchasima, Thailand; 26 CIFAR Macmillan Multi-scale Human Programme, CIFAR, Toronto, ON Canada; 27 Institut Pasteur, CNRS UMR2000, Ecology and Emergence of Arthropod-borne Pathogens Unit, Paris, France; 28 Department of Tropical Hygiene, Faculty of Tropical Medicine, Mahidol University, Bangkok, Thailand; 29 Faculty of Medicine, Vajira Hospital, Bangkok, Thailand; 30 Thasongyang Hospital, Tak, Thailand; 31 Mahidol Vivax Research Unit, Faculty of Tropical Medicine, Mahidol University, Bangkok, Thailand; 32 Office of Research Services, Faculty of Tropical Medicine, Mahidol University, Bangkok, Thailand; 33 Faculty of Medicine, King Mongkut’s Institute of Technology Ladkrabang, Bangkok, Thailand; 34 Department of Bacterial and Parasitic Diseases, Armed Forces Research Institute of Medical Sciences, Bangkok, Thailand

**Keywords:** Viral host response, Viral infection, Cellular immunity, Dengue virus

## Abstract

Dengue virus (DENV) is a major global health threat, with secondary heterotypic infections potentially inducing detrimental memory immune responses. Antigen-specific CD8 + T cells contribute to both protection and pathogenicity, yet how their phenotypic heterogeneity relates to disease severity remains unclear. Here, we performed plate-based single-cell RNA sequencing of circulating DENV-specific CD8 + T cells identified by HLA tetramers loaded with DENV NS3-derived epitopes. Using tetramer binding to peptides corresponding to the currently and serologically inferred dominant previously infecting serotypes, we identify distinct CD8 + T cell subsets associated with disease severity. Asymptomatic dengue is enriched for lower tetramer binding cells with moderate cytotoxic programs, whereas dengue hemorrhagic fever is associated with high tetramer binding CX3CR1 + CD8 + T cells exhibiting enhanced expression of genes related to T cell receptor signaling and cytotoxicity. T cell receptor repertoires are similar among symptomatic cases but displayed temporal dynamics. Overall, DENV NS3-specific CD8 + T cells across disease severity and time are associated with distinct transcriptomic states and T cell receptor features.

## Abstract (web summary)

Using single-cell profiling of DENV-specific CD8 + T cells reactive to NS3 epitopes derived from different DENV serotypes, the authors identify distinct transcriptomic phenotypes, TCR features, and binding-associated states linked to disease severity.

## Introduction

Dengue is a major global health challenge, with an estimated 390 million infections annually, of which approximately 96 million present with clinical symptoms^1^. It is a mosquito-borne disease caused by dengue virus (DENV), which consists of four circulating serotypes (DENV1-DENV4)^2^. Infection with one serotype typically confers lifelong immunity against that specific serotype. However, reinfection with a different serotype carries a risk of severe dengue due to antibody-dependent enhancement (ADE)^2^. DENV infection manifests across a broad clinical spectrum, ranging from asymptomatic dengue (AD) to symptomatic disease (SD), which includes mild dengue fever (DF) and severe dengue hemorrhagic fever (DHF)^2,3^. Despite the promise of licensed live-attenuated dengue vaccines, some limitations persist. For instance, in dengue-naive children, CYD-TDV has been linked to an increased risk of hospitalization after primary infection^4^. Similarly, TAK-003, which employs a DENV2 backbone to enhance CD8 + T cell responses, has demonstrated suboptimal efficacy against certain serotypes^5^. Given these challenges, DENV vaccine development should prioritize strategies that mitigate antibody-enhanced disease severity while strengthening cellular immunity for all DENV serotypes^6^.

It has been shown that DENV-specific CD8 + T cells played a dual role in infection, potentially contributing both to protection and pathogenesis^7^. On the one hand, DENV-specific CD8 + T cells have been shown to play a role in controlling dengue virus infection in murine models^8,9^^,^. In humans, during the early phase of infection, these cells became fully activated with multifunctionality, migrating to inflamed tissues, and later differentiated into distinct memory T cell subsets, enabling immune responses for reinfection^10–12^. On the other hand, pathogenicity was also proposed during heterotypic infection, as cross-reactive memory T cells specific to a prior serotype expand instead of naive ones specific to the new serotype^13,14^. The weakly cross-reactive T cells resulted in suboptimal TCR signaling, reduced cytotoxicity, and an impaired ability to efficiently eliminate DENV-infected cells^13–15^. Furthermore, excessive inflammatory cytokine production might increase vascular permeability, exacerbating severe disease outcomes^16,17^. These findings underscore the complexity of DENV-specific CD8 + T cell responses, as shown in their serotype-specific and cross-reactive epitope recognition, functional dynamics, and contribution to disease outcomes.

Previously, we demonstrated that effector memory CD8 + T cells (TEM) underwent a significant expansion in AD, which may help our understanding of potential protective immune responses^18^. Although the majority of DENV infection is asymptomatic, acquiring samples from AD cases during active viremia remains challenging due to the lack of clinical manifestation^2,19^. The DENFREE cohort study in Thailand conducted an extensive effort to monitor household members (HHMs) of hospitalized dengue patients to detect AD cases^19^. Over five years, among 179 HHMs of 279 SD patients, only eight individuals were identified with viremia but without symptoms. Because symptom onset cannot be defined in these AD individuals, their stage of infection cannot be determined. Nonetheless, AD cases have been shown to exhibit delayed viral decay rates compared to SD ones, suggesting a negative feedback mechanism that compromises viral clearance while preventing clinical symptoms^19,20^. The heterogeneity of CD8 + T cells in the context of dengue has been investigated in multiple studies using single-cell RNA sequencing (scRNA-seq) of total peripheral blood mononuclear cells (PBMC)^18,21–24^. In AD, we observed that CD8 + T cells exhibited a gene expression profile with high cytokine production, strong cytotoxicity, and reduced exhaustion compared to SD cases^18^. Consistent with recent studies analyzing total circulating or peptide-stimulated CD8 + T-cell populations, early CD8 + T-cell dysregulation has been reported in SD, including the expansion of activated CD8 + TEM cells with inhibitory features and altered functional profiles^23,24^. These observations suggest that CD8 + T-cell responses characterized by enhanced effector functions and reduced dysfunction, as observed in AD, may contribute to protective immunity. Furthermore, cellular heterogeneity within CD8 + TEM cells and their dynamic expansion were observed during DENV infection^21^. However, previous studies were done based on total CD8 + T cells, and characterization of antigen-specific CD8 + T cells remains to be further investigated.

DENV epitopes for CD8 + T cells have been shown to predominantly localize to NS proteins, particularly NS3, NS4, and NS5, regardless of geographical infection sites or HLA restriction^10,17^. Despite the abundance of epitopes, only a few have been shown to be immunodominant^11–14,17,25,26^ and well characterized. For example, HLA-A*11-restricted NS3_133_ epitope variants have been shown to drive selective TCR gene usage, shaping cross-serotype T cell activation, dynamics, and cytokine responses^13,25,26^. Similarly, NS3_556_ variants restricted to HLA-A*24 have been demonstrated to skew immune responses in epitope variant binding, though prior variants showed suboptimal T cell function^14^. However, most phenotypic characterizations of DENV-specific CD8 + T cells have been conducted using samples from the convalescent phase^10,14,17^, which may not capture the heterogeneity and functional states of these cells during the acute phase, when disease severity is determined.

In this study, we used HLA tetramer technology and index cell sorting in combination with a plate-based scRNA-seq (Smart-seq2) platform to characterize the heterogeneity of DENV NS3-specific CD8 + T cell responses in natural human DENV infections with varying disease outcomes. This approach enabled us to investigate the relationship between relative TCR avidity, V(D)J gene usage, binding of CD8 + T cells to HLA tetramers loaded with NS3 peptides (pHLA) from different DENV serotypes, HLA restriction, and T cell function, as assessed through gene expression profiling and correlation with disease severity. We analyzed clinical PBMC samples from the DENFREE cohort, including rare DENV viremic AD cases, DF, and DHF in both the acute and convalescent phases, with a focus on HLA-A*11 and HLA-A*24, which are predominant in the Southeast Asian population^13^. Moreover, by integrating time-course PBMC profiling datasets and serum protein from a partially overlapped cohort, we leveraged DENV NS3-specific TCR CDR3 amino acid sequence similarity to infer potential DENV NS3-specific CD8 + T cells in the entire CD8 + T cells, their predicted cell-cell communication to other immune cells and cytokine correlation.

## Results

### Study design and experimental overview

**Fig. 1:**
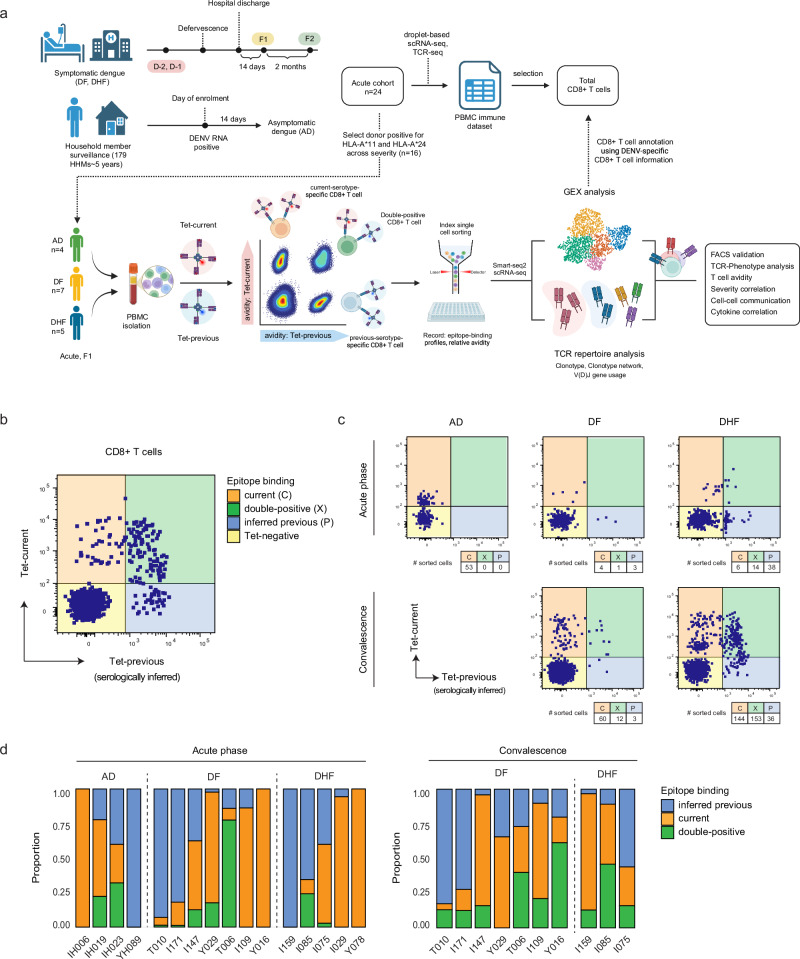
Study design and classification of CD8 + T cell binding to DENV-NS3 epitopes across infection phases and disease severity. **a** Schematic of the experimental workflow. Participants with natural DENV infection were enrolled during the acute phase (D-2/D-1), 2 weeks post-fever (F1), or 2 months post-fever (F2), and classified by disease severity (AD, DF, DHF). PBMCs were stained with tetramers targeting immunodominant DENV NS3 epitopes (GTS or NYA), followed by index sorting of tetramer-positive CD8 + T cells. Single-cell transcriptome and TCR profiling were performed using Smart-seq2. Integrated analysis included clustering, transcriptional profiling, surface marker validation, TCR sequence analysis, and correlation with clinical parameters. Created in BioRender. Srikor, S. (2026) https://BioRender.com/7fbtv7e. **b** Classification strategy of CD8 + T cells based on tetramer binding. Cells were assigned to four categories: CD8 + T cell binding to peptide derived from serologically inferred previously infecting serotype (blue), CD8 + T cell binding to peptide derived from the currently infecting serotype (orange), double-positive (green), and tetramer-negative (yellow). **c** Scatter plots showing distributions of tetramer-positive CD8 + T cells classified by pHLA tetramer binding category across acute and convalescent phases in each clinical group (AD, DF, DHF). Each plot overlays pre-sorted CD8 + T cells (yellow, tetramer-negative) with the sorted tetramer-positive population from the same sample. The pHLA tetramer binding patterns varied by disease severity and infection stage. The number of sorted cells for each epitope-binding class is indicated in the box below. **d** Stacked bar plots showing proportions of pHLA tetramer binding categories (inferred previous, current, double-positive) within tetramer-positive CD8 + T cells, stratified by epitope and disease severity. Left: acute-phase samples (AD, *n* = 4; DF, *n* = 7; DHF, *n* = 5). Right: convalescent-phase samples (DF, *n* = 7; DHF, *n* = 3).

To investigate DENV NS3-specific CD8 + T cells across different severities and timepoints in natural dengue infection, PBMC samples from DENV-infected individuals with HLA-A*11 and HLA-A*24 were analyzed. The samples comprised acute-phase samples from four AD, seven DF, and five DHF cases, as well as those from a 2-week follow-up based on seven DF and three DHF cases. No significant differences in viremia, age, or sex were observed among the groups (Supplementary Fig. 1). DENV NS3-specific CD8 + T cells were identified using HLA-A*11 and HLA-A*24 tetramers loaded with GTS (NS3_133–142_) and NYA (NS3_556–564_) epitopes, respectively. These pHLA tetramers, conjugated to different fluorochromes (PE or APC), were used to stain CD8 + T cells binding to both of the currently infecting and serologically inferred dominant previously infecting DENV serotypes. In this study, the currently infecting serotype was determined by qPCR, while the serologically inferred dominant previously infecting serotype was defined as the non-current serotype with the highest plaque reduction neutralization test (PRNT) titer (Supplementary Table 2). Individual tetramer-positive cells were index-sorted into 96-well plates for subsequent Smart-seq2-based scRNA-seq (SS2), with fluorescent intensity as a proxy for TCR avidity, as tetramer staining intensity has previously been shown to reflect the overall strength of TCR-pMHC interactions and CD8 co-receptor engagement^27–29^. A total of 2397 DENV NS3-specific CD8 + T cells were characterized across severity outcomes and timepoints via tetramer staining, with unstained pMHCs (Supplementary Fig. 2a–c), as well as HLA-A*11- and HLA-A*24-negative individuals (Supplementary Fig. 3a, b), serving as negative controls. This enabled joint analyses of CD8 + T cell transcriptomes, TCR features, epitope binding, overall TCR binding strength, and their association with disease severities. Additionally, integration with previously published droplet-based scRNA-seq data^18^ from total CD8 + T cells and PBMCs from the same samples at both acute and follow-up phases, as well as cytokine array data^30^ from the same cohort, provided a broader view of cell interactions, cytokine profiles, and immune responses in dengue infection (Fig. 1a).

### CD8 + T cell binding to NS3 peptides from different DENV serotypes across infection phases and disease severity

To investigate patterns of CD8 + T cell binding to NS3 peptides from different DENV serotypes in relation to disease severity and progression, we sorted CD8 + T cells based on binding to HLA tetramers loaded with NS3 peptides corresponding to the currently infecting serotype, as defined by qPCR, and the serologically inferred dominant previously infecting DENV serotype. This enabled us to assess their differential avidity, transcriptomic profiles, and TCR sequences in relation to disease severity and disease course. Single-positive staining indicated CD8 + T cells binding tetramers loaded with NS3 peptides from one DENV serotype, whereas double-positive staining identified cells binding tetramers loaded with NS3 peptides derived from both tested DENV serotypes (Fig. 1b). During the acute phase, no associations between patterns of CD8 + T cell binding to NS3 peptides derived from different DENV serotypes and disease severity were observed, with variability across individual samples (Fig. 1c, d left). Notably, one DF case exhibited a dominant double-positive population (Fig. 1d). During convalescence in SD samples, the overall abundance of DENV NS3-specific CD8 + T cells, including both single-positive and double-positive populations, was generally higher than in the acute phase (Fig. 1c, d right). Our findings reveal dynamic changes in total DENV NS3-specific CD8 + T cell binding to NS3 peptides from the currently and the serologically inferred dominant previously infecting DENV serotype over the course of infection, with no significant association between the abundance of cells binding these peptides and disease severity.

### Characterization of DENV NS3-specific CD8 + T cell subsets

**Fig. 2:**
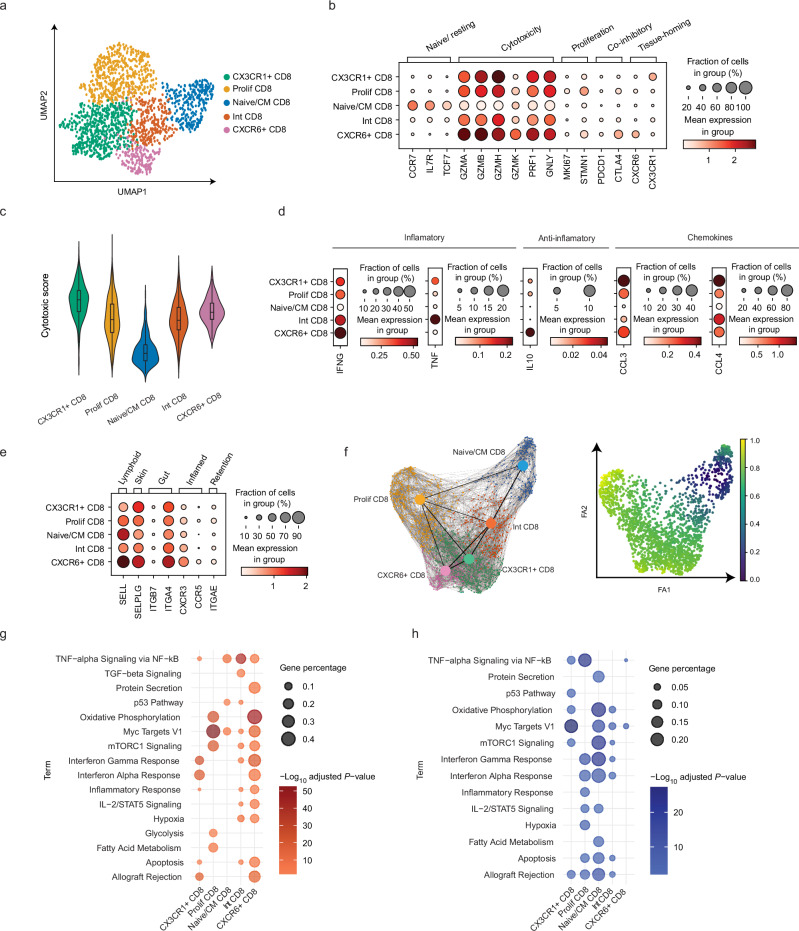
Transcriptional and functional heterogeneity of DENV NS3-specific CD8 + T cell subsets. **a** UMAP of total CD8 + T cells from tetramer-positive populations across donors, colored by subset identity. Five transcriptionally distinct clusters were identified: CX3CR1 + CD8, Prolif CD8, Naive/CM CD8, Int CD8, and CXCR6 + CD8. **b** Dot plot showing subset-specific expression of canonical gene signatures related to naïve/memory state, cytotoxicity, proliferation, and inhibitory/exhaustion markers. Dot size represents the percentage of cells expressing the gene; color intensity indicates average expression. **c** Violin plots depicting the score of cytotoxicity-related gene set across CD8 + T cell subsets. Distributions are shown at the single-cell level. Box plots indicate the median (center line), interquartile range (box), and whiskers extending to 1.5× the interquartile range. **d** Dot plots illustrate the expression level of inflammatory, anti-inflammatory, and chemotaxis-associated genes across subsets, showing preferential enrichment of inflammatory and chemokine transcripts in CX3CR1 + CD8 cells. **e** Dot plots illustrate the expression level of tissue-homing-related genes (**f**) Pseudotime and trajectory inference on total CD8 + T cells. **g**, **h** Over-representation analysis (ORA) of differentially expressed genes against MSigDB gene sets. Statistical significance was assessed using a one-sided Fisher’s exact test, with *p*-values adjusted for multiple comparisons using the Benjamini–Hochberg method (adjusted *p*-value < 0.05). Dot size indicates gene set overlap, and color represents –log_10_ adjusted *p*-value.

Given the limitations of pHLA tetramer and flow cytometry-based approaches in capturing the full heterogeneity of DENV NS3-specific CD8 + T cells, we used single-cell transcriptomic analysis to evaluate their subsets and potential associations with disease severity. This approach revealed five subsets of DENV NS3-specific CD8 + T cells based on their single-cell gene expression profiles (Fig. 2a). The Naive/Tcm subset (Naive/CM CD8) expressed high levels of naive/resting T cell markers, including *CCR7, IL7R, TCF7*, while the other remaining subsets were enriched for effector-associated genes, such as *GZMB, PRF1, GNLY*. Notably, a cluster of proliferating CD8 + T cells (Prolif CD8) exhibited elevated expression of proliferation markers (*MKI67, STMN1*). We also identified a cluster of CXCR6 + CD8 + T cells (CXCR6 + CD8), highly expressed *CXCR6* previously identified as a circulation-to-tissue homing marker^31,32^, with greater expression of co-inhibitory receptor genes, *PDCD1, CTLA4*. Additionally, a cluster of CX3CR1 + CD8 + T cells (CX3CR1 + CD8) was found, with dominant *CX3CR1* expression, marked for circulating and terminally differentiated effector/memory CTLs^33^, and high *GZMB, GZMH,* but low *GZMK* expression. Finally, a cluster of intermediate CD8 + T cells (Int CD8) displayed intermediate cytotoxic gene expression while retaining expression of naive/resting markers (Fig. 2b and Supplementary Fig. 4a).

To systematically quantify the overall cytotoxic potential of DENV NS3-specific CD8 + T cell subsets, we applied a gene module scoring approach, using a cytotoxic gene signature derived from the activated CD8 + T cell cytotoxic module, as identified through scRNA-seq analysis of healthy human tissues^34^, we found the highest cytotoxic scores in CX3CR1 + CD8, moderate scores in CXCR6 + CD8, Prolif CD8 and Int CD8 clusters, and the lowest scores in the Naive/CM CD8 cluster (Fig. 2c). Investigation of cytokine and chemokine gene expression across the CD8 + T cell subsets showed that the CXCR6 + CD8 cluster had the highest expression of *IFNG*, while the Int CD8 cluster prominently expressed *TNF*. Notably, CXCR6 + CD8 cells also expressed *IL10*. In contrast, the CX3CR1 + CD8 subset was characterized by elevated expression of chemokines *CCL3* and *CCL4*, which are associated with leukocyte recruitment^35^ (Fig. 2d).

Next, we analyzed the expression of genes associated with tissue-specific localization. *SELPLG*, which encodes the skin-homing marker CLA and has been previously linked to CD8 + T cells during DENV infection^12^, showed differential expression across subsets. The Naive/CM and Int CD8 clusters showed moderate expression of peripheral homing markers, including *SELPLG* and *ITGA4*, suggesting a more central or circulating phenotype. In contrast, the CXCR6+ and Prolif CD8 clusters were enriched for tissue/inflammatory site-homing markers such as *SELL* (encoding CD62L), *SELPLG*, *ITGA4*, *CXCR3* and *CCR5*, suggesting they might be primed to migrate to inflamed peripheral tissues, particularly skin as sites of viral entry^2^. In contrast, the CX3CR1 + CD8 cluster, while maintaining high *SELPLG* and *CX3CR1* expression, showed reduced *CXCR3* and *CCR5* levels, indicating a potentially distinct migratory profile, possibly associated with blood-tissue surveillance rather than inflammation-driven recruitment (Fig. 2e).

Given the differential gene expression indicative of a spectrum of cellular phenotypes demonstrated earlier, we conducted trajectory analysis of CD8 + T cell subsets to explore their transcriptional relationships. The Naive/CM subset was positioned at one end, with effector subsets such as CXCR6 + , Prolif, and CX3CR1 + CD8 + T cells located toward the opposite end. Notably, the Int CD8 cells occupied the middle space, suggesting they share features with both early and late cellular states. These findings support a model of divergent differentiation pathways, where distinct effector subsets may represent alternative terminal fates of DENV NS3-specific CD8 + T cells. This suggests functional specialization may occur through multiple differentiation lineages, rather than along a single linear continuum (Fig. 2f).

To define additional biological characteristics of each subset, we performed an overrepresentation analysis (ORA) of differentially expressed genes. The Naive/CM CD8 subset showed downregulation of cytokine-responsive pathways (*IFITM3, IFITM2, IRF7*), CD8 + T cell response signatures represented as allograft rejection *(ITGB2, PRF1, GZMB*), and oxidative phosphorylation, consistent with a resting phenotype, while the Int CD8 subset was enriched for TGF-β signaling (*KLF10, JUNB, SMAD7*) and TNF-α/NF-κB signaling pathways (*BTG2, PFKFB3, TNFAIP3*). Among the three distinct effector subsets, CX3CR1+ and CXCR6 + CD8 subsets were enriched for interferon responses (*IFITM2, IFITM3, IRF7*), cytotoxic pathways and T cell signaling programs, while CXCR6+ and Prolif CD8 clusters also showed enrichment for oxidative phosphorylation (*SCA1, COX7B*). Additionally, Prolif CD8 subsets were uniquely enriched for mTORC1 signaling, Myc targets, and glycolysis, hallmarks of active metabolic reprogramming and immune activation (Fig. 2g–h).

Overall, DENV NS3-specific CD8 + T cells span a spectrum of functional states. The Naive/CM CD8 cluster exhibited a quiescent phenotype with low expression of cytotoxic genes, even with antigen specificity during active infection. The Int CD8 subset displayed genes associated with moderate cytotoxic potential and elevated TNF signaling. The gene expression patterns in the CXCR6+ and Prolif CD8 clusters were moderate cytotoxic, metabolically active and enriched for inflammatory and tissue-homing signatures. In contrast, the CX3CR1 + CD8 cluster exhibited genes related to high cytotoxicity, cytokine production and circulation-based immune surveillance. These gene signatures reveal distinct immune profiles, metabolic activities, and signaling pathways of DENV NS3-specific CD8 + T cell subsets, shedding light on their potential roles in dengue infection.

### Association between DENV NS3-specific CD8 + T cell subsets and disease severity

**Fig. 3:**
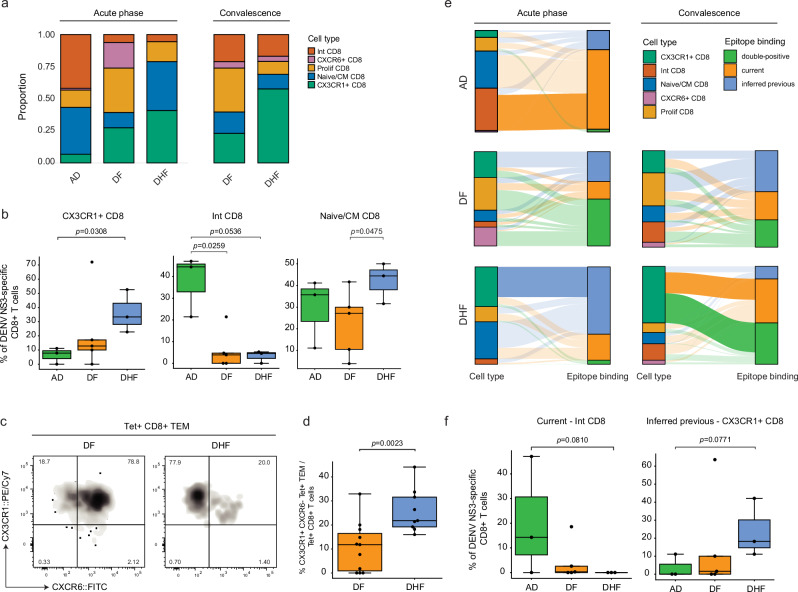
DENV NS3-specific CD8 + T cell subset compositions were associated with disease severity and epitope binding profile. **a** Stacked bar plots showing the distribution of DENV NS3-specific CD8 + T cell subsets across disease severity groups during the acute (left) and convalescent (right) phases. **b** Quantification of CX3CR1 + CD8, Int CD8, and Naive/CM CD8 subsets among tetramer-positive DENV NS3-specific CD8 + T cells across disease severity groups during the acute phase (AD, *n* = 3; DF, *n* = 5; DHF, *n* = 3). Statistical comparisons were performed using the Kruskal–Wallis test with Dunn’s multiple comparisons. **c** Representative flow cytometry plots showing CX3CR1 and CXCR6 co-expression within tetramer-positive CD8 + T cells in CD8 + TEM population from DF and DHF patients. Quadrant frequencies of tetramer-positive CD8 + T cells are indicated relative to the parental CD8 + TEM population. **d** Quantification of CX3CR1 + CXCR6 + DENV NS3-specific CD8 + T cells in clinical dengue cases (DF, *n* = 11; DHF, *n* = 9). Statistical comparisons were performed using the two-tailed Mann–Whitney *U*-test. **e** Sankey diagrams linking CD8 + T cell subsets (left) to pHLA tetramer binding categories (right; inferred previous, current, double-positive) across AD, DF, and DHF groups during the acute and convalescent phases. **f** Quantification of DENV NS3-specific CD8 + T cells, stratified by subset and DENV-NS3 epitope binding profiles: Int CD8 binding to current-serotype derived epitope (left) and CX3CR1 + CD8 binding to inferred previous-serotype derived epitope (right), across severity outcomes (AD, *n* = 3; DF, *n* = 5; DHF, *n* = 3). One AD sample was excluded due to quality control failure, leaving three AD samples for analysis. Quantification analysis was restricted to samples with at least four DENV NS3-specific cells. Statistical comparisons were performed using the Kruskal–Wallis test. Source data is provided as a Source Data file.

We then investigated whether the identified DENV NS3-specific CD8 + T cell subsets are correlated with clinical outcomes. During acute infection, CX3CR1 + CD8 + T cells increased with disease severity, while Int CD8 + T cells were more abundant in AD but less prevalent in DF and DHF (Fig. 3a left). Notably, the proportion of CX3CR1 + CD8 T cells increased with disease severity, being significantly higher in DHF compared to AD. Conversely, Int CD8 + T cells were significantly more abundant in AD than in DF, with a trend toward higher levels compared to DHF. Naive/CM CD8 + T cells also showed an increase in DHF, reaching significantly higher levels than in DF (Fig. 3b). Additionally, the high proportion of CX3CR1 + CD8 + T cells was also seen in the convalescent phase, especially in DHF (Fig. 3a right). However, no significant differences in CD8 + T cell subset distribution were observed during the convalescent phase (Supplementary Fig. 5a).

To independently validate the enrichment of the CX3CR1 + CD8 + T cell subset in SD individuals, we analyzed pHLA tetramer-positive CD8 + T cells by flow cytometry. The results showed that CX3CR1 + CXCR6 − TEM cells, defined by CD3 + CCR7− CD45RO + CD45RA− CD8+ (Supplementary Fig. 5b), were significantly more abundant in DHF during the acute phase (Fig. 3c, d). These findings suggest that excessive CX3CR1 + CD8 + T cells may contribute to severe dengue pathogenesis through their high cytotoxicity and patrolling function in circulation, potentially associated with vascular leakage in severe dengue. In contrast, the Int CD8 + T cells enriched in AD moderate cytotoxicity and TNF signaling, which may support protective immunity without triggering immunopathology (Fig. 3b). The balanced proportion of CD8 + T cell subsets may be a key factor in dictating disease severity outcome.

### CD8 + T cell subsets show distinct patterns of binding to NS3 peptides derived from different DENV serotypes across disease severity

As no significant association of total DENV NS3-specific CD8 + T binding to NS3 epitopes from different DENV serotypes and disease severity outcomes was observed (Fig. 1d), we investigated whether the defined DENV NS3-specific CD8 + T cell subsets differed in their binding to NS3 peptides from different DENV serotypes in relation to disease severity. Notably, during the acute phase, the Int CD8 subset in AD cases showed a trend toward binding more to NS3 epitopes derived from the currently infecting serotype, although this was not statistically significant (Fig. 3e left). In DHF, the CX3CR1 + CD8 subset was biased toward binding to NS3 epitopes derived from serologically inferred dominant previously infecting DENV serotypes, and its proportion was higher than in AD. While not statistically significant, this trend suggests a memory response that may contribute to severe disease. (Fig. 3e left-f). Their high cytotoxic potential and circulation-homing profile during the acute phase, which may contribute to immunopathogenesis such as vascular leakage, could be driven by heterotypic responses due to immune imprinting. Interestingly, during the convalescent phase, CX3CR1 + CD8 + T cell population continued to dominate in DHF cases and included cells with single-positive binding to the NS3 peptide from the currently infecting serotype, as well as double-positive cells binding both NS3 peptides tested (Fig. 3e right). Taken together, these findings suggest that DENV NS3-specific CD8 + T cell subsets show different patterns of binding to NS3 peptides from different DENV serotypes across disease severity.

### Differential TCR avidity, signaling and co-regulation across DENV NS3-specific CD8 + T cell subsets

**Fig. 4:**
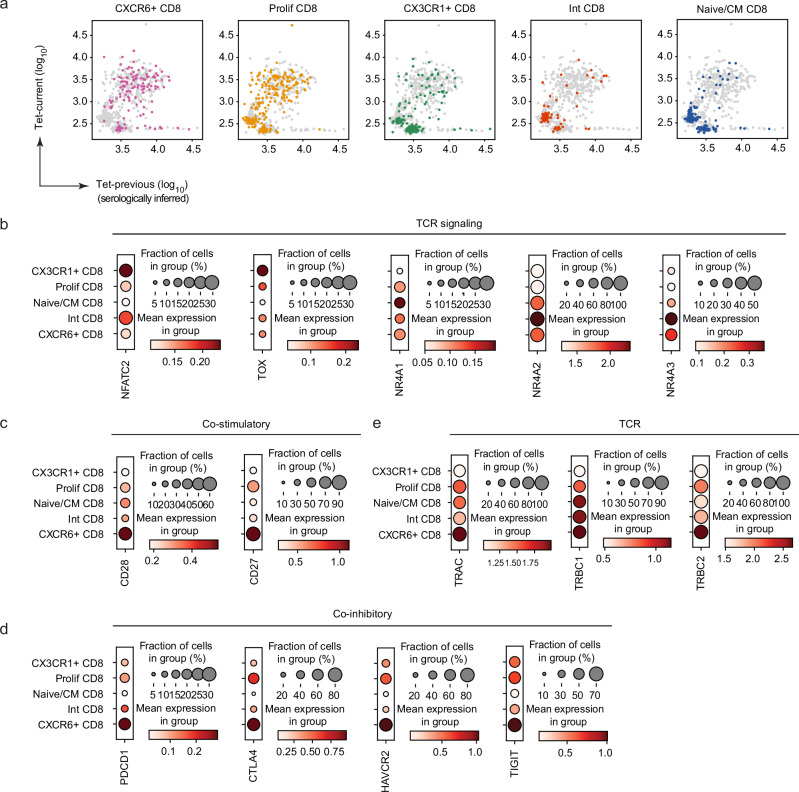
DENV NS3-specific CD8 + T cell subsets exhibited distinct expression of TCR signaling, costimulatory, and inhibitory molecules during the acute dengue infection. **a** Scatter plots showing fluorescence intensities of tetramer staining (*x*-axis: Tet-inferred previous; y-axis: Tet-current), stratified by CD8 + T cell subsets. **b**–**e** Dot plots summarizing gene expression patterns across CD8 + T cell subsets. In these plots, the size of each dot represents the fraction of cells within a subset expressing the gene, and the color intensity reflects the mean expression level. **b** Genes involved in TCR signaling pathways. **c** Co-stimulatory molecules. **d** Co-inhibitory molecules. **e** TCRα/β constant region genes.

Based on the distinct epitope binding of DENV NS3-specific CD8 + T cell subsets, we hypothesized that differences in TCR-pHLA avidity may shape their functional profiles. Using pHLA tetramer fluorescence intensity as a proxy for TCR avidity, consistent with previous studies^27–29^, we found that during acute infection, high-avidity CD8 + T cells were significantly enriched in CXCR6+ and CX3CR1 + CD8 subsets, compared to other subsets (Fig. 4a and Supplementary Fig. 6). ORA results revealed that CX3CR1+ and CXCR6 + CD8 were enriched for pathways related to TCR signaling and T cell activation (Supplementary Fig. 4b, c). To assess whether TCR avidity is associated with phenotypic diversity through differential TCR signaling, we examined the expression of transcription factors previously linked to TCR signal strength and duration, including members of the NFAT and NR4A families^36,37^. CX3CR1 + CD8 expressed high levels of *NFATC2* (encoding NFAT1) and *TOX* which marked cytotoxic effector and memory CD8 + T cells with active functional potential^38^, but relatively low levels of *NR4A2* and *NR4A3* (Fig. 4b). This transcriptional profile may reflect a state shaped by strong but not sustained TCR stimulation, sufficient to induce *NFATC2* and *TOX*, but insufficient to maintain the NFAT1-dependent signaling required for *NR4A2* and *NR4A3* expression^36^. Supporting this interpretation, CX3CR1 + CD8 exhibited low expression of TCR constant region genes (Fig. 4e), despite showing relatively high tetramer binding, implying that high-avidity stimulation may have led to TCR downregulation in this subset^39^. These results suggest that CX3CR1 + CD8 might represent an imprinted memory population primed by high-avidity signals, enabling rapid recall of cytotoxic function without requiring sustained TCR engagement.

In contrast, the Int CD8+ subset exhibited higher expression of *NR4A2, NR4A3*, and *NFATC2* (Fig. 4b), consistent with a potentially distinct TCR signaling context that may involve more prolonged or repeated stimulation^36,37^, even with their relatively lower TCR avidity. CXCR6 + CD8 showed intermediate levels of these transcription factors (Fig. 4b), while maintaining higher expression of TCR constant region genes and co-expressing the highest levels of both co-stimulatory and co-inhibitory receptors (Fig. 4c–e). This combination suggests that TCR signaling in this subset may be modulated through the integrated influence of co-regulatory molecules. For comparison, Naive/CM CD8 expressed higher levels of *NR4A1*, which has been associated with relatively lower levels of TCR engagement^36^. Collectively, these findings suggest that TCR avidity, differential TCR signaling, TCR gene expression, and co-regulatory receptor expression may contribute to shaping the functional diversity of DENV NS3-specific CD8 + T cell subsets.

### TCR repertoire analysis reveals distinct TCR gene usage across disease severity

**Fig. 5:**
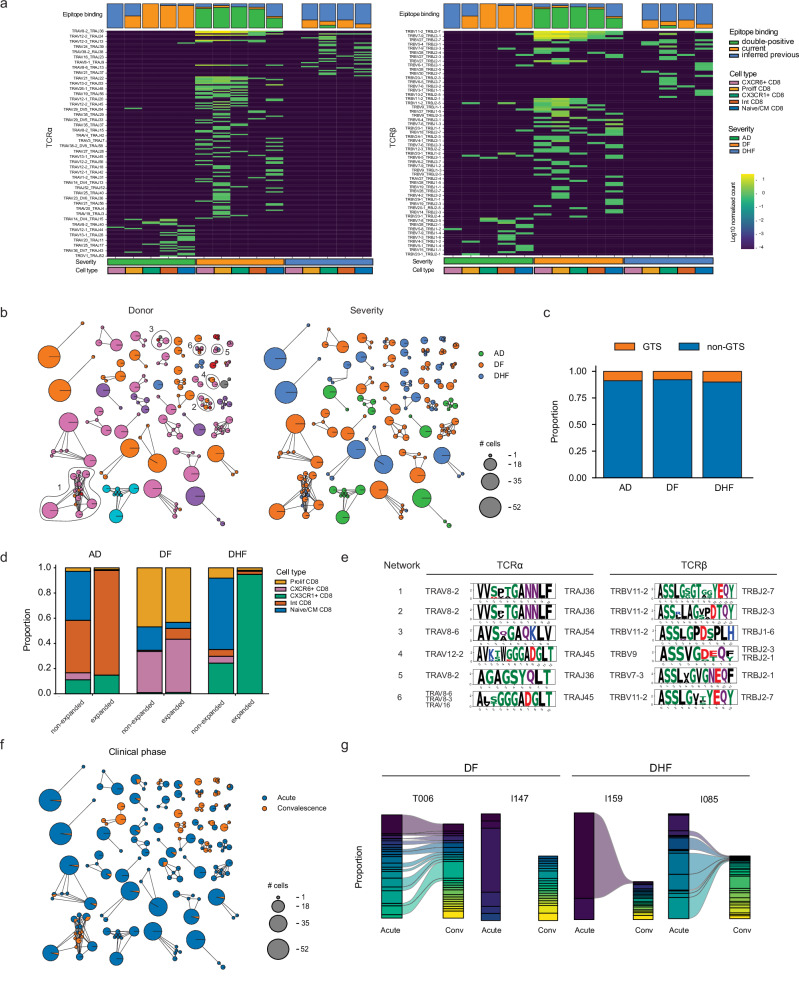
Clonality and epitope binding-associated features of TCR repertoires in GTS-specific CD8 + T cells across disease severities and infection phases. **a** Heatmaps showing paired TCRα (left) and TCRβ (right) usage across GTS-specific CD8 + T cells from the SS2 platform, stratified by epitope binding group (inferred previous, current, double-positive), cell type, and disease severity. Each row represents a unique paired V-J gene. **b** TCR similarity networks based on CDR3β sequences visualized by donor (left) and disease severity (right). Each node represents a unique TCR clonotype, with edges connecting clonotypes with high sequence similarity. Node size indicates clonal expansion (number of cells). **c** Stack bar plot showing the proportion of GTS-specific TCRs versus non-GTS among total CD8 + T cells across severities. **d** Subset distribution of GTS-associated clonotypes across clonal size and disease severities during the acute phase. **e** TCR sequence logos showing conserved CDR3 motifs among dominant public TCRα and TCRβ networks. **f** TCR similarity network colored by infection phase (acute vs. convalescent). **g** Proportions of GTS-specific CD8 + T cells showing clonal dynamics in individual donors across infection phases, with ribbons connecting identical clones.

Next, we investigated the TCR gene usage of DENV NS3-specific CD8 + T cell clones by disease severity. We focused on the analysis of pHLA-A*11-restricted cells due to limited sequence quality in HLA-A*24 samples. A total of 875 paired TCRα and TCRβ sequences from GTS-specific CD8 + T cells were analyzed, and we observed distinct patterns of TCR gene usage of TCRα and TCRβ emerged across CD8 + T cell subsets within each severity outcome (Fig. 5a). Intriguingly, AD displayed a unique TCR gene of both TCRα (Fig. 5a left) and TCRβ (Fig. 5a right) usage compared to SD, suggesting that the selection of VJ gene specific to the same GTS-HLA complex might be potentially associated with differences in the outcome of the infection. In contrast, DF and DHF cases showed three distinct patterns of VJ gene usage: DF-unique, DHF-unique, and shared between DF and DHF, observed in both the acute and convalescent phases (Fig. 5a and Supplementary Fig. 7). Notably, VJ cluster distribution was significantly associated with disease severity (Supplementary Fig. 8a–d). The overlapping V(D)J gene usage observed in DF and DHF, including predominant use of *TRBV11-2* and *TRBV9*, which contribute to the CDR2β loop as previously reported^26^ might predispose to severe outcomes. Conversely, AD cases used a different set of V(D)J genes, such as *TRVB6-6* and *TRVB20-1* (Supplementary Fig. 9a), which do not contain asparagine at the 58 position on the CDR2β loop, a residue previously shown to be important for GTS binding across DENV variants^26^. Unsupervised clustering further revealed that TCR repertoires were generally segregated by disease outcome (Supplementary Fig. 9b), consistent with principal component analysis (PCA) of global TCR repertoire composition, where resulting clusters showed significant association with AD and SD labels (Supplementary Fig. 10), suggesting an association between TCR gene usage and dengue severity. Moreover, TCR epitope binding in AD had a trend toward currently infecting serotypes, whereas in DHF, TCRs preferentially targeted serologically inferred dominant previously infecting serotypes, regardless of CD8 + T cell subset (Fig. 5a). This emphasizes how TCR composition based on infection history may reflect differences in CD8 + T cell functional responses and disease severity.

### Integrated multi-platform T cell clonality reveals a private network linked to GTS-specific CD8 + T cell functional subsets in the severity group and clonal dynamic shifts during the infection

We then characterized the TCR repertoire of GTS-specific CD8 + T cells across different subsets, disease severity outcomes, and infection phases. Clonotype network analysis based on high similarity of CDR3 amino acid sequences revealed predominantly private networks unique to severity groups, with certain sharing between DF and DHF (Supplementary Fig. 9c, d). However, we found a limited number of clonally expanded CD8 + T cells in AD, possibly due to the small sample size in SS2 data. Thus, we extended our analysis to total CD8 + T cells using matched donors in 10X scRNA-seq data (Fig. 1a). Integrating 10X and SS2 data allowed us to recover nucleotide-based clonotypes that were undetectable by tetramer staining alone (Supplementary Fig. 11a, b). Moreover, clonotype network analysis also identified additional potential GTS-specific CD8 + T cells within the total CD8 + T cell population, defined by high amino acid sequence similarity and shared networks with known GTS-specific CD8 + T cells. Consistent with earlier findings, most clonotype networks remained unique to specific severity groups, particularly AD, and a few ones were composed of cells from multiple donors of SD (Fig. 5b). Through this integration, inferred GTS-specific CD8 + T cells were identified within the total CD8 + T cell population in the 10X scRNA-seq data, accounting for approximately 0.2-30% across donors (Supplementary Fig. 11c), consistent with the established immunodominance of these epitopes in HLA-A*11-restricted dengue responses^11–13,17,25,26^. However, no differences were observed across disease severities during the acute phase, underscoring the immunodominance of the GTS epitope in the CD8 + T cell response (Fig. 5c and Supplementary Fig. 11d).

Further cell-type analysis of the inferred GTS-specific CD8 + T cells in the integrated dataset confirmed the identities of previously described DENV NS3-specific CD8 + T cell subsets. Consistent with the findings from the sorted DENV NS3-specific CD8 + T cells (Fig. 3a, b), inferred GTS-specific clonally expanded in AD were biased toward the Int CD8 + T cell subset, while those in DHF showed a bias toward CX3CR1 + CD8 + T cells (Fig. 5d). In contrast, the DF group exhibited greater cellular heterogeneity (Fig. 5d). These results further highlight the association between TCR repertoire features and CD8 + T cell phenotypes across disease severity outcomes.

As we found a few clonotype networks to be composed of cells from multiple SD donors (Fig. 5b), we performed an analysis of CDR3 motifs of TCRα and TCRβ from these clonotype networks shared across at least 3 donors and identified conserved V(D)J gene usage and amino acid motifs, aligning with previously described features of DENV-reactive TCRs^26^ (Fig. 5e). This supports the existence of public TCR motifs found in SD donors that may recognize shared DENV epitopes across individuals, suggesting that specific TCR features may underlie SD dengue across donors and the SD groups.

Next, dynamic shifts in the clonal composition of GTS-specific CD8 + T cells were investigated across the acute and convalescent phases, revealing both shared and distinct T cell clones over time (Fig. 5f). Dominant clones present during the acute phase often contracted by the convalescent phase, whereas other clones expanded specifically during the later phase (Fig. 5g). We hypothesized that early responders may be high-affinity CD8 + T cells that mount robust initial response, while most clones found in the convalescent phase may be of lower affinity that emerge later, as previously proposed^40^. These findings suggest that distinct CD8 + T cell clones may contribute to immune responses at different stages of infection. Clones dominant in the acute phase may influence disease severity, whereas those emerging in the convalescent phase might predispose to the outcome of the next infection.

### Integration of multi-platform analysis reveals phenotypic heterogeneity of CD8 + T cells and dynamics across severity outcomes and time

**Fig. 6:**
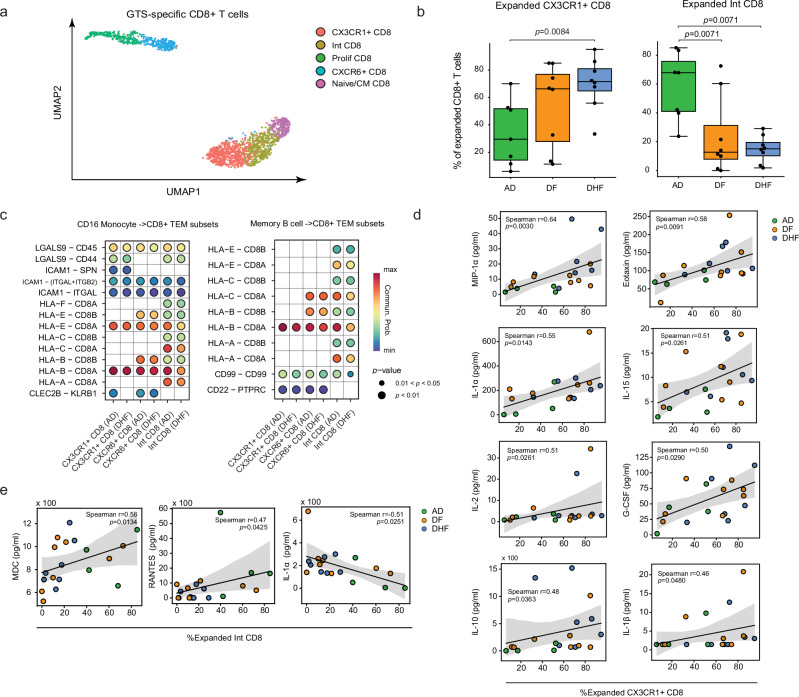
CD8 + T cell subsets and their association with inflammatory cytokines and immune cell crosstalk. **a** UMAP of GTS-specific CD8 + T cells colored by subset identity. Unsupervised clustering identified five transcriptional subsets: CX3CR1 + CD8, Int CD8, CXCR6 + CD8, Naive/CM CD8, and Prolif CD8. **b** Quantification of the proportion of expanded clones within CX3CR1+ and Int CD8 + T cells across disease severity groups in the 10X dataset (AD, *n* = 7; DF, *n* = 8; DHF, n = 8). One AD donor was excluded due to the absence of expanded CD8 + T cell clones. Statistical comparisons were performed using the Kruskal–Wallis test, with p-values indicated. **c** Cell–cell communication heatmaps inferred by CellChat showing significant predicted ligand–receptor interactions between CD8 + T cell subsets (rows) and either CD16+ monocytes (left) or memory B cells (right). Statistical significance was assessed using a one-sided permutation test implemented in CellChat. Dot size indicates significance level (*p*-value), and color represents normalized communication strength. **d**, **e** Correlations between the proportions of expanded CD8 + T cell subsets and serum cytokine concentrations from match donors (AD, *n* = 4; DF, *n* = 8; DHF, *n* = 7). **d** Expanded CX3CR1 + CD8 + T cells. **e** Expanded Int CD8 + T cells. Each dot represents an individual donor, colored by disease severity group. Spearman’s *r* and *p*-values are indicated. Linear regression lines with 95% confidence intervals are shown in gray. Source data is provided as a Source Data file.

In our previous analysis, we integrated GTS-specific CD8 + T cells from the 10X dataset with sorted DENV NS3-specific CD8 + T cells and performed unsupervised clustering on the integrated data. This approach enabled us to identify, overall, 10X derived, CD8 + T cell clusters that aligned with SS2-based gene expression markers (Fig. 6a and Supplementary Fig. 12a, b). While prior studies have attempted to distinguish CD8 + T cell subsets coarsely across severity outcomes^18,21^, our integrated analysis further dissected the heterogeneity of total CD8 + T cells across 24 samples present in the total PBMC dataset. Notably, we observed a trend toward higher abundance of CX3CR1 + CD8 + T cells in DHF, whereas Int CD8 + T cells differed significantly across severity groups (*p* = 0.0056) and were enriched in AD compared to DHF (Supplementary Fig. 12c). To refine our analysis, we focused on clonally expanded CD8 + T cells, which may be enriched for DENV-specific populations, though we cannot fully exclude the contribution of bystander activation. Among these expanded clones, CX3CR1 + CD8 + T cells differed significantly across severity (*p* = 0.0310) and were more abundant in DHF than in AD (Fig. 6b, left). Conversely, Int CD8 + T cells also showed significant differences (*p* = 0.0089) and were enriched in AD compared to the SD group (Fig. 6b, right), consistent with the trends previously observed in sorted GTS/NYA-specific CD8 + T cells (Fig. 3b). These results suggest that the observed disease severity-associated subset biases may reflect broader features of the CD8 + T cell response beyond the originally sorted epitope-specific populations.

Next, a longitudinal analysis of CD8 + T cell subsets was conducted based on the integrated GTS-specific CD8 + T cell data from paired donors during the acute (D-1) and two-month post-fever (F2) phases (Fig. 1a). While clonally expanded CX3CR1 + CD8 + T cells dominate in the acute phase in both DHF and DF, the majority of clonally expanded CD8 + T cells shift toward Int CD8 at F2 (Supplementary Fig. 12d). These findings highlight distinct temporal dynamics of CD8 + T cell subsets over the course of infection. To further characterize these patterns, we longitudinally tracked six GTS-specific CD8 + T cell clones from donor I085, the only donor with sampling across three timepoints: D-1, F1, and F2 timepoints. Consistent with the observations based on two timepoints (Fig. 5g), we observed dynamic clonal expansion at different phases of infection (Supplementary Fig. 12e). Collectively, this clonal-level analysis highlights a dynamic trajectory marked by robust activation during the acute phase, followed by the dominance of CX3CR1+ and Int CD8 + T cell subsets during convalescence (Supplementary Fig. 12f).

### Potential cell-cell interactions and plasma cytokine correlation of clonally expanded CD8 + T cell reveal distinct immune signatures in AD and DHF

We next investigated potential intercellular communication between the identified CD8 + T cell subsets and other PBMC populations using CellChatDB^41^. Building on our previous observation that CD8 + TEM cells in AD donors showed stronger predicted MHC class I-mediated interactions with memory B cells and CD16+ monocytes, which are potential viral reservoirs^18^, we extended the analysis to the newly defined CD8 + TEM subsets. Notably, Int CD8 + T cells showed more pronounced enrichment of predicted interactions with both memory B cells and CD16+ monocytes in AD, involving not only MHC class I-related interactions but also molecules associated with immunological synapses, including *ICAM1*-*ITGAL*/*ITGB2* (encoding LFA-1)^42^ and *CD99*^43,44^ (Fig. 6c). CX3CR1+ and CXCR6 + CD8 + T cells also exhibited positive communication probabilities with memory B cells and CD16+ monocytes, although the elevated levels of predicted interactions in AD over DHF were less pronounced (Fig. 6c). These results suggest possible differences in the modes of CD8 + T cell communication across clinical phenotypes. In AD, the higher levels of predicted interactions involving Int CD8 + T cells and potential viral reservoirs, particularly via molecules associated with immunological synapses, may be linked to a more regulated immune response that preserves viral control while limiting immunopathology. Conversely, the lower levels of such predicted interactions in DHF, including those involving CX3CR1 + CD8 + T cells, may reflect a more limited pattern of cell–cell communication, which may contribute to heightened inflammation and immunopathology.

To better understand the link between clonally expanded CD8 + T cell subsets and cytokine storm, known to correlate with severe diseases, we next examined pairwise Spearman’s rank correlations between plasma cytokine levels and the abundance of the clonally expanded CD8 + T cell subsets across donors during the acute phase (Supplementary Fig. 13a, b). The clonally expanded CX3CR1 + CD8 + T cells were positively correlated with several cytokines previously shown to correlate with disease severity, including MIP-1α, Eotaxin, IL-15, IL-1α, IL-2, G-CSF, IL-10 and IL-1β (Fig. 6d). In contrast, clonally-expanded Int CD8 + T cells were positively correlated with MDC and RANTES, but negatively correlated with IL-1α (Fig. 6e). Although causal relationships could not be directly determined, these findings suggest that clonally expanded CX3CR1 + CD8 + T cells, which may contribute to disease severity, are linked to several severity-associated cytokines previously described^30^. The positive association with IL-10 further supports a potential connection between CX3CR1 + CD8 + T cell–mediated pathology and the IL-10–driven plasmablast axis previously proposed in DHF^18^.

## Discussion

The role of DENV-specific CD8 + T cells in natural dengue infection remains insufficiently understood, particularly in the context of AD cases, which are rarely investigated in human studies. Here, we demonstrate the application of single-cell transcriptomic profiling in characterizing these cells beyond what is detectable by conventional pHLA-tetramer–based flow cytometry. This approach enabled high-resolution mapping of CD8 + T cell subsets bound to different DENV serotype NS3 epitopes across donors, clinical severities, and stages of infection, along with inference of their paired TCR sequences and relative avidity. During the acute phase, CX3CR1 + CD8 + T cells, exhibiting higher TCR avidity, a tendency of biased epitope binding toward serologically inferred dominant previously infecting serotypes, and elevated expression of genes associated with TCR signaling and cytotoxicity, were expanded in SD cases. In contrast, AD cases were enriched for the Int CD8 + T cell subset, characterized by relatively lower avidity in TCR binding, with a trend of preferential binding to peptides derived from the currently infecting serotype, and a transcriptional profile indicative of moderate cytotoxic potential. TCR repertoires based on paired V(D)J gene usage were largely distinct between severity groups, with some public features observed among SD donors. Longitudinal profiling across multiple timepoints revealed temporal shifts in both TCR composition and functional phenotype of DENV NS3-specific CD8 + T cell subsets, including contraction of early-expanded clones and emergence of distinct subsets during convalescence. We also extended these findings by integrating our dataset with previously published scRNA-seq and plasma cytokine profiles from partially matched donors^18,30^, enabling broader characterization of CD8 + T cell states in the total systemic immune landscape. Overall, our study provides insights into potential protective versus pathogenic roles of DENV NS3-specific CD8 + T cells, linking antigen specificity, epitope binding, phenotypic diversity, and systemic inflammatory signatures to distinct disease outcomes.

CX3CR1 has been broadly associated with terminally differentiated, inflammation-associated CD8 + T cell states, including TEM and TEMRA phenotypes^33,45,46^, and has been linked to vascular inflammation and tissue damage^47–50^. These cells interact with CX3CL1-expressing endothelial cells, facilitating trafficking and initiating downstream signaling pathways that enhance cytotoxicity and pro-inflammatory cytokine production, possibly independent of TCR engagement^50–53^. While CX3CR1-expressing CD8 + T cells were often considered protective in viral infection^54^, their ability to home to the endothelium via the CX3CR1-CX3CL1 axis may contribute to vascular leakage in dengue via released cytokines and cytotoxic molecules^55^. In the context of DHF, clonally expanded CX3CR1 + CD8 + T cells were enriched and correlated with elevated levels of inflammatory cytokines and chemokines such as IL-15, MIP-1α, Eotaxin, and IL-10, which have previously been linked to severe disease^30^. IL-15, in particular, was shown to drive activation and cytotoxicity of CX3CR1 + CD8 + T cells^50,56^. Once at the endothelial interface, CX3CR1 + CD8 + T cells may mediate damage through the release of cytotoxic molecules and inflammatory cytokines. Additionally, these cells upregulate genes including *CCL3* and *CCL4* (encoding MIP-1α and MIP-1β, respectively), which may further promote the recruitment of innate immune cells^35^, leading to amplified inflammation and endothelial activation. Collectively, these findings support a model in which DENV NS3-specific CX3CR1 + CD8 + T cells, through their cytotoxicity, cytokine production, and innate cell recruitment, contribute to tissue inflammation, endothelial injury, and vascular leakage. This is consistent with their enrichment in individuals with severe dengue (Supplementary Fig. 14). However, these observations are based on cross-sectional associations, and further mechanistic studies will be required to determine whether these cellular states play a causal role in dengue disease progression. Further in vitro and in vivo studies are needed to validate the role of CX3CR1 and assess whether the predisposition of CX3CR1 + CD8 + T cells during convalescence influences responses to subsequent infections.

Notably, CX3CR1 + CD8 + T cells also exhibited higher TCR avidity, a feature associated with enhanced polyfunctionality in antigen-specific responses^15,26^. Although the expression of early TCR response genes, such as those in the NR4A family, was low, these cells upregulated downstream effector genes, including *NFATC2* (NFAT1) and *TOX*, implying recent or sustained TCR signaling, potentially due to prior strong TCR activation. The co-expression of these molecules may reflect a poised or persistent effector state^38,57^, supporting the idea that CX3CR1 + CD8 + T cells contribute to the immunological imprinting and effector potential that influence clinical outcomes during acute secondary heterotypic infection.

Cross-reactive DENV-specific memory CD8 + T cells have been associated with functional impairment, immunopathology, and protection in dengue infection^10,14,15^, but these insights were largely derived from convalescent-phase samples. In our study, we found that during the acute phase, DENV NS3-specific CX3CR1 + CD8 + T cells, enriched in DHF, tend to bind to peptides derived from serologically inferred dominant previously infecting DENV serotype, while Int CD8 + T cells, enriched in AD, had a trend to bind to peptides derived from the currently infecting ones. These findings suggest that early responses may be primarily driven by mono-reactive DENV NS3-specific CD8 + T cells, either to the current or prior serotypes, and the double-positive ones may expand later in infection, consistent with our longitudinal profiling, which showed dynamic shifts in TCR usage and subset composition. As these cell states align with clinical presentation, phenotypes captured during acute infection may provide more direct insights into mechanisms underlying divergent disease outcomes. These results also highlight the importance of temporally resolved sampling to study the role of CD8 + T cells in dengue pathogenesis.

In AD, we observed an increase in DENV NS3-specific Int CD8 + T cells with intermediate expression of cytotoxicity-related genes, low TCR avidity, and predominant binding to peptides derived from the current infecting serotypes. The moderate cytotoxic profile and the enrichment of TGF-β and TNF-induced NF-κB signaling pathways, both previously shown to compromise CD8 + T cell effector functions and enhance apoptosis^58–60^, may help limit excessive immune activation and reduce the risk of severe disease^20^. Despite their low avidity, DENV NS3-specific Int CD8 + T cells were predicted to maintain relatively stronger contact with potential viral reservoirs and exhibit upregulated genes associated with TCR signaling (Supplementary Fig. 14). Additionally, proportions of this subset were negatively correlated with proinflammatory cytokines but positively correlated with MDC and RANTES, further supporting controlled levels of inflammation in AD. These features suggest that DENV NS3-specific Int CD8 + T cells may support an optimally regulated immune response, where moderate cytotoxicity combined with sustained antigen presenting cell  engagement contributes to viral clearance, even at a slower rate^19^, without contributing to excessive tissue damage. Nevertheless, because the precise infection stage in AD individuals cannot be definitively determined, the potential influence of infection timing on these observations cannot be fully excluded.

For TCR analysis, we observed preferential usage of TRBV genes such as *TRBV11-2*, *TRBV9*, and *TRBV27* in GTS-HLA-A*11-reactive CD8 + T cells, consistent with prior reports linking these genes to the Asn58β residue in the CDR2β loop, which is critical for GTS binding across DENV variants^26^. Interestingly, we also found frequent usage of other TRBV genes lacking the Asn58β residue, suggesting alternative binding mechanisms or contributions from other TCR regions^61^. Moreover, the TCR repertoire of GTS-specific CD8 + T cells varied across disease severities, with certain GTS-specific CD8 + T cells in DF and DHF sharing paired V(D)J gene usage and conserved CDR3 motifs, indicating the presence of public TCRs in SD. This pattern is similar to observation in SARS-CoV-2 infection, supporting a link between TCR diversity, immune response strength, and disease outcome^62–64^. Together, these findings suggest that distinct V(D)J gene usage and CDR3 features may underlie differential CD8 + T cell responses associated with disease severities.

Despite these important findings, our study has limitations as discussed here. The relatively small cohort size and number of tetramer-positive cells in certain donors, together with the restricted range of HLAs and epitopes analyzed, may restrict the generalizability of our results. Further studies with broader pHLA and a larger sample size, and more efficient antigen-specific T-cell capture techniques are warranted. For some samples, particularly at the acute phase, the number of tetramers captured, FACS-sorted DENV NS3-specific CD8 + T cells was relatively low, likely due to the known phenomenon of TCR downregulation after activation^39^. We attempted to address this issue by integrating the data with total CD8 + T cells from the partially matched droplet-based dataset, where we identified a higher number of cells within the same clonotype networks as defined by shared V(D)J gene usage and CDR3 sequences, enabling us to better characterize the heterogeneity of DENV NS3-specific CD8 + T cells and confirm what we found based on the sorted T cell data. Where applicable, quantitative comparisons were performed at the donor level to account for differences in recovered cell numbers across individuals.

Another limitation relates to the determination of infection history. Primary versus secondary infection status was determined using antibody-based assays such as PRNT or hemagglutination inhibition (HI) assay^65^, which may not perfectly reconstruct historical infecting serotypes, particularly in endemic settings. To reflect this limitation, the relevant serotypes assigned to these samples are described as serologically inferred dominant previously infecting serotypes, defined operationally based on neutralization titers rather than definitive virological confirmation historically. Consistent with this limitation, we identified one AD case initially classified as primary based on serology that may instead represent a secondary infection, as suggested by detectable DENV NS3-specific T-cell responses to inferred previously encountered serotypes. This observation highlights a potential limitation of antibody-based determination of previously infecting serotypes, particularly in AD, which is characterized by substantial T cell responses and relatively lower antibody ones^18^. In addition, serotype-specific neutralizing antibody and CD8 + T-cell responses do not necessarily correlate directly (Supplementary Tables 8 and 9) and may reflect differential or biased immune recall within distinct immune compartments. We suggest that, in such cases, investigating CD8 + T cell binding to different DENV serotypes may provide a useful complementary approach to more accurately determine infection history.

In summary, our findings underscore the phenotypic and functional heterogeneity of DENV NS3-specific CD8 + T cells and their context-dependent roles in both protective immunity and immunopathogenesis. Disease severity may not be solely dictated by T cell reactivity but by the qualitative features of specific subsets. High-avidity, clonally expanded CX3CR1 + CD8 + T cells, likely imprinted from prior infection, exhibit potentially potent effector functions that may contribute to vascular pathology in severe dengue. Conversely, currently activated Int CD8 + T cells, characterized by restrained expression of genes related to cytotoxicity and immunoregulatory signatures, are enriched in AD and may support effective viral control with limited immunopathology. These distinctions also align with the divergence in TCR repertoires between AD and clinical dengue, potentially underlying such distinct DENV NS3-specific CD8 + T cell responses. Together, these insights may inform better rational vaccine designs aimed at eliciting protective CD8 + T cell responses and therapeutic strategies to mitigate disease severity.

## Methods

### Ethics statement and study cohort

As part of the dengue research framework for resisting epidemics in Europe (DENFREE) initiative, the DENFREE Thailand cohort was established. DENV-infected patients and their household members (HHMs) were recruited from three locations: Vajira Hospital (Bangkok, Thailand), the Faculty of Tropical Medicine, Mahidol University (Bangkok, Thailand), and Tasongyang Hospital (Tak, Thailand). The study received approval from the Institutional Review Board (IRB) of the Faculty of Medicine, Vajira Hospital (No.015/12) and the Faculty of Tropical Medicine, Mahidol University (TMEC 13-041). Informed consent was obtained from all participants. The use of archived samples in this study was approved by the IRB of the Faculty of Medicine, Ramathibodi Hospital, Mahidol University (MURA2016/219 and MURA2019/603), and the UK North West York Ethics Committee (19/NE/0170).

### Study population and sample collection

Recruitment of SD index cases and AD cases has been previously described^19^. Briefly, SD cases were enrolled based on the presentation of high fever (≥38 °C) and at least two symptoms of dengue disease, such as severe headache, retro-orbital pain, muscle pain, joint pain, skin rash, or hemorrhagic manifestations, coupled with the detection of DENV RNA via qRT-PCR or anti-DENV IgM or NS1 antigen. Systematic surveillance of HHMs of SD index cases identified AD individuals as those exhibiting DENV viremia (determined by qRT-PCR) but showing no clinical symptoms during a 2-week follow-up. For AD subjects, PBMC and plasma samples were collected upon enrollment. PBMCs were isolated using a density gradient method and stored in liquid nitrogen for subsequent experiments.

### Clinical samples

To standardize collection time points during acute DENV infection and minimize confounding variables, SD samples were collected on the first day of enrollment during the acute febrile phase (24–48 h before defervescence). The parental cohort for total immune profiling consisted of 24 samples, including 8 AD, 8 DF, and 8 DHF^18^. HLA typing was performed using sequence-specific primer PCR^66^. For comparative analysis of DENV-specific CD8 + T cell analysis, we selected samples from the parental cohort that were HLA-A*11-positive or HLA-A*24 -positive , comprising 4 AD, 7 DF, and 5 DHF, enabling integrated analysis across different immune profiling aspects. Sample selection ensured balance in confounding variables such as viremia levels, age and gender (Supplementary Fig. 1a-d). For the longitudinal cohort, paired samples from 3 DF and 3 DHF patients were collected during the febrile phase and at a 2-month follow-up to monitor immune response dynamics. Currently infecting serotype with level of DENV viremia was quantified via qRT-PCR, while primary versus secondary infections in SD cases were defined by a dengue-specific IgM/IgG ratio of less than 1.8, determined by IgM and IgG capture ELISA, or by a ≥ 4-fold rise in hemagglutination inhibition (HI) antibody titers against any dengue serotype between paired acute and convalescent samples. For AD cases lacking paired samples, neutralizing antibody responses were assessed using plaque reduction neutralization tests (PRNT)^67^, distinguishing primary from secondary infections based on antibody specificity and titers. Participant and clinical sample details are listed (Supplementary Tables 1 and 2).

### Identification of DENV NS3-specific CD8 + T cells

DENV NS3-specific CD8 + T cells were identified using HLA-A*11 and HLA-A*24 tetramers loaded with DENV peptides NS3_133-142_, NS_556-564_ specific for DENV serotypes, labeled with either PE or APC. Monomers were generated using previously validated immunodominant NS3 epitopes reported in prior studies^13,14^. The amino acid sequences of all peptide variants used for tetramer generation are provided in Supplementary Table 3. PBMCs were incubated with the LIVE/DEAD Fixable Green Dead Cell Stain Kit at room temperature (RT). Tetramers targeting the currently infecting serotype and selected previously infecting serotypes, as defined by PRNT or HI, were used to stain the PBMCs. We selected serologically inferred dominant previously infecting serotypes as the non-current DENV serotype with the highest PRNT titer in the acute phase. Tetramer staining was performed at saturating concentrations (1 µg monomer each) to ensure maximal occupancy of available TCR binding sites. After a 30-min incubation at 37 °C, cells were stained with an antibody cocktail containing 6 antibodies, including anti-CD3 PerCP, anti-CD8 APC-H7, anti-CD4 FITC, anti-CD14 FITC, anti-CD19 FITC, and anti-CD56 FITC (BD Biosciences, USA), on ice for 30 min in the dark. Lymphocytes were pre-gated based on FSC-A and SSC-A, followed by singlet gating using FSC-A and FSC-H. Non-relevant cells, including monocytes, CD4 + T cells, B cells, NK cells, and dead cells (FITC-positive), were excluded from the singlet population. CD8 + T cells were identified by co-expression of CD3 and CD8, while tetramer-positive (Tet + ) CD8 + T cells were identified by the presence of PE and APC signals. Using the FACSAria III cell sorter (BD Biosciences, USA), Tet+ CD8 + T cells were single-cell sorted into 96-well plates containing lysis buffer, snap-frozen, and stored at −80 °C until further use. FACS data, including phenotype and fluorescence intensity, were recorded for individual cells via index sorting. Data visualization and gating strategies were analyzed using FlowJo™ v10.10 Software (Supplementary Fig. 2a). The number of sorted cells for each donor is shown in Supplementary Table 4.

### Library preparation, sequencing, and alignment

For full-length transcriptome analysis, the Smart-seq2 (SS2) protocol^68^ was performed with the following modifications, including increasing preamplification PCR cycles to 23 to obtain sufficient cDNA for downstream analysis. PCR purification of whole transcriptome amplification (WTA) was automated using a Zephyr machine. cDNA ranging from 400–5000 bp was quantified by the 4150 TapeStation system and normalized across wells. Fragmented cDNA (500 pg/µl) was processed using the Nextera XT library preparation kit (Illumina), incorporating library index sets, and amplified over 11 PCR cycles. Libraries were pooled in four unique index sets. The libraries underwent paired-end sequencing on the Illumina HiSeq4000 platform (Wellcome-Sanger Institute, UK). Raw FASTQ data were aligned to the GRCh38 reference genome using Bowtie within the TraCeR pipeline^69^.

### scRNA seq data analysis

Single-cell RNA sequencing (scRNA-seq) data were analyzed using the Scanpy package (v1.8.2). After importing and concatenating datasets, cells expressing fewer than 200 genes or genes detected in fewer than three cells were excluded. Additional quality control filters were applied to remove cells with mitochondrial gene content exceeding 17.5%, gene counts below 5000, or total reads above 4 million to ensure high-quality data and minimize inclusion of droplets. The number of cells passing quality control for each donor is shown in Supplementary Table 5. The data were normalized to 10⁴ counts per cell and log-transformed. To correct for batch effects, likely introduced during PCR amplification, plate-specific identifiers were used. Highly variable genes were selected while excluding TCR/BCR genes to prevent bias, and batch correction was performed using the Scanorama extension (sigma = 3). UMAP visualization was generated with n_neighbors set to 13 and n_pcs to 6, and unbiased clustering was performed at a resolution of 0.32. CD8 + T cell subsets were annotated through a combination of reference-based CellTypist prediction and manual curation based on well-established markers.

Differential gene expression (DGE) analysis was conducted using the Wilcoxon method implemented in Scanpy, filtering out genes with adjusted *p*-values greater than 0.05 or log₂-fold changes less than 1.5. Over-representation analysis (ORA) was then performed using the GSEApy package with gene sets from the MSigDB 2020 and Elsevier pathway collections to identify pathways enriched within clusters. CD8 + T cell differentiation trajectories were reconstructed using the partition-based graph abstraction (PAGA) method in Scanpy to map connectivity between clusters, with Naive/CM CD8 designated as the root, and diffusion pseudotime (DPT) calculated with n_dcs set to 10 to track temporal gene expression dynamics.

For cell-cell communication analysis, CellChat objects generated from 10X scRNA-seq data of AD and DHF samples were reannotated using inferred GTS-specific CD8+ T-cell subset identities. Cell-cell communication networks were inferred using CellChatDB version 1.6.1^41^ based on the overexpression of literature-curated receptor-ligand pairs, excluding pathways involving fewer than 10 cells. Communication probabilities were computed at the signaling pathway level, and aggregated networks were analyzed to identify significant intercellular interactions.

### TCR analysis

TCR analysis was performed by reconstructing TCR sequences using TraCeR. FASTQ files were aligned to reference sequences, specifically targeting VJ recombinations for alpha chains and V(D)J recombinations for beta chains, using Bowtie, which supports spliced alignments including joining regions. Trinity software was then used to assemble contigs, followed by TCR gene segment annotation using IgBlast, and transcript abundance quantification using Kallisto. Single-cell V(D)J sequencing analysis and visualization were carried out using the Scirpy package (v0.10.1). Productive TCR chain pairs of GTS-specific CD8 + T cells were identified in 875 cells, with the number of cells per donor shown in Supplementary Table 6. Clonotypes were defined based on shared TCR gene usage and CDR3 nucleotide sequences for both the alpha and beta chains. Clonotype networks were constructed based on amino acid sequence similarity using an alignment matrix, with a cutoff of 15. For integrated analysis across different chemistries, the Levenshtein metric was applied with a cutoff distance of 2, and clusters were required to have matching VJ and V(D)J receptor arms for inclusion. Motif analysis was performed using the Scirpy package (v0.13.0).

### Dataset integration

All inferred GTS-specific CD8 T cells from SS2 and 10X datasets were log-normalized, and the top 2000 variable features were selected using the Seurat package (v4.3.0) to replicate and validate the phenotype of sorted DENV NS3-specific CD8 + T cells observed in the SS2 data. Integration was performed using Canonical Correlation Analysis (CCA), which involved identifying shared features, scaling the data, and conducting principal component analysis (PCA). Integration anchors were identified across datasets to generate batch-corrected data, referred to as the GTS dataset. Clustering at a resolution of 0.7 identified five distinct clusters.

### Multimodal reference mapping

Query mapping to the reference dataset was performed using the Seurat package (v4.3.0). The UMAP from the integrated GTS-specific CD8+ T cell data served as the reference. Raw concatenated 10X GEX data were normalized, and anchors were identified using the FindTransferAnchors method (k.anchor  = 6, dims = 1:30, reference.reduction = “pca”). Mapped data were projected onto the reference space to identify CD8 + T cell subsets for integration into the query datasets (k.weight = 40, dims = 1:30). After identifying anchors for each sample, cell-type labels from the reference were transferred to the corresponding query dataset.

### Flow cytometry analysis

Frozen PBMC samples from 11 DF and 9 DHF HLA-A*11 patients (see Supplementary Table 7) were thawed and incubated with 100 nM dasatinib (Sigma-Aldrich, USA), for 30 min to enhance the detection of Tet+ CD8 + T cells. HLA-A*11 tetramers were used to identify CD8 + T cells specific to the currently infecting serotype (PE-tagged) and serotypes 1 and 2 (APC-tagged). Fc receptors were blocked using TruStain FCX (BioLegend, USA), and cells were stained with an antibody cocktail containing 7 fluorochrome-conjugated antibodies, including anti-CD3 PerCP, anti-CD8 APC-H7, anti-CD45RA BV510, anti-CD45RO BV421, anti-CCR7 BV786 (BD Biosciences, USA), anti-CX3CR1 PE-Cy7, and anti-CXCR6 FITC (BioLegend, USA), on ice for 30 min. Stained cells were acquired using the FACSMelody flow cytometer (BD Biosciences, USA). Lymphocytes were pre-gated based on FSC-A and SSC-A, followed by gating singlets using FSC-H and FSC-A. CD8 + T cells were identified by co-expression of CD3 and CD8. Tet+ CD8 + T cells were subsequently identified by the presence of either PE or APC signals, above the levels observed in non-tetramer-stained controls. Tet+ CD8 + T cell subsets were determined based on the expression of CD45RA, CD45RO, and CCR7 markers: Naive (CD45RA + CD45RO − CCR7 + ), TCM (CD45RA − CD45RO + CCR7 + ), TEM (CD45RA − CD45RO + CCR7 − ), and TEMRA (CD45RA + CD45RO − CCR7 − ). Fluorescence minus one (FMO) control for each antibody was used for gating, and the gating strategy is shown in Supplementary Fig. 5b. To assess tetramer specificity across the four DENV serotypes, PBMC samples collected at defervescence (D0) were first stained with LIVE/DEAD Fixable Green Dead Cell Stain and treated with dasatinib prior to tetramer staining. Cells were then incubated with serotype-specific DENV NS3 peptide–HLA tetramers labeled with PE, APC, BV421, and PE-Cy7. Tet+ CD8 + T cells were identified based on tetramer fluorescence above FMO controls. CD8 + T cells binding to different DENV serotype NS3 epitopes and double-positive CD8 + T cells were determined using Boolean gating in FlowJo™ v10.10 software (see Supplementary Tables 8 and 9).

### Statistical analysis

Statistical analyses were performed using GraphPad Prism version 8.0 (GraphPad Software, Inc., La Jolla, CA, USA; https://www.graphpad.com/). Comparisons across severity groups were conducted using non-parametric, two-tailed tests. Multi-group comparisons were performed with the Kruskal–Wallis test, followed by post-hoc Dunn’s test with the two-stage linear step-up procedure of Benjamini, Krieger, and Yekutieli (BKY) to control the false discovery rate. Pairwise analyses were performed using the two-tailed Mann–Whitney *U*-test. Associations between sex and clinical groups were tested using the Chi-square test. Correlations between clonally expanded subsets and plasma cytokine levels were assessed using Spearman’s rank correlation coefficient, implemented in Python with the scipy.stats package. The composite tetramer fluorescence index across CD8 + T-cell clusters was evaluated using a linear mixed-effects regression model with the cell cluster as a fixed effect and donor as a random intercept, implemented in the statsmodels package (model described in Supplementary Methods). Associations between TCR VJ cluster distribution and disease severity were assessed using the Fisher-Freeman-Halton exact test with Monte Carlo simulation (scipy.stats). Global differences in TCR repertoire composition were evaluated using PCA of donor-level V(D)J gene usage, and the association between cluster membership and disease severity was evaluated using the Fisher–Freeman–Halton exact test. A *p*-value less than 0.05 was considered statistically significant.

### Reporting summary

Further information on research design is available in the Nature Portfolio Reporting Summary linked to this article.

## Supplementary information

Supplementary Information

Peer Review file

Reporting Summary

## Source data

Source Data 1

Source Data 2

## Data Availability

The raw single-cell RNA sequencing and TCR sequencing data generated in this study have been deposited in the European Genome-Phenome Archive (EGA) under accession code EGAD00001015637. The data are available under controlled access due to ethical and privacy considerations for human participants, and can be obtained by application to the relevant data access committee through the EGA repository. Raw sequencing data are protected and not publicly available due to data privacy regulations. Processed data supporting the findings of this study are provided in the Supplementary Information and Source Data files. Source data are provided with this paper.

## Code availability

All data analysis scripts are available at GitHub: https://github.com/sirawitsrikor/dengue-gex-tcr-analysis.
