# Supplementary material for: Heterogeneity and dynamics of DENV-specific CD8 + T cells in dengue infection
Source: Nat Commun. 2026 Jun 3;17:7103. doi: 10.1038/s41467-026-73491-5 (PMC13392236; doi:10.1038/s41467-026-73491-5)
Supplement: Supplementary file 1 — Supplementary Information [file 41467_2026_73491_MOESM1_ESM.pdf]

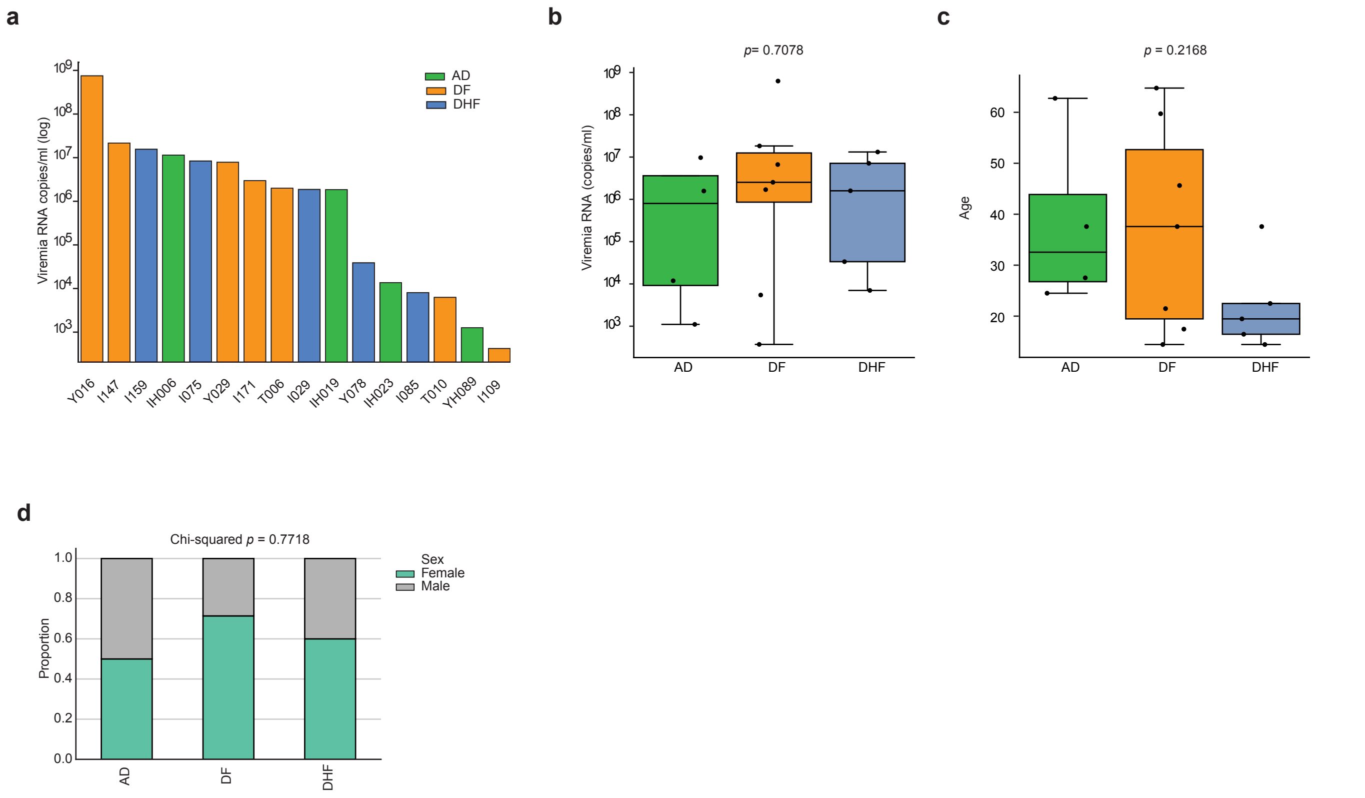

Supplementary Figure 1. Demographic and clinical characteristics of the cohort used for SS2 scRNA-seq analysis. (a) Viremia levels (RNA copies/ml, $\log_{10}$ scale) across individual donors, stratified by disease severity. (b) Comparison of viremia levels across severity groups (AD, DF, DHF). Box plots show median and interquartile range (IQR), with whiskers indicating 1.5× IQR; points represent individual donors. Statistical analysis was performed using the Kruskal–Wallis test (p = 0.7078). (c) Age distribution across severity groups. Statistical analysis was performed using the Kruskal–Wallis test (p = 0.2168). (d) Sex distribution across severity groups. Statistical analysis was performed using the chi-squared test (p = 0.7718)

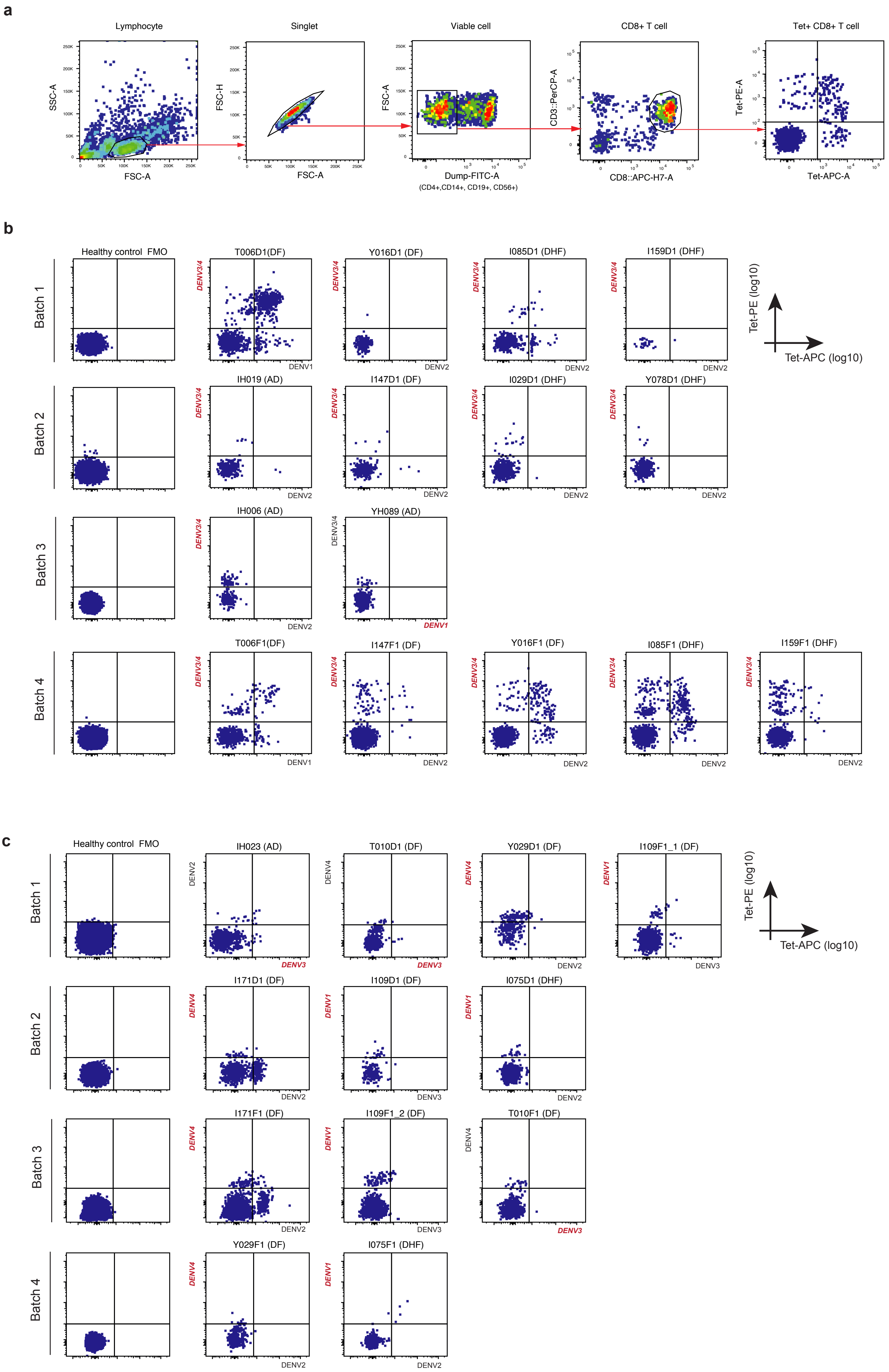
a
Lymphocyte
SSC-A
FSC-A
Singlet
FSC-H
FSC-A
Viable cell
FSC-A
Dump-FITC-A
(CD4+,CD14+, CD19+, CD56+)
CD8+ T cell
CD3::PerCP-A
CD8::APC-H7-A
Tet+ CD8+ T cell
Tet-PE-A
Tet-APC-A
b
Batch 1
Healthy control FMO
T006D1(DF)
DENV3/4
DENV1
Y016D1 (DF)
DENV3/4
DENV2
I085D1 (DHF)
DENV3/4
DENV2
I159D1 (DHF)
DENV3/4
DENV2
Tet-PE (log10)
Tet-APC (log10)
Batch 2
IH019 (AD)
DENV3/4
DENV2
I147D1 (DF)
DENV3/4
DENV2
I029D1 (DHF)
DENV3/4
DENV2
Y078D1 (DHF)
DENV3/4
DENV2
Batch 3
IH006 (AD)
DENV3/4
DENV2
YH089 (AD)
DENV3/4
DENV1
Batch 4
T006F1(DF)
DENV3/4
DENV1
I147F1 (DF)
DENV3/4
DENV2
Y016F1 (DF)
DENV3/4
DENV2
I085F1 (DHF)
DENV3/4
DENV2
I159F1 (DHF)
DENV3/4
DENV2
c
Batch 1
Healthy control FMO
IH023 (AD)
DENV2
DENV3
T010D1 (DF)
DENV4
DENV3
Y029D1 (DF)
DENV4
DENV2
I109F1_1 (DF)
DENV1
DENV3
Tet-PE (log10)
Tet-APC (log10)
Batch 2
I171D1 (DF)
DENV4
DENV2
I109D1 (DF)
DENV1
DENV3
I075D1 (DHF)
DENV1
DENV2
Batch 3
I171F1 (DF)
DENV4
DENV2
I109F1_2 (DF)
DENV1
DENV3
T010F1 (DF)
DENV4
DENV3
Batch 4
Y029F1 (DF)
DENV4
DENV2
I075F1 (DHF)
DENV1
DENV2

Supplementary Figure 2. Flow cytometry gating strategy and tetramer staining plots for DENV-specific CD8+ T cells. (a) Gating strategy for identification of viable tetramer-positive CD8+ T cells. Lymphocytes were first gated on FSC-A vs. SSC-A, followed by singlet discrimination (FSC-A vs. FSC-H), and exclusion of dead cells and non-T cell populations (CD4+, CD19+, CD16+, CD56+) using a viability dye and lineage markers. CD3+CD8+ T cells were then gated, and DENV-specific CD8+ T cells were identified using tetramers. Each sample was stained with two tetramers: one specific for the serologically inferred dominant previously infecting serotype and one for the current infecting serotype, each conjugated to a different fluorochrome. (b–c) Representative tetramer staining plots of DENV-specific CD8+ T cells. Presorted CD8+ Tcells (double negative for PE and APC) are overlaid with sorted tetramer-positive CD8+ T cells from each sample. (b) GTS-specific CD8+ T cells in HLA-A*11+ donors. (c) NYA-specific CD8+ T cells in HLA-A*24+ donors across AD, DF, and DHF cases. Red denotes the currently infecting serotype, and black denotes the serologically inferred dominant previously infecting serotype (see Supplementary Tables 1–2). Numbers of sorted cells and epitope reactivities are provided in Supplementary Table 4. Fluorescence-minus-one (FMO) controls from healthy donors were included in the first column of each batch.

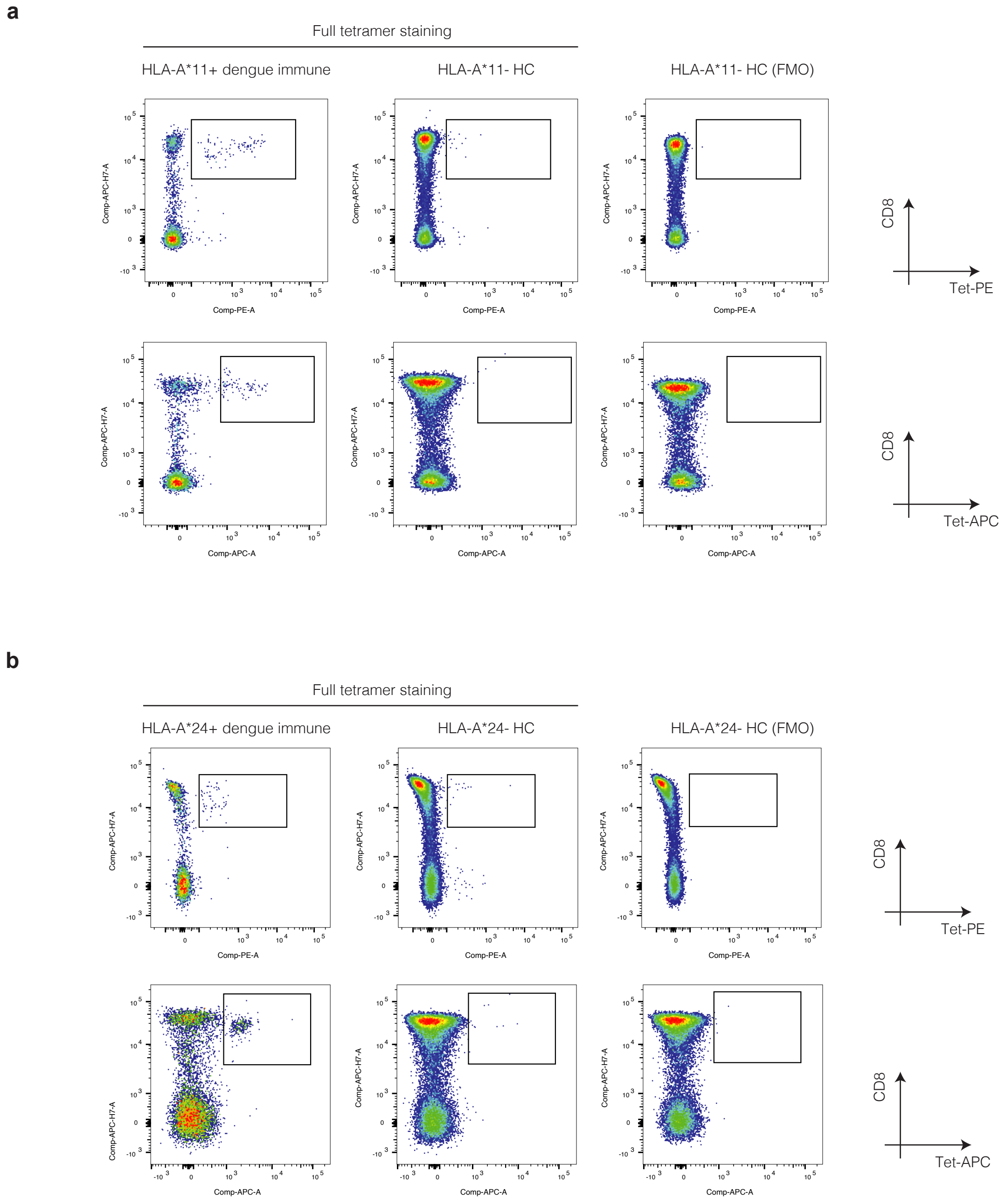

Supplementary Figure 3. Representative flow cytometry plots of Tet-PE (top) and Tet-APC (bottom) staining using GTS–HLA-A*11 tetramers (A) and NYA–HLA-A*24 tetramers (B). Lymphocytes were first gated based on FSC-A versus SSC-A, followed by singlet discrimination (FSC-A vs. FSC-H) and exclusion of dead cells and non–T cell populations (CD4+, CD19+, CD16+, CD56+) using a viability dye and lineage markers. Tetramer-positive CD8+ T cells were then gated, and boxed regions indicate tetramer-positive CD8+ T cells. Left: A dengue-immune donor shows a distinct tetramer-positive CD8+ T cell population (presorted cells overlaid with sorted CD8+ T cells). Middle: HLA-matched healthy controls show minimal background events. Right: Fluorescence-minus-one (FMO) controls overlap with the tetramer-negative population observed in fully stained samples. Gating thresholds were therefore defined based on the FMO controls.

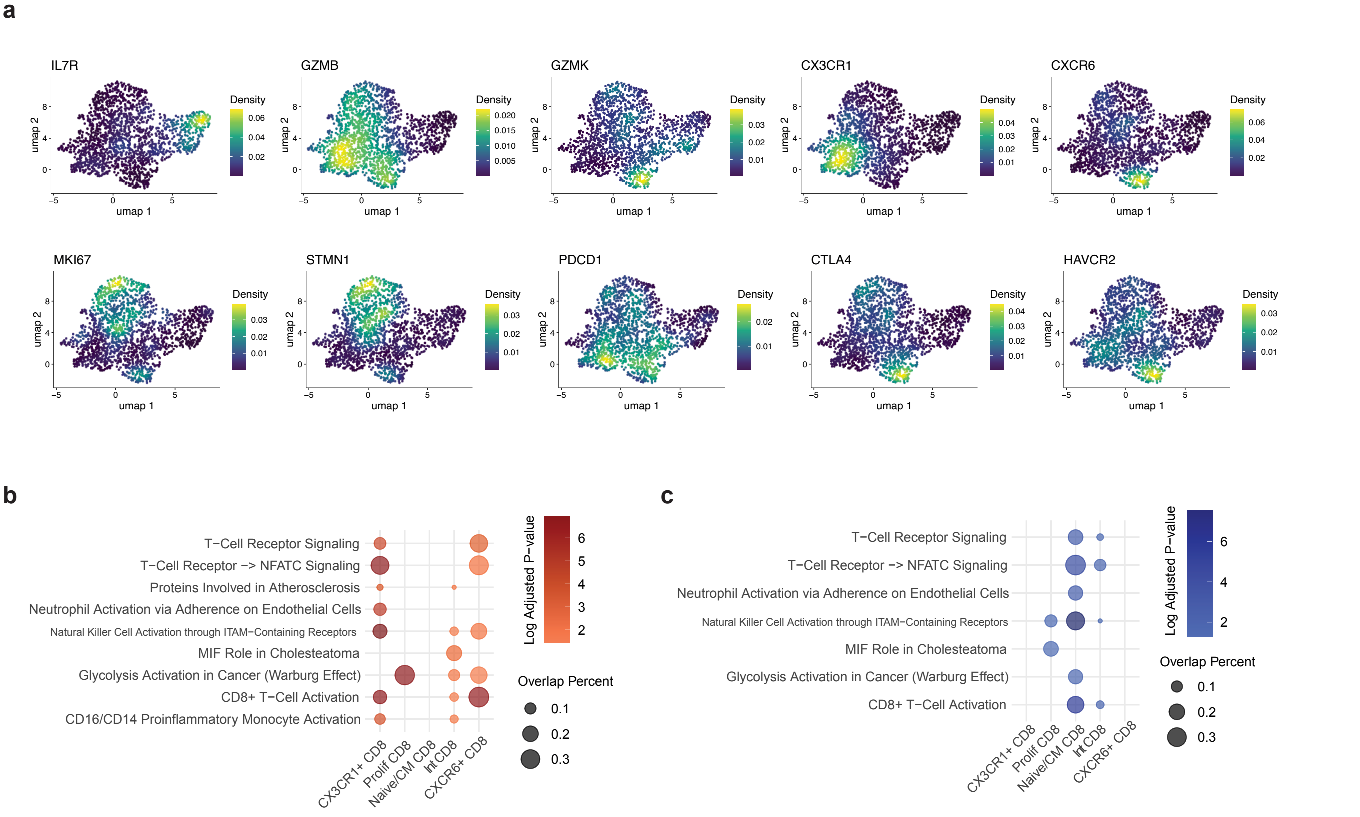

Supplementary Figure 4. Expression density and pathway enrichment analysis of DENV-specific CD8+ T cell subsets. (a) UMAP plots showing the expression density of selected genes related to T cell activation and cytotoxicity (*IL7R, GZMB, GZMK*), tissue homing (*CX3CR1, CXCR6*), proliferation (*MKI67, STMN1*), and inhibitory/exhaustion markers (*PDCD1, CTLA4, HAVCR2*) across DENV-specific CD8+ T cells. (b–c) Pathway enrichment analysis (ORA) of genes differentially expressed in each CD8+ T cell subset. Panel b shows enriched pathways among upregulated genes, and panel c shows those among downregulated genes. Dot size represents the percentage of overlapping genes, while color reflects pathway significance ($-\log_{10}$ adjusted p-value).

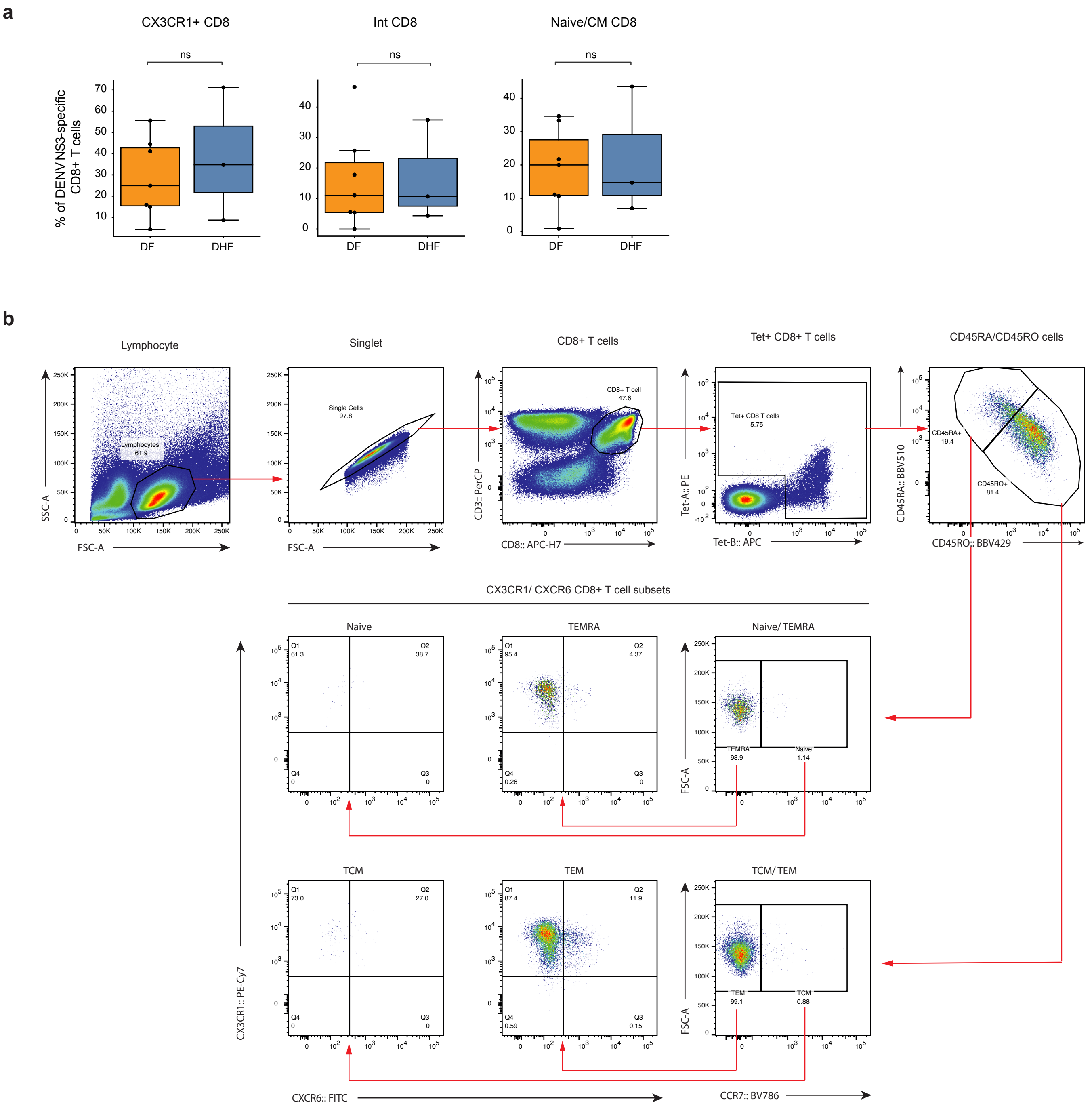

Supplementary Figure 5. Quantification of DENV-NS3 specific CD8+ T cell subsets during the convalescent phase and gating strategy for GTS-specific CD8+ T cell subsets in the validation cohort. (a) Quantification of CX3CR1+ CD8, Naive/CM CD8, and Int CD8 T cells among tetramer-positive DENV-NS3 specific CD8+ T cells, stratified by disease severity (DF, n = 7; DHF, n = 3) during the convalescent phase. Box plots show median and interquartile range (IQR); points represent individual donors. Statistical analysis was performed using the Mann–Whitney U test. No statistically significant differences were detected between groups. (b) Flow cytometry gating strategy for defining GTS-specific CD8+ T cell subsets from validation cohort. Lymphocytes were first gated, followed by singlet selection (FSC-A vs. FSC-H). CD3+CD8+ T cells were then identified. Tetramer-positive CD8+ T cells were further subdivided into Naive, TCM, TEM, and TEMRA subsets based on CD45RA, CD45RO, and CCR7 expression. Final gating using CX3CR1 and CXCR6 delineated subset heterogeneity within each CD8+ T cell population. Source data is provided as a Source Data file.

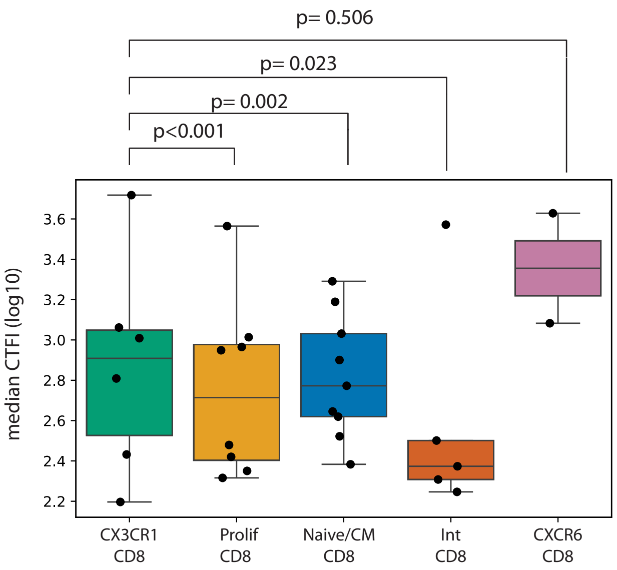

Supplementary Figure 6. Donor-level tetramer fluorescence intensity across CD8+ T-cell clusters during acute phase. Donor-level median composite tetramer fluorescence index (CTFI, log10) across CD8+ T-cell transcriptional clusters. CTFI was calculated by integrating tetramer PE-A and APC-A fluorescence signals as the Euclidean norm to capture overall tetramer staining intensity across channels. Each dot represents an individual donor, and box plots summarize the distribution of donor-level median CTFI values for each cluster (CX3CR1+ CD8, proliferating CD8, naïve/central memory CD8, intermediate CD8, and CXCR6+ CD8). Donor–cluster combinations represented by fewer than four cells were excluded. Statistical comparisons were performed using a linear mixed-effects regression model with cluster identity as a fixed effect and donor as a random intercept (shown in Supplementary method). Pairwise comparisons between clusters are indicated above the plot.

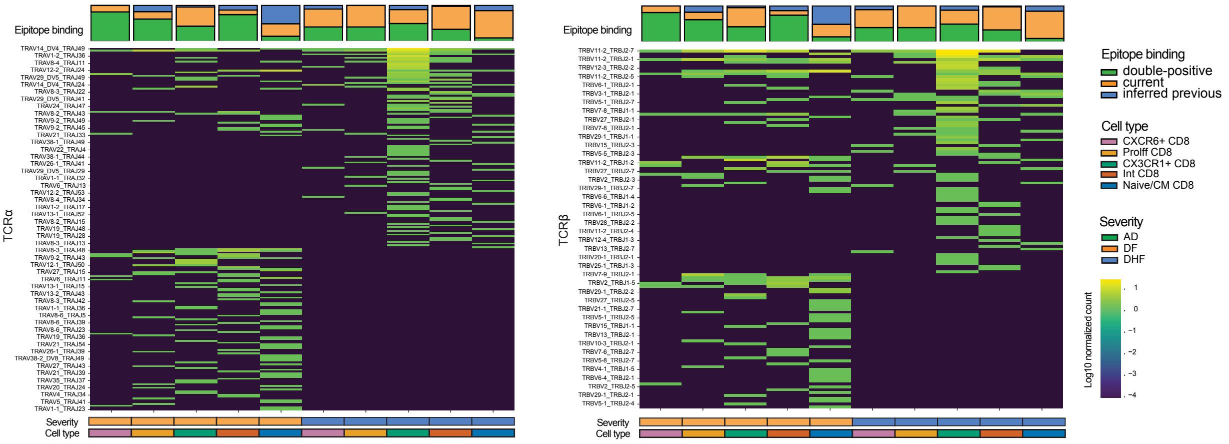

Supplementary Figure 7. TCRα and TCRβ V–J gene usage patterns across DENV-specific CD8+ T cell subsets during the convalescent phase. Heatmaps showing TRAV (left) and TRBV (right) V–J gene usage across tetramer-binding DENV-specific CD8+ T cells during the convalescent phase. TCRs are stratified by epitope binding (current-serotype, inferred previous-serotype, and double-positive), transcriptional subset identity, and disease severity (DF, DHF), as indicated by annotation bars. Each row represents a unique V–J gene pair, and each column corresponds to epitope reactivity for TCRα or TCRβ chains. Color intensity indicates normalized frequency of gene usage.

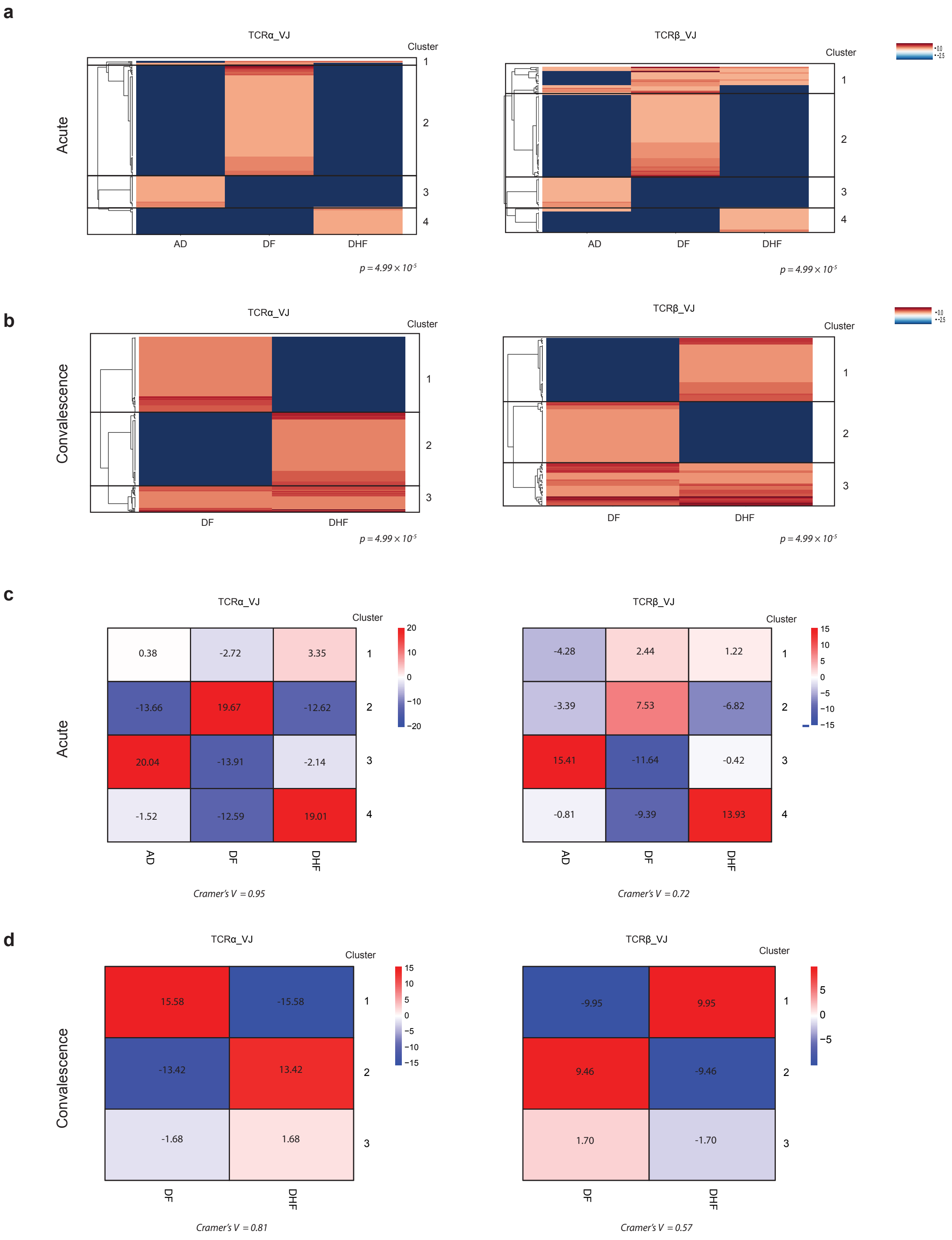

Supplementary Figure 8. Hierarchical clustering and standardized residual analysis of paired V–J gene usage in GTS-specific CD8+ T cells. Heatmaps show paired V–J gene usage for TCRα (left) and TCRβ (right). (a) Hierarchical clustering heatmaps of paired V–J gene usage across disease severity groups (AD, DF, DHF) during the acute phase. Distinct TCR clusters defined by major dendrogram branches show differential V–J usage patterns across severity groups (Fisher–Freeman–Halton exact test, $p = 4.99 \times 10^{-5}$). (b) Hierarchical clustering heatmaps comparing symptomatic dengue groups only (DF and DHF) during the convalescent phase, revealing three major TCR clusters with differential V–J gene usage (Fisher–Freeman–Halton exact test, $p = 4.99 \times 10^{-5}$). (c) Standardized residual heatmaps showing enrichment (red) or depletion (blue) of TCR clusters across severity groups (AD, DF, DHF) for TCRα_VJ (left) and TCRβ_VJ (right) during the acute phase. Strong associations were observed between cluster distribution and severity (Cramér's V: TCRα_VJ = 0.95; TCRβ_VJ = 0.72). (d) Standardized residual heatmaps comparing symptomatic groups (DF vs DHF) during the convalescent phase, highlighting cluster enrichment patterns. Strong associations were observed between cluster distribution and severity (Cramér's V: TCRα_VJ = 0.81; TCRβ_VJ = 0.57).

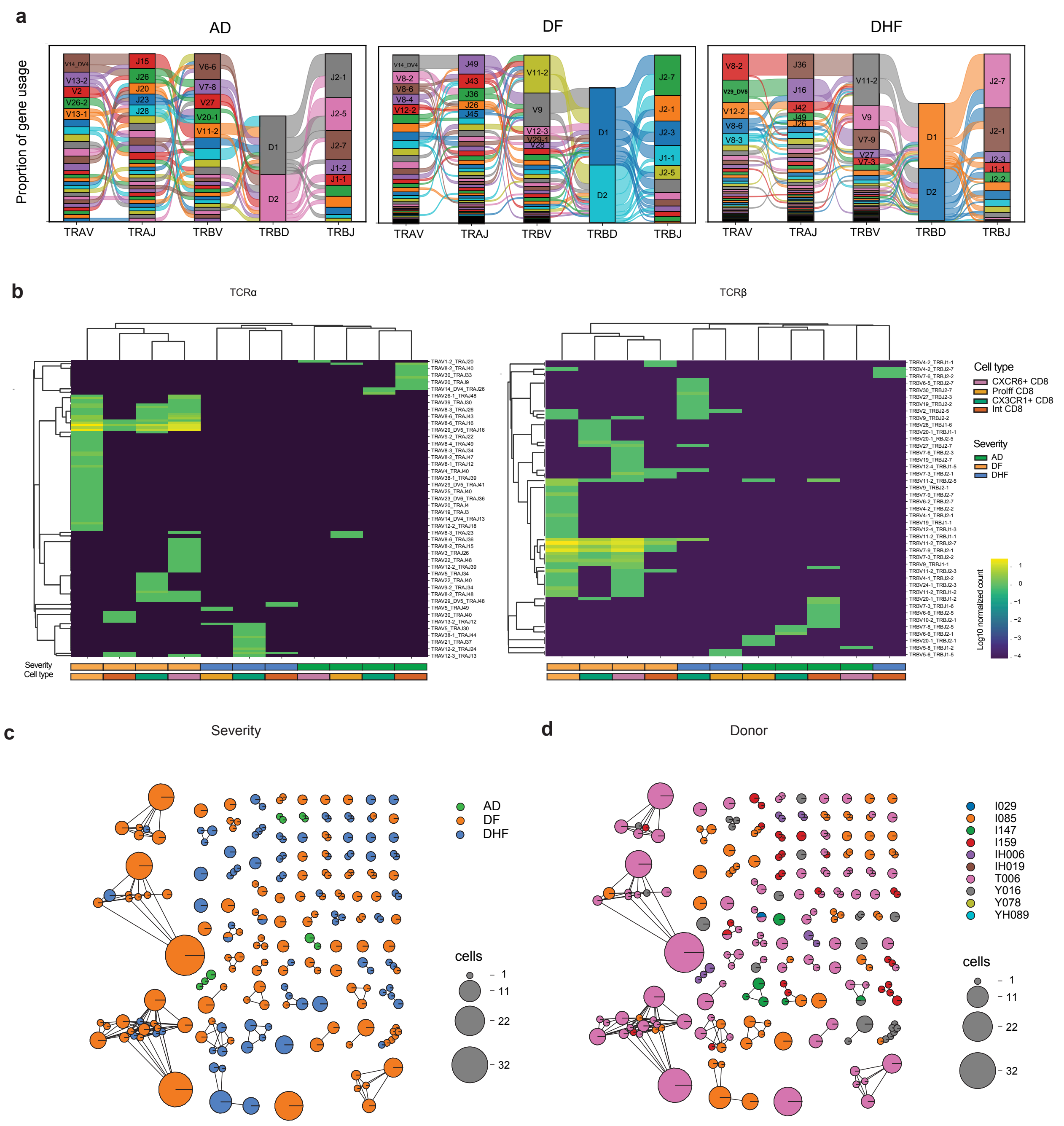

Supplementary Figure 9. TCR repertoire analysis of sorted GTS-specific CD8+ T cells across disease severity. (a) Sankey plots illustrating paired TCR gene segment usage (*TRAV, TRAJ, TRBV, TRBD, TRBJ*) in GTS-specific CD8+ T cells from AD (left), DF (middle), and DHF (right). Line widths indicate the relative frequency of each V(D)J gene pairing. (b) Hierarchically clustered heatmaps of TRAV and TRBV gene usage by clonotype, grouped by TEM CD8+ T cell subset and disease severity during the acute phase. (c–d) Clonotype network plots of GTS-specific CD8+ T cells. Each node represents a unique clonotype, with node size reflecting clonal expansion (number of cells) and edges linking clonotypes with high sequence similarity. In panel c, nodes are colored by disease severity; in panel d, by donor identity.

a

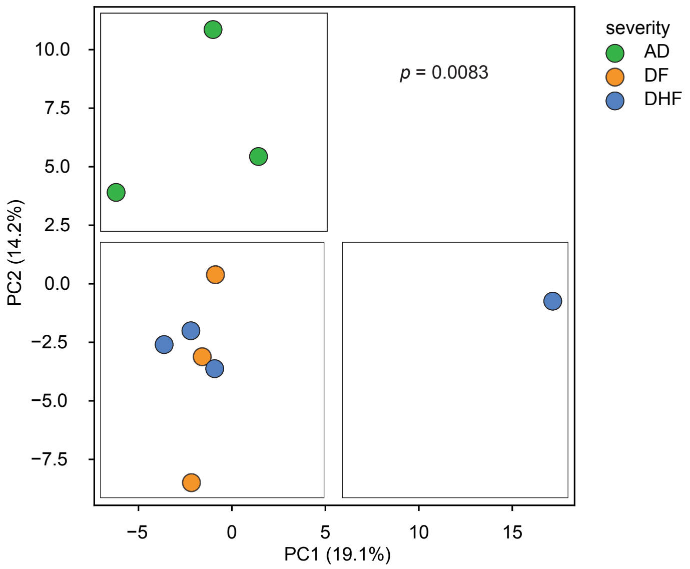

b

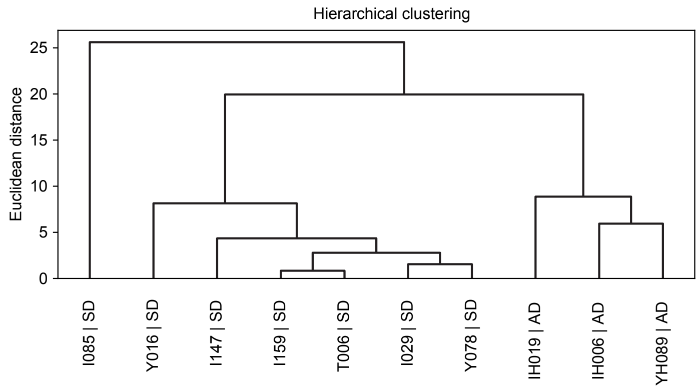

Supplementary Figure 10. Principal component (PCA) and hierarchical clustering analyses of donor-level TCR V(D)J gene usage across dengue disease severity. (a) PCA of donor-level TCR V(D)J gene usage from the integrated dataset combining 10x and SS2 across disease severity groups (AD, DF, DHF). AD samples cluster separately from symptomatic dengue (DF and DHF), which largely overlap in PCA space. Three clusters were defined based on the PCA distribution, and the association between cluster membership and disease severity was evaluated using the Fisher–Freeman–Halton exact test, showing a significant relationship ($p = 0.0083$). (b) Hierarchical clustering dendrogram of donor-level samples based on Euclidean distances calculated from PC1 and PC2 coordinates derived from PCA of global TCRα and TCRβ V(D)J gene usage. Each leaf represents an individual donor labeled by donor identity and disease group. Clustering reveals separation of AD donors from symptomatic dengue (SD; DF and DHF) samples, consistent with the PCA distribution.

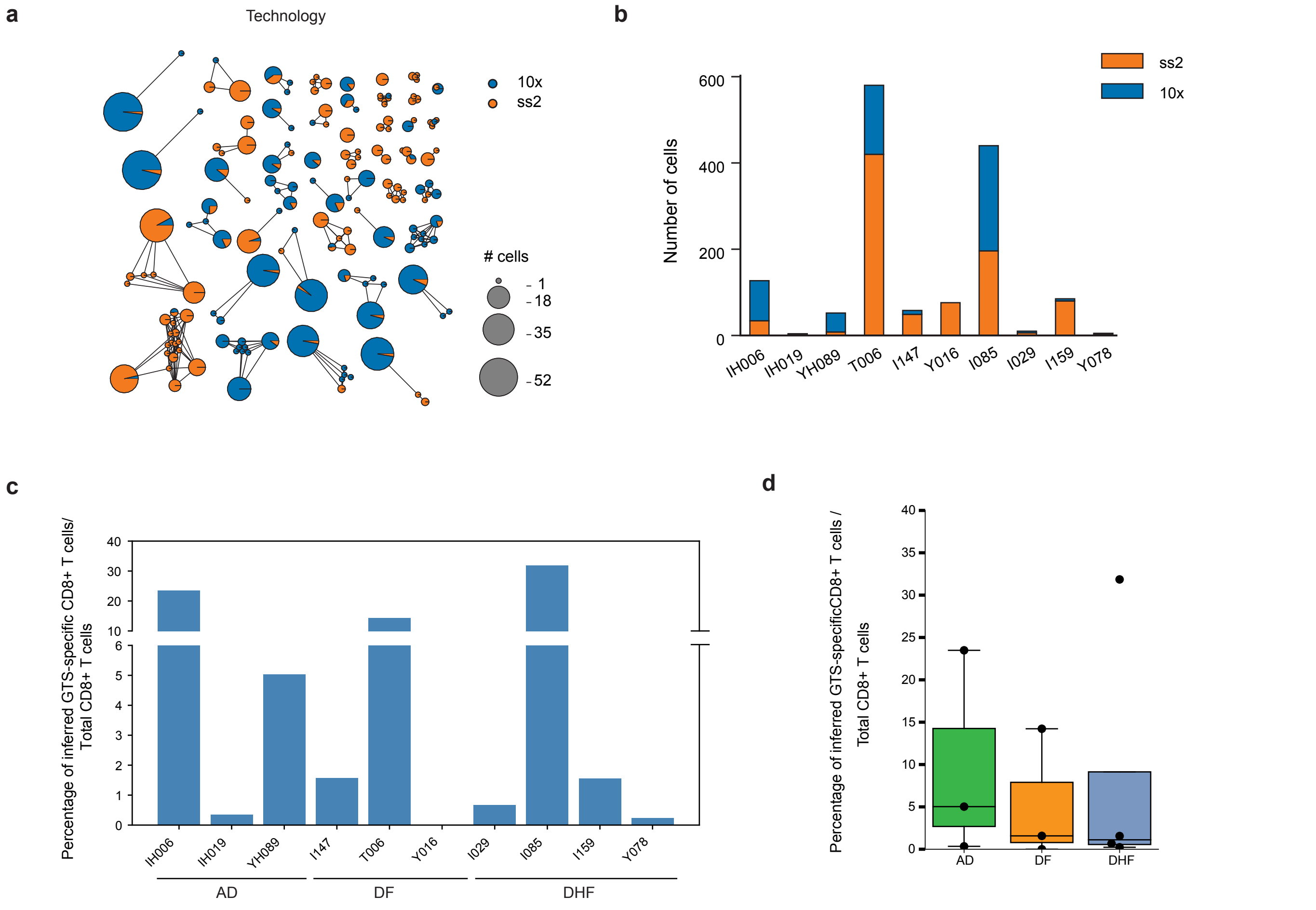

Supplementary Figure 11. Integration of GTS-specific CD8+ T cells from matched HLA-A*11+ donors across two scRNA-seq technologies. (a) Clonotype network plot showing GTS-specific CD8+ T cells from 10x Genomics (blue) and Smart-seq2 (orange) datasets. Each node represents a unique clonotype, with size corresponding to clonal expansion (number of cells). Edges connect clonotypes with high sequence similarity. (b) Stack bar plot showing the number of GTS-specific CD8+ T cells derived from each donor, stratified by scRNA-seq technology. (c) Bar plot showing the percentage of inferred GTS-specific CD8+ T cells relative to total CD8+ T cells for each donor in the 10X dataset. The proportion was calculated based on the number of tetramer-positive cells detected within the total CD8+ T-cell population for each donor. (d) Distribution of the percentage of inferred GTS-specific CD8+ T cells among total CD8+ T cells across disease severity groups in the 10X dataset. Each point represents an individual donor. Kruskal–Wallis test showed no statistically significant differences among groups.

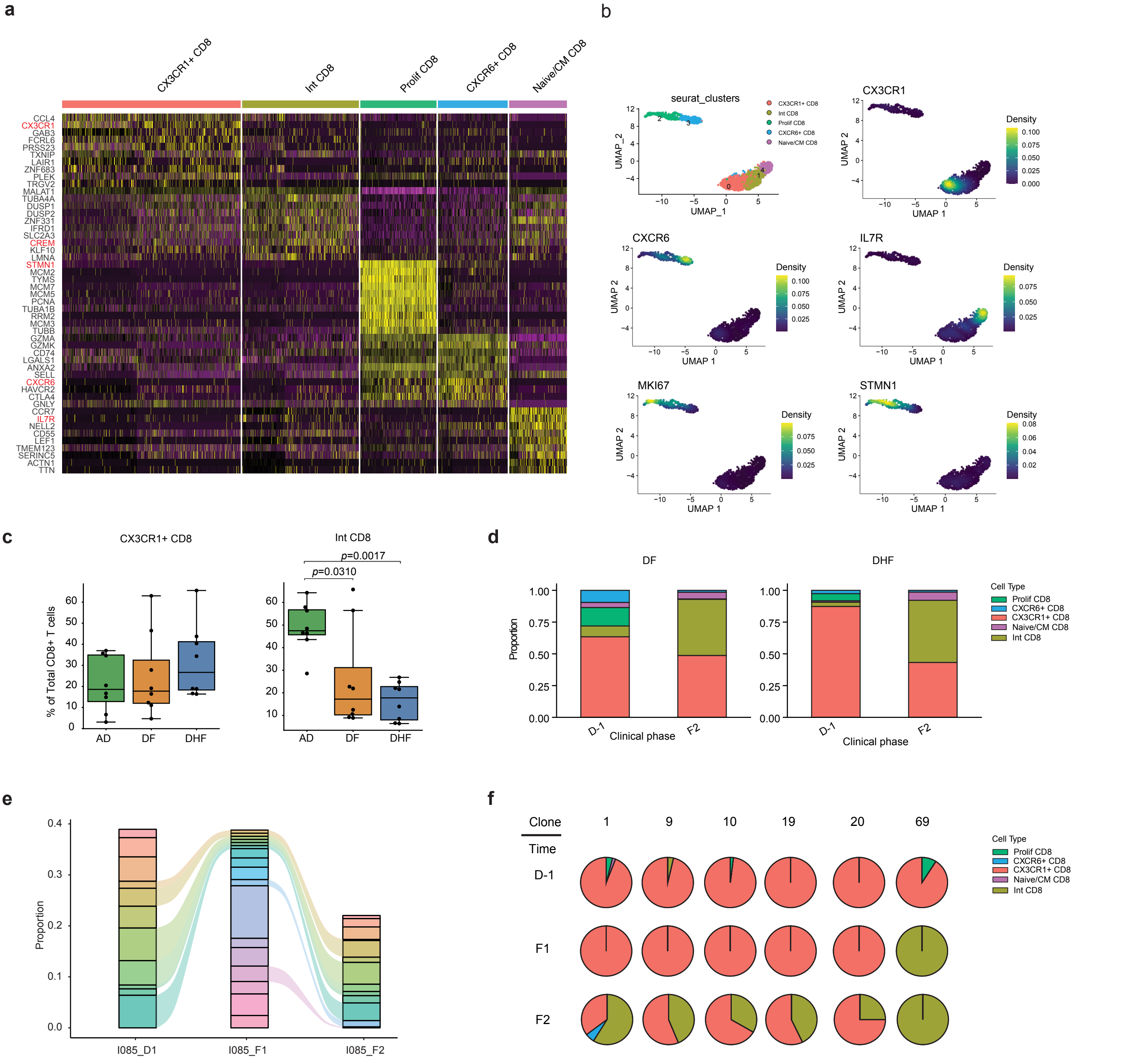

Supplementary Figure 12. Integrated data analysis of GTS-specific CD8+ T cell subsets, longitudinal analysis, and clonal dynamics. (a) Heatmap showing expression of marker genes across GTS-specific CD8+ T cell subsets identified from integrated scRNA-seq analysis. Columns represent distinct subsets. (b) UMAP projection of DENV-specific CD8+ T cells colored by subset identity (top left) and by expression density (right) of key marker genes, including *CX3CR1, CXCR6, IL7R, MKI67*, and *STMN1*, supporting subset classification.(c) Box plots comparing the proportions of total CD8+ T cell subsets, including CX3CR1+ CD8 and Int CD8 subsets, across severity groups (AD = 8, DF = 8, DHF = 8) in the 10x dataset, using GTS-specific CD8+ T cell information from acute dengue infection. Statistical analysis was performed using the Kruskal–Wallis test. No significant differences were observed for CX3CR1+ CD8+ T cells. By contrast, Int CD8+ T cells showed significant differences across severity groups (p = 0.0056), with pairwise results indicated in the graph. (d) Stacked bar plots showing the temporal distribution of clonally expanded CD8+ T cell subsets in DF and DHF (DF = 3, DHF = 3) at day-1 (D-1) and 2-month convalescence (F2) time points in the longitudinal cohort, based on GTS-specific CD8+ T cell information. (e) Alluvial plot showing clonal dynamics of GTS-specific CD8+ T cells from donor I085 across three time points, colored by CD8+ T cell subset. Ribbon links indicate the same clone. (f) Pie charts illustrating the subset composition of representative CD8+ T cell clones from donor I085 that persisted across D-1, F1, and F2 time points. Source data is provided as a Source Data file.

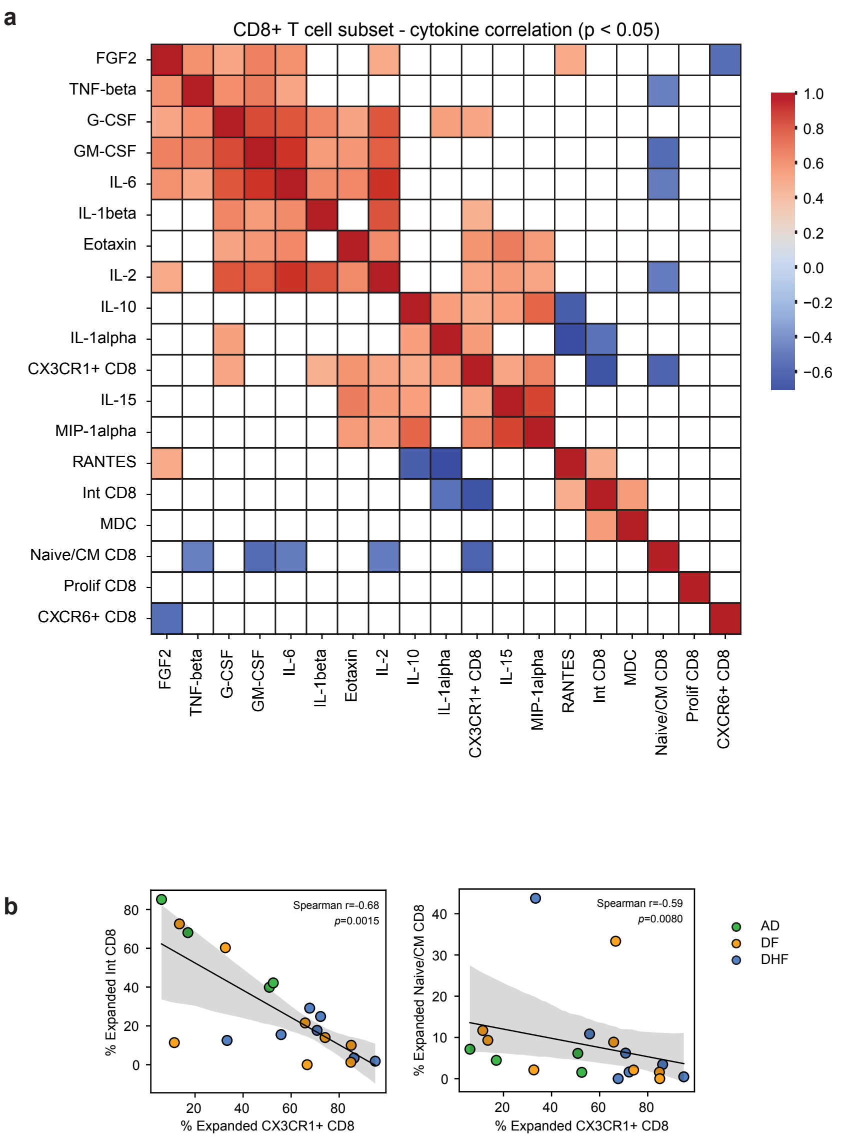

Supplementary Figure 13. Correlation between clonally expanded CD8+ T cell subsets and serum cytokine levels from match donors (AD, n = 4; DF, n = 8; DHF, n = 7) during acute infection. (a) Spearman correlation matrix showing significant correlations ($p < 0.05$) between the frequencies of clonally expanded CD8+ T cell subsets and serum cytokine levels. Positive correlations are shown in red and negative correlations in blue. (b) Scatter plots showing negative correlations between expanded CX3CR1+ CD8+ T cells and expanded Naive/CM CD8+ (left) and Int CD8+ (right) subsets. Each dot represents an individual donor, colored by disease severity group. Linear regression lines with 95% confidence intervals are shown in gray.

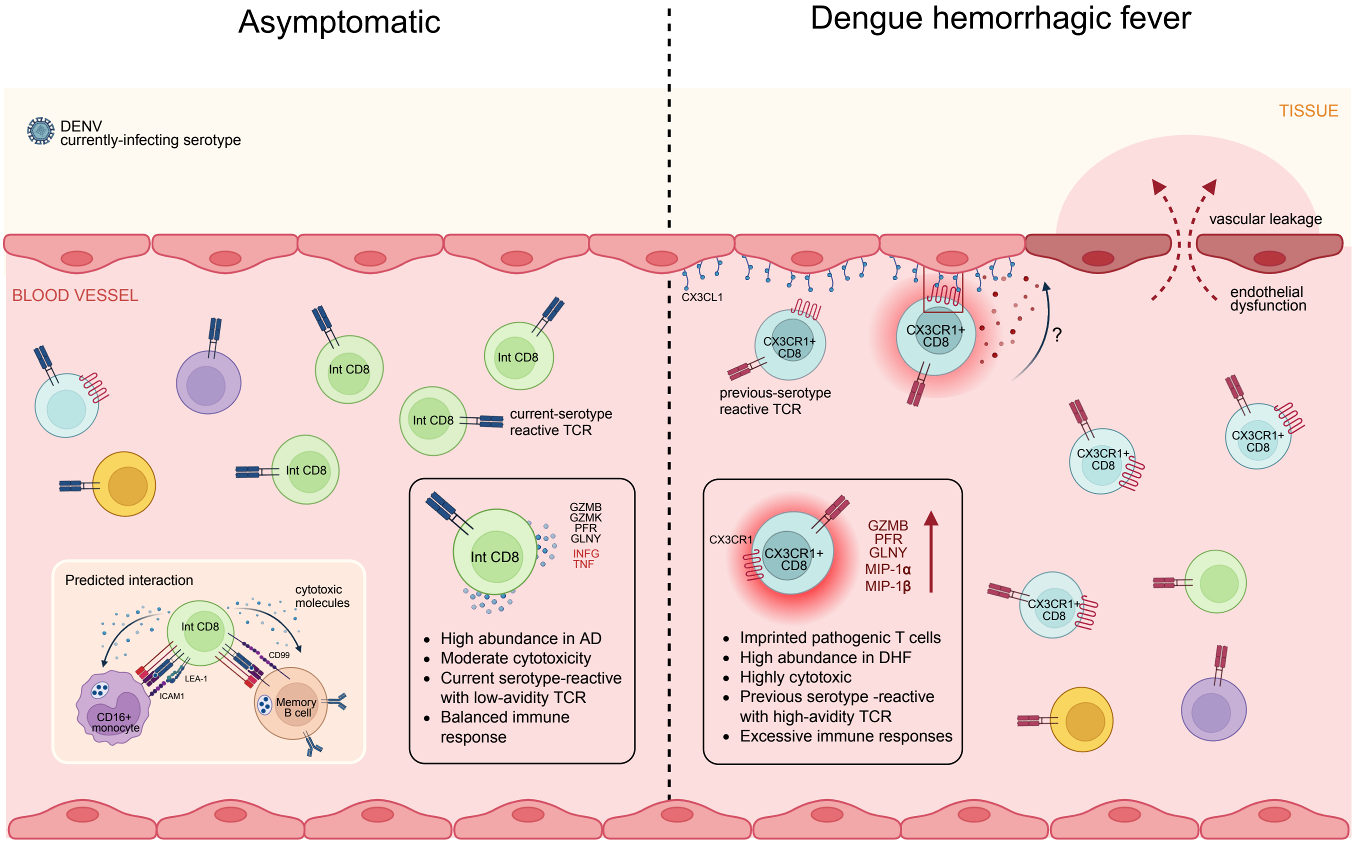

Supplementary Figure 14. Hypothetical model of DENV-specific CD8+ T cell mechanisms in protective versus pathogenic responses during acute dengue infection. (Left) Int CD8+ T cells are highly abundant during acute infection, characterized by moderate cytotoxicity and low-avidity TCRs specific to the current infecting serotype. Predicted interactions suggest these cells can form immunological synapses with viral reservoirs such as CD16+ monocytes and memory B cells, thereby contributing to viral clearance while limiting immunopathology. (Right) CX3CR1+ CD8+ T cells represent imprinted pathogenic T cells that expand during the acute phase. These cells express high-avidity TCRs specific to previously infecting serotypes, display strong cytotoxic activity, and produce pro-inflammatory cytokines that amplify immune activation. Their CX3CR1 expression potentially promotes migration toward the vasculature, where endothelial cells express CX3CL1. Engagement of CX3CR1 on CD8+ T cells with endothelial CX3CL1 leads to release of cytotoxic granules and inflammatory cytokines, driving endothelial activation and dysfunction. The combined effect of these processes contributes to vascular leakage, a hallmark of severe dengue disease. Created in BioRender. Srikor, S. (2026) https://BioRender.com/0hrquvo

Quantification of tetramer fluorescence intensity and statistical analysis

Tetramer fluorescence intensity was quantified by integrating the PE-A and APC-A tetramer channels into a composite tetramer fluorescence index (CTFI). The CTFI was calculated as the Euclidean norm of the two fluorescence signals:

$$CTFI = \sqrt{(PE - A)2 + (APC - A)2}$$

This metric summarizes overall tetramer staining intensity across the two detection channels. For statistical analysis, the CTFI values were log-transformed prior to modeling.

To assess differences in tetramer fluorescence intensity across CD8+ T-cell clusters while accounting for donor-to-donor variability, linear mixed-effects regression models were fitted using the *statsmodels* package in Python. Prior to analysis, donor–cluster combinations represented by fewer than four cells were excluded to avoid unstable estimates arising from extremely small cell counts. The model included cell cluster identity as a fixed effect and donor identity as a random intercept.

$$CTFI_{logij} = \beta_0 + \beta_k(\text{Cell cluster}_k) + u_j + \varepsilon_{ij}$$

Where i indexes individual cells and j indexes donors. Cluster identity (Cell clusters) was included as a categorical fixed effect, and donor ID was modeled as a random intercept ($uj \sim N(0, \sigma^2 donor)$) to account for inter-donor variability. Residual errors were assumed to follow a normal distribution ($\varepsilon ij \sim N(0, \sigma^2)$).

Contingency table analysis and effect size estimation

Contingency tables summarizing the distribution of TCR VJ clusters across disease severity groups were constructed by aggregating cell counts within each cluster. Associations between cluster distribution and disease severity were assessed using the Fisher–Freeman–Halton exact test for RxC contingency tables with Monte Carlo simulation (100,000 iterations). Standardized residuals from the chi-square test were calculated to visualize deviations from expected frequencies. Effect sizes were quantified using Cramer's V, calculated from the chi-square statistic of the contingency table as:

$$v = \sqrt{\frac{\chi 2}{n \times min(r-1, c-1)}}$$

where χ2 is the chi-square statistic, nnn is the total number of observations, and rrr and ccc denote the numbers of rows and columns in the contingency table, respectively.

All contingency table analyses were performed in R. The R scripts used for contingency table construction, Fisher–Freeman–Halton testing with Monte Carlo simulation, standardized residual calculation, and Cramer's V effect size estimation are available at: https://github.com/sirawitsrikor/dengue-gex-tcr-analysis

Table S1: Participant demographics, clinical parameters and applied single-cell RNA-seq techniques

Sample ID	ID	HLA	Severity	Days post-onset of symptoms (DPO)	Serotype	Age	Sex	Viral copies/ml (qRT-PCR)	Primary/ Secondary infection	Ig Titer(HI)								scRNA-seq technique
									HI	Timepoint	DENV1	DENV2	DENV3	DENV4	JEV	Zika	Chik	
IH006	1	A*11	AD	N/A	DENV4	20–29	F	1.03E+07	Secondary	D1	N/A	N/A	N/A	N/A	N/A	N/A	N/A	SS2,10X
IH019	2	A*11	AD	N/A	DENV3	60–69	M	1.64E+06	Secondary	D1	N/A	N/A	N/A	N/A	N/A	N/A	N/A	SS2,10X
IH023	3	A*24	AD	N/A	DENV3	20–29	M	1.17E+04	Secondary	D1	N/A	N/A	N/A	N/A	N/A	N/A	N/A	SS2,10X
YH045	4	A*02	AD	N/A	DENV4	10–19	M	2.19E+06	Unknown	D1	N/A	N/A	N/A	N/A	N/A	N/A	N/A	10X
YH049	5	A*11	AD	N/A	DENV4	30–39	F	5.37E+06	Secondary	D1	N/A	N/A	N/A	N/A	N/A	N/A	N/A	10X
YH089	6	A*11	AD	N/A	DENV1	30–39	F	1.06E+03	Primary	D1	N/A	N/A	N/A	N/A	N/A	N/A	N/A	SS2,10X
H001	7	A*02	AD	N/A	DENV3,4	10–19	F	3.60E+04, 1.47E+07	Secondary	D1	N/A	N/A	N/A	N/A	N/A	N/A	N/A	10X
H030	8	A*02	AD	N/A	DENV3	30–39	M	5.31E+04	Secondary	D1	N/A	N/A	N/A	N/A	N/A	N/A	N/A	10X
I160	9	A*02	DF	3	DENV3	30–39	M	2.76E+04	Secondary	D1	N/A	10240	10240	N/A	5120	N/A	0	10X
T010	10	A*24	DF	5	DENV3	20–29	F	5.34E+03	Secondary	D1	N/A	5120	5120	N/A	10240	2560	0	SS2,10X
T006	11	A*11	DF	4	DENV4	10–19	F	1.77E+06	Secondary	D1	N/A	5120	20480	N/A	5120	N/A	0	SS2,10X
Y029	12	A*24	DF	3	DENV4	60–69	F	7.04E+06	Secondary	D1	N/A	10240	10240	N/A	20480	10240	320	SS2,10X
Y016	13	A*24 & A*11	DF	N/A	DENV4	40–49	F	6.99E+08	Secondary	D1	N/A	160	320	N/A	640	160	0	SS2,10X
I147	14	A*11	DF	5	DENV3	10–19	M	1.96E+07	Secondary	D1	N/A	5120	25560	N/A	2560	N/A	0	SS2,10X
I171	15	A*24	DF	3	DENV4	60–69	F	2.65E+06	Secondary	D1	N/A	5120	20480	N/A	5120	1280	0	SS2,10X
I109	16	A*24	DF	5	DENV1	30–39	M	3.52E+02	Secondary	D1	N/A	10240	20480	N/A	10240	N/A	0	SS2,10X
I085	17	A*11	DHF	3	DENV3	10–19	M	6.87E+03	Secondary	D1	N/A	10240	1520	N/A	5120	N/A	0	SS2,10X
Y078	18	A*24 & A*11	DHF	4	DENV3	20–29	F	3.35E+04	Secondary	D1	N/A	1280	2560	N/A	5120	1280	0	SS2,10X
I010	19	A*02	DHF	4	DENV3	20–29	M	2.03E+07	Secondary	D1	N/A	1280	1280	N/A	1280	N/A	0	10X
I016	20	A*02	DHF	5	DENV4	30–39	F	5.79E+06	Secondary	D1	N/A	20480	20480	N/A	10240	N/A	80	10X
I159	21	A*11	DHF	3	DENV4	30–39	M	1.41E+07	Secondary	D1	N/A	80	80	N/A	40	N/A	0	SS2,10X
I024	22	A*02	DHF	5	DENV4	30–39	F	8.61E+05	Secondary	D1	N/A	5120	5120	N/A	1280	N/A	0	10X
I029	23	A*11	DHF	5	DENV4	20–29	F	1.66E+06	Secondary	D1	N/A	20480	5120	N/A	2560	N/A	0	SS2,10X
I075	24	A*24	DHF	5	DENV1	10–19	F	7.54E+06	Secondary	D1	N/A	10240	20480	N/A	5120	N/A	80	SS2,10X

N/A: Data is not available

Table S2: PRNT results of HLA-A*11 and HLA-A*24 patients

Sample ID	Severity	Current serotype	PRNT50 titer					PRNT infered previous serotype
			Time-point	DENV1	DENV2	DENV3	DENV4	
IH006	AD	DENV4	D1	1031	7105	511	382	DENV2
IH019	AD	DENV3	D1	56	1104	735	551	DENV2
IH023	AD	DENV3	D1	404	1713	1399	1254	DENV2
YH089	AD	DENV1	D1	1229	55	33	0	Unknown
T010	DF	DENV3	D1	3590	7424	1652	54194	DENV4
			F1	5944	16706	7618	38598	
T006	DF	DENV4	D1	42206	30915	17840	16113	DENV1
			F1	308937	129567	45104	37607	
Y029	DF	DENV4	D1	748	43559	3928	4632	DENV2
			F1	41244	20879	28983	38806	
Y016	DF	DENV4	D1	N/A	N/A	N/A	N/A	N/A
			F1	N/A	N/A	N/A	N/A	
I147	DF	DENV3	D1	2108	13286	2960	2555	DENV2
			F1	6350	372805	26783	61984	
I171	DF	DENV4	D1	8140	20770	27268	13709	Unknown
			F1	42881	29865	38709	86973	
I109	DF	DENV1	D1	4947	3646	33863	23419	DENV3
			F1	8439	14483	26914	45461	
I085	DHF	DENV3	D1	25703	64353	3004	14401	DENV2
			F1	6365	136271	4443	41112	
Y078	DHF	DENV3	D1	1558	4923	1582	6366	DENV4
I159	DHF	DENV4	D1	9	315	48	40	DENV2
			F1	7127	51718	4797	25201	
I029	DHF	DENV4	D1	6148	11956	6739	4291	DENV2
I075	DHF	DENV1	D1	3423	41067	4533	340	DENV2
			F1	19546	17044	142196	26523	

Red = Titer of the current infecting-serotype
N/A: Data is not available
Unknown: Data is unable to interprete due to low titer

Table S3. Peptide sequences used for pHLA tetramer generation

HLA restriction	Epitope variant	Serotype	Amino acid sequence	Protein	Position
HLA-A*11	GTS1.1	DENV1	GTSGSPIVNR	NS3	133–142
HLA-A*11	GTS2.1	DENV2	GTSGSPIIDK	NS3	133–142
HLA-A*11	GTS3.1	DENV3/4	GTSGSPIINR	NS3	133–142
HLA-A*24	NYA1.1	DENV1	QYSDRRWCF	NS3	556–564
HLA-A*24	NYA2.1	DENV2	NYADRRWCF	NS3	556–564
HLA-A*24	NYA3.1	DENV3	KYTDRKWCF	NS3	556–564
HLA-A*24	NYA4.1	DENV4	SYKDREWCF	NS3	556–564

Table S4: Number of sorted CD8+ T cells from all samples

Sample ID	Severity	HLA	Epitope staining	Clinical_phase	Epitope binding (#cells)			
					double-positive	current	previous	Total
IH006	AD	A*11	GTS	Acute	0	53	0	53
IH019	AD	A*11	GTS	Acute	2	5	2	9
YH089	AD	A*11	GTS	Acute	0	0	17	17
I147	DF	A*11	GTS	Acute	1	4	3	8
T006	DF	A*11	GTS	Acute	356	39	64	459
Y016	DF	A*24 & A*11	GTS	Acute	0	1	0	1
I029	DHF	A*11	GTS	Acute	0	17	1	18
I085	DHF	A*11	GTS	Acute	14	6	38	58
I159	DHF	A*11	GTS	Acute	0	0	1	1
Y078	DHF	A*24 & A*11	GTS	Acute	0	4	0	4
I147	DF	A*11	GTS	Convalescence	12	60	3	75
T006	DF	A*11	GTS	Convalescence	52	43	35	130
Y016	DF	A*24 & A*11	GTS	Convalescence	113	34	37	184
I085	DHF	A*11	GTS	Convalescence	153	144	36	333
I159	DHF	A*11	GTS	Convalescence	16	104	4	124
IH023	AD	A*24	NYA	Acute	8	7	10	25
I109	DF	A*24	NYA	Acute	0	19	3	22
I171	DF	A*24	NYA	Acute	2	27	132	161
T010	DF	A*24	NYA	Acute	1	4	66	71
Y029	DF	A*24	NYA	Acute	16	73	2	91
I075	DHF	A*24	NYA	Acute	1	20	14	35
I109	DF	A*24	NYA	Convalescence	23	75	11	109
I171	DF	A*24	NYA	Convalescence	32	39	185	256
T010	DF	A*24	NYA	Convalescence	3	1	19	23
Y029	DF	A*24	NYA	Convalescence	0	69	36	105
I075	DHF	A*24	NYA	Convalescence	4	7	14	25
**Total**								2397

Table S5: Number of antigen-specific CD8+ T cells included in gene expressione analysis by donor

Sample ID	Severity	HLA	clinical phase	cell count
IH006	AD	A*11	Acute	51
IH023	AD	A*24	Acute	14
YH089	AD	A*11	Acute	9
I109	DF	A*24	Acute	12
I147	DF	A*11	Acute	3
I171	DF	A*24	Acute	154
T006	DF	A*11	Acute	382
T010	DF	A*24	Acute	10
Y029	DF	A*24	Acute	70
I029	DHF	A*11	Acute	9
I075	DHF	A*24	Acute	22
I085	DHF	A*11	Acute	38
I159	DHF	A*11	Acute	1
Y078	DHF	A*24 & A*11	Acute	1
I109	DF	A*24	Convalescence	56
I147	DF	A*11	Convalescence	54
I171	DF	A*24	Convalescence	217
T006	DF	A*11	Convalescence	101
T010	DF	A*24	Convalescence	9
Y016	DF	A*24 & A*11	Convalescence	161
Y029	DF	A*24	Convalescence	70
I075	DHF	A*24	Convalescence	23
I085	DHF	A*11	Convalescence	243
I159	DHF	A*11	Convalescence	95
**Total**				1805

Table S6: Number of sorted and inferred DENV-specific CD8+ T cells included in TCR repertoire analysis across HLA-A*11+ donors.

**Sample ID**	**Severity**	**Clinical phase**	**cell count**		
			**Sorted CD8+ T cell (SS2)**	**Inferred DENV-specific CD8+ T cell (10x)**	**Total cell in analysis**
IH006	AD	Acute	34	93	127
IH019	AD	Acute	3	1	4
YH089	AD	Acute	8	44	52
I147	DF	Acute	6	9	15
I147	DF	Convalescence	43	0	43
T006	DF	Acute	344	160	504
T006	DF	Convalescence	76	0	76
Y016	DF	Convalescence	76	0	76
I029	DHF	Acute	6	4	10
I085	DHF	Acute	30	244	274
I085	DHF	Convalescence	166	0	166
I159	DHF	Acute	0	5	5
I159	DHF	Convalescence	80	0	80
Y078	DHF	Acute	3	2	5
**Total**			875	562	1437

Table S7: Demographics and clinical characteristics of participants in the flow cytometry validation cohort

Sample ID	HLA	Severity	Serotype	Age	Sex	Viral copies/ml (qRT-PCR)	Primary/ Secondary infection	Ig Titer(HI)							
							HI	Timepoint	DENV1	DENV2	DENV3	DENV4	JEV	Zika	Chik
I147	A*11	DF	DENV3	10–19	M	1.96E+07	Secondary	D1	N/A	5120	25560	N/A	2560	N/A	0
I153	A*11	DF	DENV3	30–39	F	5.44E+02	Secondary	D1	N/A	5120	2560	N/A	5120	N/A	0
I167	A*11	DF	DENV3	10–19	F	6.21E+09	Secondary	D1	N/A	640	1280	N/A	1280	640	0
T003	A*11	DF	DENV3	30–39	F	2.41E+05	Secondary	D1	N/A	20480	10240	N/A	20480	N/A	0
T008	A*11	DF	DENV4	50–59	F	N/A	Secondary	D1	N/A	20480	20480	N/A	20480	10240	0
Y011	A*11	DF	DENV4	20–29	F	4.79E+07	Secondary	D1	N/A	160	160	N/A	320	160	0
Y030	A*11	DF	DENV4	10–19	F	8.99E+05	Secondary	D1	N/A	2560	2560	N/A	5120	2560	0
Y048	A*11	DF	DENV4	20–29	M	1.29E+07	Secondary	D1	N/A	640	640	N/A	1280	640	0
Y066	A*11	DF	DENV2	30–39	M	5.71E+07	Secondary	D1	N/A	20480	20480	N/A	20480	20480	80
Y074	A*11	DF	DENV4	0–9	F	6.02E+06	Secondary	D1	N/A	160	320	N/A	160	80	0
Y028	A*11	DF	DENV4	50–59	M	N/A	Secondary	D1	N/A	1280	640	N/A	1280	640	0
I002	A*11	DHF	DENV4	20–29	M	4.98E+07	Primary	D1	N/A	40	80	N/A	40	N/A	0
I037	A*11	DHF	DENV3	10–19	M	2.09E+05	Primary	D1	N/A	0	10	N/A	10	N/A	0
I039	A*11	DHF	DENV4	50–59	F	2.64E+06	Secondary	D1	N/A	2560	2560	N/A	1280	N/A	0
I043	A*11	DHF	DENV3	20–29	M	1.26E+09	Secondary	D1	N/A	20480	10240	N/A	5120	N/A	0
I064	A*11	DHF	DENV3	30–39	M	N/A	Secondary	D1	N/A	2560	5120	N/A	2560	N/A	620
I076	A*11	DHF	DENV4	20–29	M	1.74E+07	Secondary	D1	N/A	20480	20480	N/A	10240	N/A	20
I081	A*11	DHF	DENV3	10–19	F	1.00E+09	Secondary	D1	N/A	320	640	N/A	640	N/A	80
I085	A*11	DHF	DENV3	10–19	M	6.87E+03	Secondary	D1	N/A	10240	1520	N/A	5120	N/A	0
Y023	A*11	DHF	DENV4	30–39	F	9.50E+04	Secondary	D1	N/A	20480	20480	N/A	20480	20480	10

N/A: Data is not available

Table S8: PRNT50 titers against DENV1–4 and distribution of single-positive and double-positive GTS-specific CD8+ T cells across HLA-A*11 donors

Sample ID	Severity	PRNT50 titer				
		Time-point	DENV1	DENV2	DENV3	DENV4
I147	DF	D1	2108	13286	2960	2555
		F1	6350	372805	26783	61984
T006	DF	D1	42206	30915	17840	16113
		F1	308937	129567	45104	37607
Y016	DF	D1	N/A	N/A	N/A	N/A
		F1	N/A	N/A	N/A	N/A
I085	DHF	D1	25703	64353	3004	14401
		F1	6365	136271	4443	41112
I159	DHF	D1	9	315	48	40
		F1	7127	51718	4797	25201
Y078	DHF	D1	1558	4923	1582	6366
		F1	N/A	N/A	N/A	N/A
I029	DHF	D1	6148	11956	6739	4291
		F1	N/A	N/A	N/A	N/A

High

Low

Current

Inferred previous

Sample ID	Percent of CD8 T cells					
	Single-positive			Double-positive		
	Tet-GTS1	Tet-GTS2	Tet-GTS3/4	Tet-GTS1|2	Tet-GTS1|3/4	Tet-GTS2|3/4
I147D0	0.387	0.035	0.228	0.121	0.314	0.125
T006D0	0.65	0.225	1.397	1.158	1.437	0.467
Y016D0	0.71	0.098	0.177	0.095	0.212	0.084
I085D0	0.215	0.224	0.753	0.633	1.272	0.615
I159D0	1.74	0.185	0.188	0.065	0.105	0.062
Y078D0	0.316	0.066	0.199	0.036	0.081	0.01
I029D0	0.169	0.041	0.813	0.503	1.152	0.498

Table S9: PRNT50 titers against DENV1–4 and distribution of single-positive and double-positive NYA-specific CD8+ T cells across HLA-A*24 donors

Sample ID	Severity	PRNT50 titer				
		Time-point	DENV1	DENV2	DENV3	DENV4
IH023	AD	D1	404	1713	1399	1254
		F1	N/A	N/A	N/A	N/A
T010	DF	D1	3590	7424	1652	54194
		F1	5944	16706	7618	38598
Y029	DF	D1	748	43559	3928	4632
		F1	41244	20879	28983	38806
I109	DF	D1	4947	3646	33863	23419
		F1	8439	14483	26914	45461
I171	DF	D1	8140	20770	27268	13709
		F1	42881	29865	38709	86973
I075	DHF	D1	3423	41067	4533	340
		F1	19546	17044	142196	26523

High

Low

Current
Inferred previous

Sample ID	Percent of CD8 T cells									
	Single-positive				Double-positive					
	Tet-NYA1+	Tet-NYA2+	Tet-NYA3+	Tet-NYA4+	Tet-NYA1|2	Tet-NYA1|3	Tet-NYA1|4	Tet-NYA2|3	Tet-NYA2|4	Tet-NYA3|4
IH023	0.011	0.359	0.378	0.452	0.005	0.005	0.005	0.183	0.21	0.279
T010D0	0	0.234	0.315	3.098	0.019	0.019	0.017	2.895	3.358	3.342
Y029D0	0	0.185	0.539	2.797	0.002	0.002	0.002	0.487	0.613	0.913
I109D0	0	0.211	0.369	3.121	0.018	0.019	0.019	2.448	2.994	2.8
I171D0	0.012	2.08	0.844	1.024	0.009	0.009	0.009	3.696	0.691	0.685
I075D0	0	0.179	0.28	1.224	0.016	0.006	0.003	0.58	0.726	0.721

Table S10: cDNA and library concentrations of all plate

HLA-A*11 samples				HLA-A*24 samples			
**Plate ID**	**Library conc. (nM)**	**Assigned Pool**	**Pooled conc. (nM)**	**Plate ID**	**Library conc. (nM)**	**Assigned Pool**	**Pooled conc. (nM)**
Plate 4	274.6	Pool 1 (P4–7)	48.3	Plate 1	12.5	Pool 4 (P1,10)	27.3
Plate 6	272	Pool 1 (P4–7)	48.3	Plate 2	85.5	Pool 1 (P2,5,6,12)	45.3
Plate 7	202.5	Pool 1 (P4–7)	48.3	Plate 3	14.7	Pool 3 (P3,4,7,8)	40.1
Plate 8	236.5	Pool 1 (P4–7)	48.3	Plate 4	84.5	Pool 3 (P3,4,7,8)	40.1
Plate 9	174.5	Pool 2 (P8–11)	33.1	Plate 5	25.7	Pool 1 (P2,5,6,12)	45.3
Plate 10	42.6	Pool 2 (P8–11)	33.1	Plate 6	62.5	Pool 1 (P2,5,6,12)	45.3
Plate 11	14.5	Pool 2 (P8–11)	33.1	Plate 7	107.5	Pool 3 (P3,4,7,8)	40.1
Plate 12	36.9	Pool 3 (P12–15)	34.1	Plate 8	45.4	Pool 3 (P3,4,7,8)	40.1
Plate 13	142.5	Pool 3 (P12–15)	34.1	Plate 9	44.8	Pool 2 (P9,11,13,14)	53.9
Plate 14	41.2	Pool 3 (P12–15)	34.1	Plate 10	58.5	Pool 4 (P1,10)	27.3
Plate 15	241	Pool 3 (P12–15)	34.1	Plate 11	82.5	Pool 2 (P9,11,13,14)	53.9
Plate 16	226	Pool 4 (P16–19)	33.5	Plate 12	27.7	Pool 1 (P2,5,6,12)	45.3
Plate 17	210.5	Pool 4 (P16–19)	33.5	Plate 13	41.9	Pool 2 (P9,11,13,14)	53.9
Plate 18	260.5	Pool 4 (P16–19)	33.5	Plate 14	29.1	Pool 2 (P9,11,13,14)	53.9
Plate 19	82.5	Pool 4 (P16–19)	33.5				
Plate 20	214.5	Pool 5 (P20–23)	34.5				
Plate 21	196.5	Pool 5 (P20–23)	34.5				
Plate 22	196.5	Pool 5 (P20–23)	34.5				
Plate 23	143.5	Pool 5 (P20–23)	34.5				
Plate 24	162.5	Pool 6 (P24–26)	26.6				
Plate 25	97.5	Pool 6 (P24–26)	26.6				

Table S11. Antibody clones used in this study

Product description	Clone	Supplier	Cat. No.	Note
Mouse Anti-Human CD3	SK7	BD Biosciences	347344	sorting/validation
Mouse Anti-Human CD8	SK1	BD Biosciences	560179	sorting/validation
Mouse Anti-Human CD4	RPA-T4	BD Biosciences	555346	sorting
Mouse Anti-Human CD14	M5E2	BD Biosciences	555397	sorting
Mouse Anti-Human CD19	HIB19	BD Biosciences	555412	sorting
Mouse Anti-Human CD56	B159	BD Biosciences	562794	sorting
Mouse Anti-Human CD45RO	UCHL1	BD Biosciences	562641	validation
Mouse Anti-Human CD45RA	HI100	BD Biosciences	563031	validation
Mouse Anti-Human CCR7 (CD197)	2-L1-A	BD Biosciences	566758	validation
Mouse Anti-human CD186 (CXCR6)	K041E5	BioLegend	356020	validation
Rat Anti-human CX3CR1	2A9-1	BioLegend	341612	validation
