# Supplementary material for: Heterogeneity and dynamics of DENV-specific CD8 + T cells in dengue infection
Source: Nat Commun. 2026 Jun 3;17:7103. doi: 10.1038/s41467-026-73491-5 (PMC13392236; doi:10.1038/s41467-026-73491-5)
Supplement: Supplementary file 3 — Reporting Summary [file 41467_2026_73491_MOESM3_ESM.pdf]

nature portfolio

Corresponding author(s): Ponpan Matangkasombut

Last updated by author(s): May 7, 2026

Reporting Summary

Nature Portfolio wishes to improve the reproducibility of the work that we publish. This form provides structure for consistency and transparency in reporting. For further information on Nature Portfolio policies, see our Editorial Policies and the Editorial Policy Checklist.

Statistics

For all statistical analyses, confirm that the following items are present in the figure legend, table legend, main text, or Methods section.

n/a	Confirmed	
☐	☒	The exact sample size (*n*) for each experimental group/condition, given as a discrete number and unit of measurement
☐	☒	A statement on whether measurements were taken from distinct samples or whether the same sample was measured repeatedly
☐	☒	The statistical test(s) used AND whether they are one- or two-sided<br>*Only common tests should be described solely by name; describe more complex techniques in the Methods section.*
☐	☒	A description of all covariates tested
☐	☒	A description of any assumptions or corrections, such as tests of normality and adjustment for multiple comparisons
☐	☒	A full description of the statistical parameters including central tendency (e.g. means) or other basic estimates (e.g. regression coefficient) AND variation (e.g. standard deviation) or associated estimates of uncertainty (e.g. confidence intervals)
☐	☒	For null hypothesis testing, the test statistic (e.g. *F*, *t*, *r*) with confidence intervals, effect sizes, degrees of freedom and *P* value noted<br>*Give P values as exact values whenever suitable.*
☒	☐	For Bayesian analysis, information on the choice of priors and Markov chain Monte Carlo settings
☒	☐	For hierarchical and complex designs, identification of the appropriate level for tests and full reporting of outcomes
☐	☒	Estimates of effect sizes (e.g. Cohen's *d*, Pearson's *r*), indicating how they were calculated

Our web collection on statistics for biologists contains articles on many of the points above.

Software and code

Policy information about availability of computer code

Data collection	Flow cytometry data collected with BD FACSAria™ III Cell Sorter and BD FACSMelody™ Cell Sorter, visualized by FlowJo v10.10.
Data analysis	scRNA-seq analyzed using Scanpy (v1.8.2), Seurat (v4.3.0), Scirpy (v 0.10.1), TraCeR and CellChat (v1.6.1) | Visualization: matplotlib, seaborn, ggplot2 | Code availability: https://github.com/sirawitsrikor/dengue-gex-tcr-analysis

For manuscripts utilizing custom algorithms or software that are central to the research but not yet described in published literature, software must be made available to editors and reviewers. We strongly encourage code deposition in a community repository (e.g. GitHub). See the Nature Portfolio guidelines for submitting code & software for further information.

Data

Policy information about availability of data

All manuscripts must include a data availability statement. This statement should provide the following information, where applicable:

- Accession codes, unique identifiers, or web links for publicly available datasets
- A description of any restrictions on data availability
- For clinical datasets or third party data, please ensure that the statement adheres to our policy

The raw single-cell RNA sequencing and TCR sequencing data generated in this study have been deposited in the European Genome-Phenome Archive (EGA) under accession code EGAD00001015637. The data are available under controlled access due to ethical and privacy considerations for human participants, and can be obtained by application to the relevant data access committee through the EGA repository. Raw sequencing data are protected and not publicly available due to data privacy regulations.

Research involving human participants, their data, or biological material

Policy information about studies with human participants or human data. See also policy information about sex, gender (identity/presentation), and sexual orientation and race, ethnicity and racism.

Reporting on sex and gender	Both male and female donors were included in the study, with sex recorded at enrollment based on self-reporting. No significant sex-based differences were observed in the analyses performed. Gender identity was not specifically collected.
Reporting on race, ethnicity, or other socially relevant groupings	All participants were Thai nationals, recruited in Bangkok and Tak provinces, Thailand. Nationality/ethnicity was determined based on self-reporting at enrollment. Race or ethnicity was not used as a study variable in the analyses, and no stratification was performed on this basis. Analyses focused on clinical severity and infection phase, and potential confounding by race/ethnicity was not applicable in this study.
Population characteristics	The study included dengue virus–infected participants spanning three clinical categories: asymptomatic dengue (AD), dengue fever (DF), and dengue hemorrhagic fever (DHF), diagnosed according to WHO criteria. Participants were recruited in Bangkok and Tak provinces, Thailand. Age and sex were balanced across severity groups, with both male and female donors included.
Recruitment	Participants were recruited from the DENFREE Thailand cohort at Vajira Hospital (Bangkok) and Tasongyang Hospital (Tak province). Symptomatic dengue (SD) patients were enrolled upon hospital presentation, while asymptomatic dengue cases were identified through household surveillance of SD index cases. Recruitment was based on virologically confirmed dengue infection. Potential selection bias is limited to the geographic regions of recruitment, but is unlikely to affect the internal comparisons across disease severity groups.
Ethics oversight	The study protocol was approved by the Institutional Review Boards of Vajira Hospital (No. 015/12), Mahidol University Ethics Committee (TMEC 13-041), and Ramathibodi Hospital (MURA2016/219 and MURA2019/603), as well as the UK North West–York Research Ethics Committee (19/NE/0170).

Note that full information on the approval of the study protocol must also be provided in the manuscript.

Field-specific reporting

Please select the one below that is the best fit for your research. If you are not sure, read the appropriate sections before making your selection.

☒ Life sciences ☐ Behavioural & social sciences ☐ Ecological, evolutionary & environmental sciences

For a reference copy of the document with all sections, see nature.com/documents/nr-reporting-summary-flat.pdf

Life sciences study design

All studies must disclose on these points even when the disclosure is negative.

Sample size	Sorted tetramer+ CD8+ T cells were obtained from 4 AD, 7 DF, and 5 DHF donors in the acute phase, and from 7 DF and 3 DHF donors in the convalescent phase. An independent flow cytometry validation cohort included 11 DF and 9 DHF donors.
Data exclusions	Cells were excluded if they failed quality control filters, including fewer than 200 detected genes, >17.5% mitochondrial content, or extreme read counts. Pre-established criteria were applied uniformly across all samples. In addition, donor samples with fewer than four high-quality cells were excluded from statistical analyses.
Replication	N/A
Randomization	N/A
Blinding	N/A

Reporting for specific materials, systems and methods

We require information from authors about some types of materials, experimental systems and methods used in many studies. Here, indicate whether each material, system or method listed is relevant to your study. If you are not sure if a list item applies to your research, read the appropriate section before selecting a response.

Materials & experimental systems

n/a	Involved in the study	
☐	☒	Antibodies
☒	☐	Eukaryotic cell lines
☒	☐	Palaeontology and archaeology
☒	☐	Animals and other organisms
☐	☒	Clinical data
☒	☐	Dual use research of concern
☒	☐	Plants

Methods

n/a	Involved in the study	
☒	☐	ChIP-seq
☐	☒	Flow cytometry
☒	☐	MRI-based neuroimaging

Antibodies

Antibodies used

Mouse Anti-Human CD3 PerCP, BD Biosciences, clone SK7, cat: 347344, lot# 2363532
Mouse Anti-Human CD8 APC-H7, BD Biosciences, clone SK1, cat: 560179, lot# 1039448
Mouse Anti-Human CD4 FITC, BD Biosciences, clone RPA-T4, cat: 555346, lot# 30292
Mouse Anti-Human CD14 FITC, BD Biosciences, clone M5E2, cat: 555397, lot# 7289545
Mouse Anti-Human CD19 FITC, BD Biosciences, clone HIB19, cat: 555412, lot# 7208855
Mouse Anti-Human CD56 FITC, BD Biosciences, clone B159, cat: 562794, lot# 0010250
Mouse Anti-Human CD45RA BV510, BD Biosciences, clone HI100, cat: 563031, lot# 3244605
Mouse Anti-Human CD45RO BV421, BD Biosciences, clone UCHL1, cat: 562641, lot# 3177655
Mouse Anti-Human CCR7 BV786, BD Biosciences, clone 2-L1-A, cat: 566758, lot# 3172743
Mouse Anti-Human CXCR6 FITC, BioLegend, clone K041E5, cat: 356020, lot# B388931
Rat Anti-Human CX3CR1 PE/Cy7, BioLegend, clone 2A9-1, cat: 341612, lot# B349469

Validation

All antibodies used in this study have been validated by the manufacturers for human flow cytometry applications, with validation data available on the respective manufacturers' websites. pHLA monomers were kindly provided by Dr. Juthatip Mongkolsapaya (University of Oxford) and had been validated and used in her team's previously published studies (Mongkolsapaya et al., Nat Med 2003; Mongkolsapaya et al., J Immunol 2006).

Clinical data

Policy information about clinical studies
All manuscripts should comply with the ICMJE guidelines for publication of clinical research and a completed CONSORT checklist must be included with all submissions.

Clinical trial registration: N/A

Study protocol: N/A

Data collection: N/A

Outcomes: N/A

Plants

Seed stocks

Report on the source of all seed stocks or other plant material used. If applicable, state the seed stock centre and catalogue number. If plant specimens were collected from the field, describe the collection location, date and sampling procedures.

Novel plant genotypes

Describe the methods by which all novel plant genotypes were produced. This includes those generated by transgenic approaches, gene editing, chemical/radiation-based mutagenesis and hybridization. For transgenic lines, describe the transformation method, the number of independent lines analyzed and the generation upon which experiments were performed. For gene-edited lines, describe the editor used, the endogenous sequence targeted for editing, the targeting guide RNA sequence (if applicable) and how the editor was applied.

Authentication

Describe any authentication procedures for each seed stock used or novel genotype generated. Describe any experiments used to assess the effect of a mutation and, where applicable, how potential secondary effects (e.g. second site T-DNA insertions, mosiacism, off-target gene editing) were examined.

Flow Cytometry

Plots

Confirm that:

- [x] The axis labels state the marker and fluorochrome used (e.g. CD4-FITC).
- [x] The axis scales are clearly visible. Include numbers along axes only for bottom left plot of group (a 'group' is an analysis of identical markers).
- [x] All plots are contour plots with outliers or pseudocolor plots.
- [x] A numerical value for number of cells or percentage (with statistics) is provided.

Methodology

Sample preparation	PBMCs were thawed, stained with LIVE/DEAD viability dye, incubated with peptide-loaded HLA class I tetramers (PE- or APC-conjugated), and subsequently stained with antibody panels. For the independent flow cytometry validation cohort, PBMCs were thawed and incubated with a protein kinase inhibitor prior to tetramer and antibody staining, without LIVE/DEAD viability dye. To assess tetramer specificity across the four DENV serotypes, PBMC samples were first stained with LIVE/DEAD viability dye and treated with PKI prior to multi-tetramer staining.
Instrument	BD FACSAria III (sorting) and BD FACSMelody (validation)
Software	FlowJo v10.10 (BD Biosciences)
Cell population abundance	Cell sorting: DENV-specific CD8+ T cells were defined as tetramer+ CD3+ CD8+ lymphocytes. The number of sorted cells was recorded by index sorting for each sample and time point. Validation: In the independent validation cohort, DENV-specific CD8+ T cells were defined as tetramer+ CD3+ CD8+ lymphocytes. Within the naïve, TCM, TEM, and TEMRA subsets, the proportions of CX3CR1+ and CXCR6+ cells were quantified, expressed as percentages of the total tetramer+ CD8+ T cell pool. Tetramer specificity assessment: The proportions of serotype-specific and multiserotype cross-reactive CD8+ T cells were determined and presented as percentages of the total CD8+ T-cell pool.
Gating strategy	Cell sorting: Lymphocytes were first gated by FSC/SSC, followed by singlet discrimination using FSC-A/FSC-H. Live cells were identified using a viability dye, and CD8+ T cells were defined as CD3+CD8+. Tetramer+ CD8+ T cells were identified by PE/APC signal, with the cutoff set using non-tetramer–stained healthy controls. Validation: In the validation cohort, additional subset gating was performed using CD45RA, CD45RO, CCR7, CX3CR1, and CXCR6. Positive populations were defined using fluorescence minus one (FMO) controls generated from pooled PBMCs across all samples. Tetramer specificity assessment: After identification of live CD8+ T cells using a viability dye, tetramer+ cells were defined based on fluorescence above FMO controls. Serotype-specific populations were first determined, and multiserotype cross-reactive CD8+T cells were subsequently identified using Boolean gating.

- [x] Tick this box to confirm that a figure exemplifying the gating strategy is provided in the Supplementary Information.
