# Supplementary material for: Heterogeneity and dynamics of DENV-specific CD8 + T cells in dengue infection
Source: Nat Commun. 2026 Jun 3;17:7103. doi: 10.1038/s41467-026-73491-5 (PMC13392236; doi:10.1038/s41467-026-73491-5)
Supplement: Supplementary file 2 — Peer Review file [file 41467_2026_73491_MOESM2_ESM.pdf]

nature portfolio

Peer Review File

Heterogeneity and dynamics of DENV-specific CD8+ T cells in dengue infection

Corresponding Author: Dr Ponpan Matangkasombut

This file contains all reviewer reports in order by version, followed by all author rebuttals in order by version.

Version 0:

Reviewer comments:

Reviewer #1

(Remarks to the Author)
In this study, Srikor et al. examine DENV-specific CD8 T cells in individuals with asymptomatic, symptomatic, and severe dengue virus (DENV) infections. Using MHC tetramer-based sorting combined with plate-based single-cell RNA sequencing, the authors address key questions in dengue immunology, including: (1) the frequency of cross-reactive versus type-specific memory T cells during heterotypic secondary infections, and (2) the transcriptional profiles of DENV-specific CD8 T cells across different disease severities. The study leverages a unique sample set and robust analytical approach to address these knowledge gaps.

Major Concerns:

The authors use a single MHC/peptide tetramer probe per DENV serotype to identify virus-specific CD8 T cells, selecting HLA-compatible donors accordingly. However, the proportion of the total DENV-specific T cell response targeting this epitope remains unclear. If this epitope represents the dominant response, the findings would be highly compelling; however, if it captures only a minor fraction, the conclusions would be less generalizable. To contextualize these findings, it would be helpful to assess the total T cell response using conventional assays (e.g., ELISPOT) with both the selected peptides described in this manuscript as well as standard overlapping DENV peptide pools.

The authors use serology to infer participants' infection histories—an approach known to be challenging and potentially unreliable. The validity of this stratification method is unclear, as it appears unvalidated against samples from individuals with PCR-confirmed infection histories. To strengthen these findings, the authors should analyze T cell responses to DENV serotypes that participants presumably did not encounter (based on their inferred history). For instance, an individual with current DENV-2 infection and inferred past DENV-1 exposure should show minimal DENV-3 and DENV-4 specific T cells if the serological inference is accurate and/or informative.

The manuscript frequently cites "Sungnak et al. under revision" for foundational observations. While this manuscript was included with the submission, the current study's ability to stand independently is questionable given its reliance on unpublished findings critical to the analysis.

Minor Comments:

The introduction contains several imprecise statements requiring revision:

Mouse model limitations: References to mouse studies regarding T cell roles in dengue severity are problematic, as DENV neither naturally infects mice nor causes disease in immunocompetent animals. These citations do not adequately support the claims made.

CYD-TDV and ADE claim: The statement that "in dengue-naive children, CYD-TDV has been linked to an increased risk of hospitalization after primary infection due to ADE" is partially incorrect. While increased hospitalization risk is documented, no mechanistic evidence confirms ADE as the cause.

(Remarks on code availability)

Reviewer #2

(Remarks to the Author)
This manuscript presents an interesting and technically well-executed study investigating the heterogeneity of DENV-specific CD8⁺ T cell responses across different disease severity. Using plate-based single-cell RNA sequencing of sorted tetramer+ CD8 T cells reactive to both current and prior DENV serotypes, the authors identified distinct subsets characterized by differences in avidity, cytotoxic gene expression, and TCR repertoire. Integration of these data with PBMC and plasma cytokine profiles (from previous under review publication) revealed that clonally expanded CX3CR1⁺ CD8 T cells are enriched in severe disease and correlate with systemic inflammatory signatures. The study provides valuable insight into the complexity of DENV-specific T cell responses and their potential contribution to either protective or pathogenic immune outcomes. This is a well-written and thoughtfully designed manuscript with a clear biological question and solid technical execution. The conceptual framework linking antigen specificity, TCR avidity, and disease severity is strong and of potential relevance for vaccine development and immune monitoring. The figures are clearly and nicely presented, and the overall flow of the manuscript is good.
While the study is sophisticated, it remains largely descriptive and lacks the statistical robustness necessary to support its conclusions as a standalone paper. The major limitation is the small sample size, which prevents meaningful comparative analyses between disease groups. Most figures, including the flow cytometry (Fig. 1), single-cell clustering (Figure 2), TCR features (Fig. 4), and clonality analyses (Fig. 5), rely on descriptive visualization with insufficient statistical testing. As a result, although the trends are interesting, it is difficult to determine whether they reflect true biological differences or sampling variability. This limitation substantially weakens the impact and interpretability of the findings to a larger cohort of DENV+ patients. As presented, the dataset appears too narrow to represent the full spectrum of DENV-specific immune responses and the conclusions therefore remain speculative. Another concern is novelty relative to previous work. Similar datasets from the same or related groups have described phenotypic and transcriptional heterogeneity of DENV-specific T cells, including signatures of activation, exhaustion, and clonal expansion. This manuscript extends those findings on the tetramer+ CD8 T cells but does not introduce a sufficiently distinct mechanistic insight to justify a separate publication. The work would be more impactful if positioned as a focused report or as part of a broader cohort analysis integrating additional immunological parameters or larger cohort. Expanding the sample size, improving the statistical analyses, and including confirmatory experiments would significantly enhance the robustness and translational relevance of the study. Despite these limitations, the manuscript reflects careful experimental work and could form the foundation for a larger, more comprehensive investigation of DENV+-specific T cell responses during and after dengue infection.

(Remarks on code availability)

Reviewer #3

(Remarks to the Author)
The paper assesses an important question in the field, shedding light on the roles of DENV-specific CD8+ T cells in dengue disease pathogenesis. The cohort includes asymptomatic infected individuals, which provide unique insights into a protective immune response. In addition, the study focuses on the T cell response during acute (secondary) infection, which is unlike most studies focusing on the convalescent response. Even though the research question is relevant, and the cohort and methods used are unique and cutting edge, their remain important limitations with regard to sample size (both in terms of patients included and cells investigated), which limit the validity of the interpretation of the data.

Dengue disease is very dynamic, and changes in immune cell composition and activation occur rapidly. Patients are at risk to develop severe disease in a small window after onset of symptoms, usually 3-5 days post onset of symptoms. Here, it is unclear how many days after onset of symptoms the DF and DHF patients were included (this is a better measurement rather than days before defeverescence). In addition, the stage of infection in asymptomatic cases cannot be determined. This is an important limitation and should be mentioned in the paper. Maybe all ASD cases are earlier in their infection trajectory and therefore display less CX3CR1+ CD8+ T cells.

During secondary infection, usage of PRNT levels to evaluate previous infecting serotype should be done with caution, especially when only the acute phase timepoint is available. This should be clearly stated in the manuscript.

“biased reactivity to current versus previous serotypes”: the data (line 262-276) show a trend, not a statistically significant difference. Pls amend abstract and discussion where needed (eg. Line 61, line 440, etc)

How is the serotype specificity of the peptides evaluated? Can the authors be sure that the peptide-specific CD8+ T cells are specific for the current-previous infecting serotype? Data should be included of patients in the convalescent phase after RT-qPCR serotype confirmed primary infection, where serotype-specific reactivity of the used peptides is confirmed. In addition, tet staining should be performed on HC to evaluate specificity of the staining (Figure S2 shows the FMO for the HC, so there aspecific binding cannot be evaluated)

How sure are the authors that pHLA tetramer fluorescence intensity can be used as a proxy for TCR avidity? Is a higher MFI not associated with higher expression of TCR on the surface. The authors mention themselves somewhere that T cell

activation leads to downregulation of TCR and hence less pHLA+ cells. Do the authors have any data to support this interpretation?
In several instances, CD3CR1+ CD8+ T cells are called “potentially associated with vascular leakage in DHF”. What is the rationale for this?

On how many Tet+ CD8+ T cells were the scRNAseq analysis done for each individual? It would be good to add these numbers, as I suspect these are very small populations. Similary, on how many cells of each individual was the TCR repertoire analysis done? Was this not biased towards a few individuals with more cells?

Important recent publications in the field, such as Gregorova et al, Nat Comm 2025 and Gonnella et al, 2025 should be discussed.

(Remarks on code availability)

Version 1:

Reviewer comments:

Reviewer #1

(Remarks to the Author)
This submission has been significantly improved in revision and most of my prior suggestions/comments have been adequately addressed. However, there remains some points that need to be addressed.

While the authors have clearly justified their use of NS3-derived peptides for the identification and isolation of DENV-specific T cells, the point remains that it is unclear what fraction of the total DENV-specific T cell response this population represents. Even in the papers cited by the authors to justify their exclusive use of these NS3-derived peptides for this analysis, significant responses to antigens other than NS3 are observed, with the difference between the subdominant and immunodomant antigens differing only by 1-2 fold. The additional bioinformatic analysis provided in the rebuttal doesn't directly alleviate this concern, as up to 98% of expanded/activated T cell clones do not appear to express GTS-specific TCRs. I understand the limitations and challenges associated with this analysis approach - and wholeheartedly agree with the authors that this is the best approach given the limitations of other assays - but I would appreciate the authors being more precise in how the data are framed/presented. If the authors aren't able to clearly define what fraction of the total DENV-specific T cell responses these TET-positive CD8 T cells represent, I would recommend that the conclusions and data clearly state "DENV NS3 specific" or "GTS-specific" T cell responses rather than "DENV-specific T cell responses.

There remains some cognitive dissonance regarding the relationship between antibody titers, CD8 T cell responses, and inferred infection history. The authors maintain that one of the primary objectives of this analysis is to compare the T cell responses directed against historic and current DENV antigens, with the historic virus inferred by serology. However, in their rebuttal, the authors acknowledge that "in some donors we observed relatively low PRNT titers to certain serotypes despite detectable Tet+ CD8+ T-cell responses. This further supports the concept that neutralizing antibody and CD8+ T-cell responses do not necessarily correlate perfectly and may reflect differential or biased immune recall within distinct immune compartments". Given this discordance, why is prior infection history pinned to the specificity of the antibody responses rather than the T cell response? In light of this - and the clearly acknowledged challenges associated with inferring infection history based on serology - I would strongly recommend paraphrasing/repositioning "inferred prior infection" or "previous infection" to something along the limes of "Predominant seroreactivity" or "putative serologic exposure". Acknowledging these limitations in no way detracts from the importance or impact of the observations described in this study, but more precisely reflects the data.

(Remarks on code availability)

Reviewer #2

(Remarks to the Author)
I appreciate the effort the authors have made in revising the manuscript and responding to the previous comments.

The figures are clearer, and some additional statistical analyses have been added. That said, several major concerns remain. First, the cohort is still small and heterogeneous, which limits statistical power, and many interpretations feel overextended relative to the data. My primary concern—that most figures remain largely descriptive with insufficient statistical evaluation persists. I recognize that sampling from this patient population is challenging, but the main conclusions of the manuscript rely on observations that are not fully corroborated. Claims regarding TCR avidity and CX3CR1 enrichment continue to depend on indirect measures, such as tetramer MFI proxies, without any direct protein-level or functional validation. Likewise, the functional significance of the observed phenotypes is not completely demonstrated, and although the limitations of ELISPOT are acknowledged, they remain unaddressed.

Overall, while the manuscript is interesting, it doesnt provide strong enough evidence to fully support its conclusions in its

current form.

(Remarks on code availability)

Reviewer #3

(Remarks to the Author)
I thank the reviewers for their careful replies to the comments and concerns of the reviewers. However, concerns remain:
1. Sample size: the number of individuals included is limited. The identification of AD is challenging, however, more hospitalized patients could be included to increase robustness of the findings
2. Amount of cells analyzed. This information is new and is now provided by the authors (Table S3). For more than half of the individuals, less than 100 cells were analyzed. This raises substantial concerns on the robustness of the findings.
3. Concerns on the serotype-specificity of the tetramers used. The authors did not validate their tetramers in convalescent primary cases, as requested by me and another reviewer, so the extend of cross-reactivity remains unknown.
4. The limitation on knowledge on previous infecting serotype. Point 3 and 4 together make that the claims “a trend of biased reactivity to the infecting serotype” and the designation “serotype specific” and “cross reactive” CD8+ T cells highly uncertain and not supported by sufficient controls and is highly speculative. All analysis regarding this topic should be omitted from the manuscript.

(Remarks on code availability)

Open Access This Peer Review File is licensed under a Creative Commons Attribution 4.0 International License, which permits use, sharing, adaptation, distribution and reproduction in any medium or format, as long as you give appropriate credit to the original author(s) and the source, provide a link to the Creative Commons license, and indicate if changes were made.
In cases where reviewers are anonymous, credit should be given to 'Anonymous Referee' and the source.
The images or other third party material in this Peer Review File are included in the article’s Creative Commons license, unless indicated otherwise in a credit line to the material. If material is not included in the article’s Creative Commons license and your intended use is not permitted by statutory regulation or exceeds the permitted use, you will need to obtain permission directly from the copyright holder.
To view a copy of this license, visit https://creativecommons.org/licenses/by/4.0/

Response to Reviews

Reviewer #1(Remarks to the Author):
In this study, Srikor et al. examine DENV-specific CD8 T cells in individuals with asymptomatic, symptomatic, and severe dengue virus (DENV) infections. Using MHC tetramer-based sorting combined with plate-based single-cell RNA sequencing, the authors address key questions in dengue immunology, including: (1) the frequency of cross-reactive versus type-specific memory T cells during heterotypic secondary infections, and (2) the transcriptional profiles of DENV-specific CD8 T cells across different disease severities. The study leverages a unique sample set and robust analytical approach to address these knowledge gaps.

We appreciate the reviewer for the constructive evaluation of our study and for noting the unique sample set and robust analytical approach used to address key questions in dengue immunology.

Major Concerns:

1. The authors use a single MHC/peptide tetramer probe per DENV serotype to identify virus-specific CD8 T cells, selecting HLA-compatible donors accordingly. However, the proportion of the total DENV-specific T cell response targeting this epitope remains unclear. If this epitope represents the dominant response, the findings would be highly compelling; however, if it captures only a minor fraction, the conclusions would be less generalizable. To contextualize these findings, it would be helpful to assess the total T cell response using conventional assays (e.g., ELISPOT) with both the selected peptides described in this manuscript as well as standard overlapping DENV peptide pools.

We thank the reviewer for raising this important point regarding the dominance of the DENV-specific CD8+ T cell responses captured by the selected epitopes. We would like to address the reviewer's concern by first summarizing prior literature supporting the immunodominance of these particular set of epitopes used in this study, and then presenting additional quantitative evidence from our dataset confirming that they represent substantial fractions of the acute DENV-specific CD8+ T cell responses.

In this manuscript, we focused on previously characterized CD8+ T cell epitopes: HLA-A*11-restricted GTS epitopes and HLA-A*24-restricted NYA epitopes, both derived from the DENV NS3 protein, which has been consistently identified as a major target of antiviral CD8+ T cell responses in humans[1,2]. For the GTS epitopes, their immunodominance has been consistently demonstrated across multiple independent cohorts and experimental platforms[2-9]. Across studies, tetramer-defined GTS-specific CD8+ T cells have frequently been reported to comprise approximately 0.1-10% of total circulating CD8+ T cells during convalescence[2-3,5-9]. Notably, among HLA-A*11-restricted epitopes, the GTS epitope is detected in a larger number of

responding individuals and at higher frequencies than other tested epitopes, indicating both a high responder rate and preferential epitope usage within the HLA-A*11 context[9].

In addition, our data further provides quantitative support that this epitope represents a major contributor to the CD8+ T cell response during acute dengue infection. Specifically, we inferred putative GTS-specific CD8+ T cells within the total CD8+ T-cell compartment based on amino acid-level TCR sequence similarity to pHLA-A*11-sorted CD8+ T cells using clonotype network analysis, enabling donor-level estimation of their relative contribution to circulating CD8+ T cells. The analysis revealed that inferred GTS-specific CD8+ T cells were detected in nearly all HLA-matched donors (9 out of 10) during acute dengue infection and reached high frequencies, accounting for approximately 0.2-30% of total circulating CD8+ T cells (Rebuttal Figure 2). One donor (Y016) did not show detectable matched GTS-specific cells during the acute phase, where only a single cell was sorted. However, 184 Tet+ CD8+ T cells were detected during the convalescent phase. This indicates that all 10 of 10 HLA-A*11+ donors mounted a measurable GTS-specific CD8+ T cell response across the infection course. This is in line with other defined immunodominant epitopes in other viral infection contexts. For example, in SARS-CoV-2 infection, the widely studied HLA-B*07:02-restricted $NP_{105-113}$ (SPRWYFYYL) epitope reaches approximately ~0.01-9% of total CD8+ T cells[10-12] , whereas in influenza infection, classic immunodominant epitopes such as $A68\text{-}NP_{145}$, $A2\text{-}M1_{58}$ and $A2\text{-}PB1_{413}$ typically account for ~0.001-0.02%, ~0.001-1% and ~0.001-0.05% of CD8+ T cells, respectively[13,14], providing a contextual benchmark that confirms the rationale in defining the immunodominance of our selected epitopes.

We have addressed this point in the Results section and incorporated Rebuttal Figure 2 into the revised manuscript as Supplementary Figure 11c.

Original Text (Result):
Line 363: Through this integration, a high proportion of GTS-specific CD8+ T cells was identified within the total CD8+ T cell population.

Revised Text (Result):
Line 360: Through this integration, inferred GTS-specific CD8+ T cells were identified within the total CD8+ T cell population in the 10X scRNA-seq data, accounting for approximately 0.2–30% across donors (Supplementary Fig. 11c), consistent with the established immunodominance of these epitopes in HLA-A*11-restricted dengue responses[11-13,17,25,26]

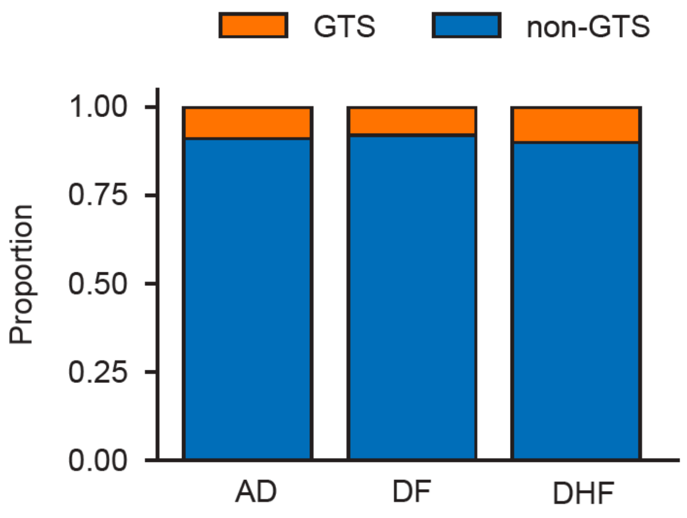

Rebuttal Figure 1. Proportion of inferred GTS-specific CD8+ T cells relative to non–GTS-specific CD8+ T cells across disease severity groups in HLA-A*11+ donors (corresponding to Figure 5C in the revised manuscript).

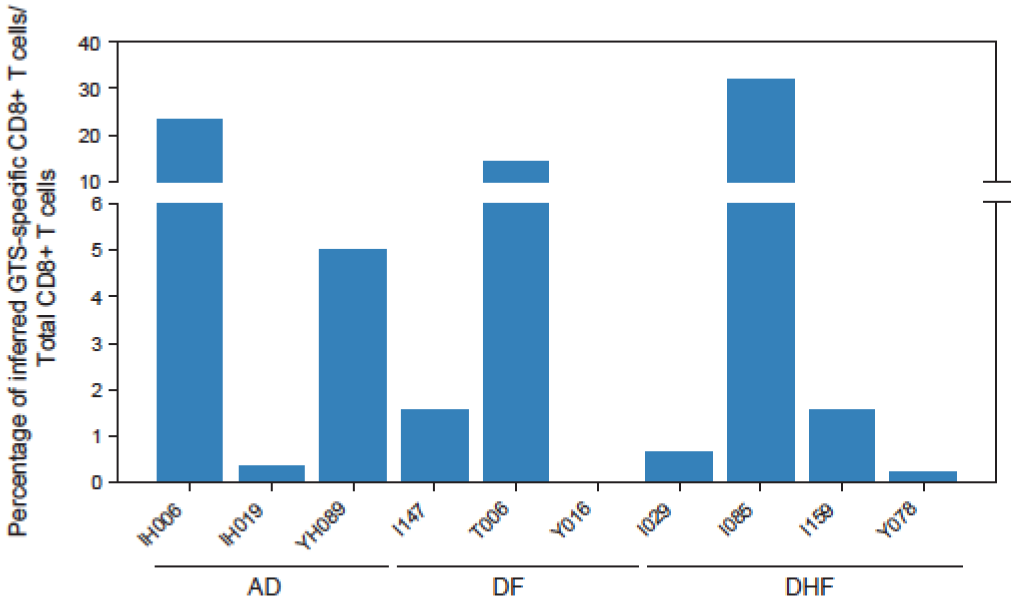

Rebuttal Figure 2. Percentage of inferred GTS-specific CD8+ T cells among total CD8+ T cells in the 10X scRNA-seq dataset, stratified by HLA-A*11+ donors.

Furthermore, when focusing specifically on clonally expanded CD8+ T cells, used here as a proxy for antigen-experienced and recently activated populations, while acknowledging that

bystander activation cannot be fully excluded, GTS-specific cells comprised approximately 1.5-55% of expanded clones, representing even higher proportions than those observed within the total CD8+ T cell pool (Rebuttal Figure 3). The frequencies are consistent with our independent validation cohort of HLA-A*11+ acute dengue cases, in which GTS Tet+ cells reached up to 0.5-9% of CD8 TEM and TEMRA cells, with a single outlier reaching 16% (Rebuttal Figure 4). Taken together, such enrichments within the clonally expanded compartment further support immunodominance of this epitope among potential responding effector CD8+ T cells during acute dengue infection.

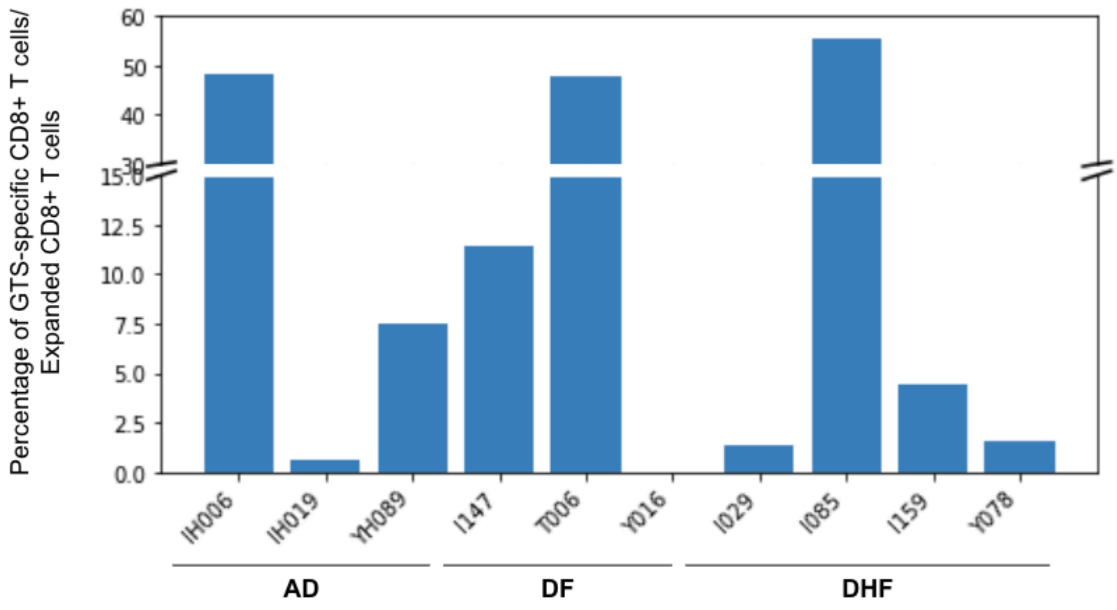

Rebuttal Figure 3. Percentage of inferred GTS-specific CD8+ T cells among expanded CD8+ T cells in the 10X scRNA-seq dataset, stratified by HLA-A*11+ donors.

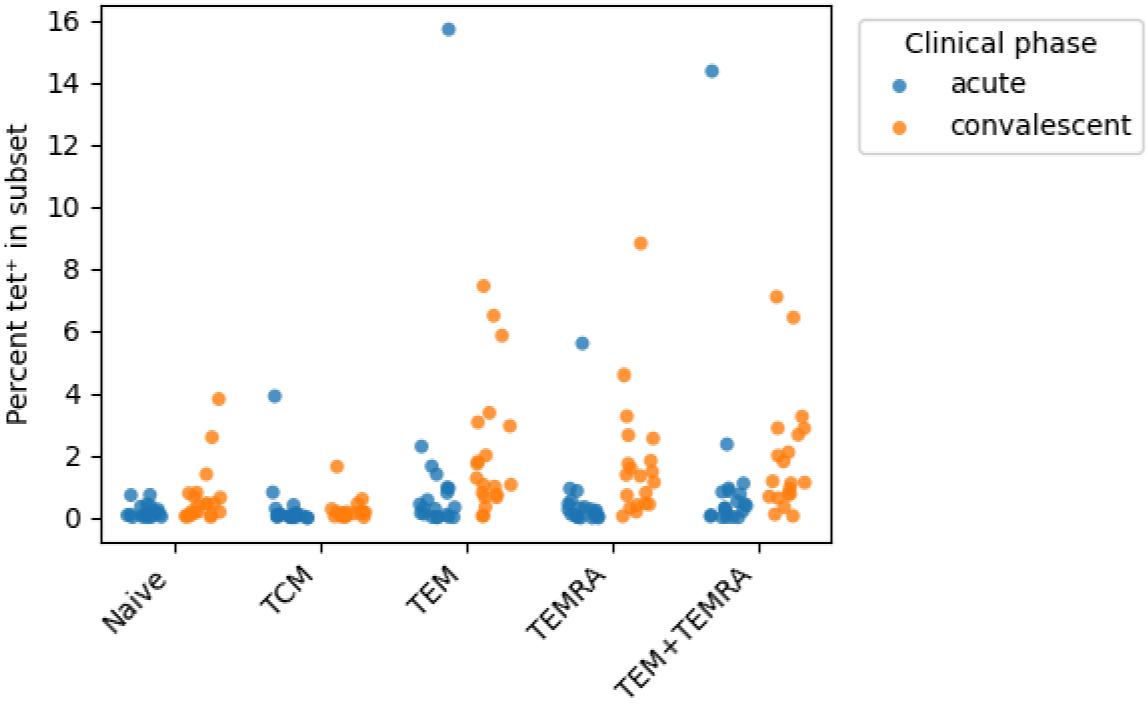

Rebuttal Figure 4. Percentage of Tet+ CD8+ in different CD8 T-cell subsets among total CD8+ T cells in the independent flow cytometry validation cohort (HLA-A*11)

For the other selected epitope, the NS3-derived NYA one is restricted by HLA-A*24. HLA-A*24 is also prevalent in Thailand and across Southeast Asia (~20%)[15], making this epitope particularly relevant for expanding the analysis of epitope-specific TCR diversity beyond HLA-A*11-restricted responses.

Although less extensively characterized than GTS, the NYA epitope has been reported as an immunodominant NS3-derived epitope in both human and experimental models[16,17]. NYA-specific CD8+ T-cell responses have been detected in murine systems using ELISPOT assays[17], and in human studies the NS3-derived peptide NYADRRWCF (NYA) has been recognized by CD8+ T cells from DHF patients, with responses ranging from approximately 40–256 spot-forming cells per 10^6 PBMCs across donors[18]. Acute CD8+ T-cell responses to natural NS3 variants, including NYA, have also been observed in individuals with secondary dengue infection[19]. Functional analyses further suggest that NYA-specific CD8+ T cells derived from prior infections may exhibit suboptimal responses compared with T cells specific for the currently infecting serotype[20]. Notably, the NYA epitope is relatively conserved across DENV serotypes and shows sequence similarity within the flavivirus family, including Zika virus, supporting its potential role in cross-reactive CD8+ T-cell immunity[21]. Collectively, these findings establish NYA as a well-described and biologically relevant NS3 immunodominant epitope in human dengue infection and related flaviviruses, warranting us to select it for our investigation as well.

In our study, we successfully sorted NYA-HLA-A*24 Tet+ CD8+ T cells. However, the number of productive TCR sequences recovered was limited, thereby reducing statistical power and increasing the risk of over-interpretation. Accordingly, although NYA-specific cells were included in the experimental framework, the TCR repertoire analyses focused primarily on GTS-specific CD8+ T cells, for which sequencing depth and clonotype representation were sufficient to support robust and statistically sound conclusions. Therefore, the principal conclusions of this work are mainly derived from GTS-specific responses, and we have clearly indicated throughout the manuscript which findings are epitope-specific and which are based on combined analyses.

In summary, the epitopes selected in this study should capture immunodominant components of the DENV-specific CD8+ T cell response in HLA-matched individuals and are expected to be sufficient to interrogate, at least partially, disease-associated heterogeneity within antigen-specific CD8+ T cells. Nonetheless, we acknowledge that additional epitopes may contribute to the broader CD8+ T cell repertoire, and that a more systematic expansion of epitope coverage and HLA alleles warrants further investigation. However, such questions are beyond the scope of the present study.

We have revised the manuscript and incorporated this point as a future perspective in the Discussion section (Line 544)

"Further studies with broader pHLA and larger sample size, and more efficient antigen-specific T-cell capture techniques are warranted"

Regarding the reviewer's suggestion to use conventional assays such as ELISPOT with individual peptides and overlapping peptide pools to contextualize epitope dominance, we agree in principle that the immunodominance of the selected epitopes should be carefully assessed. As detailed above, evidence from the literature, along with the quantitative observations in our dataset, supports that the selected epitopes, particularly the HLA-A*11-restricted GTS ones, are major contributors to the acute DENV-specific CD8+ T cell response in HLA-matched individuals. However, ELISPOT assays performed during the acute febrile phase of dengue infection, which is the primary focus of our study, have well-characterized limitations. Prior studies have demonstrated that during acute dengue (D-1, D0), activated CD8+ T cells often exhibit reduced increased activation-induced cell death, which may lead to transient functional impairment and consequently diminished ELISPOT readouts[3,20] (Rebuttal Figure 5-6) . Therefore, ELISPOT results during acute infection may underestimate the true magnitude of antigen-specific CD8+ T cell responses. While ELISPOT assays performed during convalescence could partially mitigate these constraints, convalescent measurements would not directly reflect acute-phase immunodominance or the acute-phase clonal and phenotypic states that are central to the objectives of the present study, particularly given the marked differences we observe between acute and convalescent phases in both clonal composition and subset distribution.

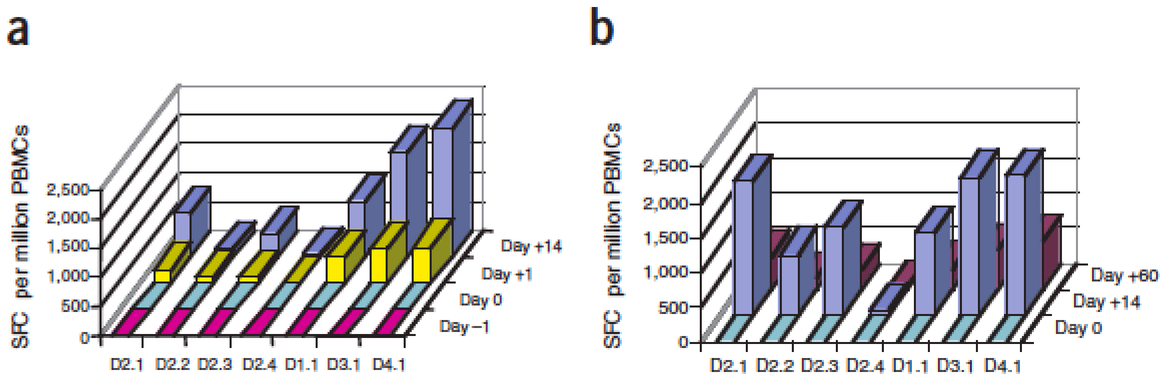

Rebuttal Figure 5. Longitudinal IFN-γ ELISPOT responses to GTS peptide variants across DENV serotypes at multiple time points during acute, post-febrile and convalescent infection (Mongkolsapaya et al., 2003).

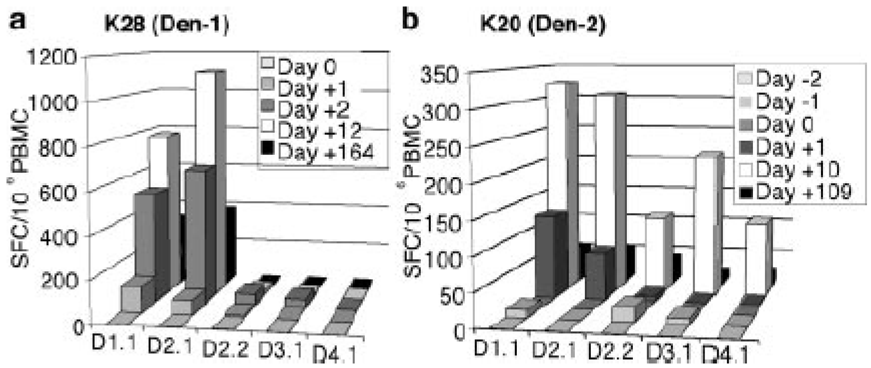

Rebuttal Figure 6. Longitudinal IFN-γ ELISPOT responses to NYA peptide variants across DENV serotypes at multiple time points during acute, post-febrile and convalescent infection (Mongkolsapaya et al., 2006).

2. The authors use serology to infer participants' infection histories—an approach known to be challenging and potentially unreliable. The validity of this stratification method is unclear, as it appears unvalidated against samples from individuals with PCR-confirmed infection histories.

We agree with the reviewer that inferring dengue infection history using serological assays is inherently imperfect. However, it is important to note that serology-based frameworks, particularly those incorporating neutralization assays such as PRNT or FRNT, have nonetheless formed the foundation of much of our current understanding of dengue epidemiology, immune imprinting, and T cell immunology. These approaches have been extensively used in landmark surveillance, vaccine, and immunological studies to distinguish primary versus secondary infections and to contextualize immune responses in the absence of definitive virological documentation of prior infections[22-26].

We also agree in principle that validation using individuals with PCR-confirmed infection histories would represent an ideal reference. In practice, however, virologically confirmed documentation of historical infecting serotypes is available only in a very limited number of long-running prospective cohorts or hospital-based studies, in which the same individual is captured and serotyped during multiple sequential symptomatic infection episodes[27] . Even in these highly specialized settings, asymptomatic or subclinical infections, which constitute a substantial proportion of dengue infections, remain difficult to capture systematically[28] .

In our study context, reconstructing infection history using PCR-confirmed prior infections would be particularly challenging. As dengue transmission was endemic in our settings, prior infections were frequently asymptomatic or unrecognized, and our analyses further required stratification by specific HLA alleles. As a result, identifying individuals with PCR-confirmed historical infection histories who also meet these immunogenetic and clinical criteria would require extremely large-scale, long-term surveillance and would not be practically compatible with the acquisition of rare asymptomatic dengue cases, even with a five-year active surveillance effort done in this study.

In this manuscript, while still using a serology-based framework to infer infection history, we attempted to strengthen it by integrating multiple orthogonal measurements, including hemagglutination inhibition titers, IgM/IgG ratios, qPCR-confirmed current infecting serotype, and paired PRNT measurements obtained during the acute and convalescent phases (Rebuttal Table 1). This integrated approach reduces reliance on any single assay and provides a biologically informed means to contextualize infection history in the absence of virologically confirmed prior infections.

Rebuttal Table 1 Participant demographics, clinical parameters and applied single-cell RNA-seq techniques (corresponding to Supplementary Table 1 in revised manuscript)

Table S1: Participant demographics, clinical parameters and applied single-cell RNA-seq techniques

Sample ID	ID	HLA	Severity	Days post-onset of symptoms (DPO)	Serotype	Age	Sex	Viral copies/ml (qRT-PCR)	Primary/ Secondary infection	Ig Titer(HI)								scRNA-seq technique
									HI	Timepoint	DENV1	DENV2	DENV3	DENV4	JEV	Zika	Chik	
IH006	1	A*11	AD	N/A	DENV4	28	F	1.03E+07	Secondary	D1	N/A	N/A	N/A	N/A	N/A	N/A	N/A	SS2,10X
IH019	2	A*11	AD	N/A	DENV3	63	M	1.64E+06	Secondary	D1	N/A	N/A	N/A	N/A	N/A	N/A	N/A	SS2,10X
IH023	3	A*24	AD	N/A	DENV3	25	M	1.17E+04	Secondary	D1	N/A	N/A	N/A	N/A	N/A	N/A	N/A	SS2,10X
YH045	4	A*02	AD	N/A	DENV4	19	M	2.19E+06	Unknown	D1	N/A	N/A	N/A	N/A	N/A	N/A	N/A	10X
YH049	5	A*11	AD	N/A	DENV4	34	F	5.37E+06	Secondary	D1	N/A	N/A	N/A	N/A	N/A	N/A	N/A	10X
YH089	6	A*11	AD	N/A	DENV1	38	F	1.06E+03	Primary	D1	N/A	N/A	N/A	N/A	N/A	N/A	N/A	SS2,10X
H001	7	A*02	AD	N/A	DENV3,4	18	F	3.60E+04,<br>1.47E+07	Secondary	D1	N/A	N/A	N/A	N/A	N/A	N/A	N/A	10X
H030	8	A*02	AD	N/A	DENV3	39	M	5.31E+04	Secondary	D1	N/A	N/A	N/A	N/A	N/A	N/A	N/A	10X
I160	9	A*02	DF	3	DENV3	39	M	2.76E+04	Secondary	D1	N/A	10240	10240	N/A	5120	N/A	0	10X
T010	10	A*24	DF	5	DENV3	22	F	5.34E+03	Secondary	D1	N/A	5120	5120	N/A	10240	2560	0	SS2,10X
T006	11	A*11	DF	4	DENV4	15	F	1.77E+06	Secondary	D1	N/A	5120	20480	N/A	5120	N/A	0	SS2,10X
Y029	12	A*24	DF	3	DENV4	60	F	7.04E+06	Secondary	D1	N/A	10240	10240	N/A	20480	10240	320	SS2,10X
Y016	13	A*24 & A*11	DF	N/A	DENV4	46	F	6.99E+08	Secondary	D1	N/A	160	320	N/A	640	160	0	SS2,10X
I147	14	A*11	DF	5	DENV3	18	M	1.96E+07	Secondary	D1	N/A	5120	25560	N/A	2560	N/A	0	SS2,10X
I171	15	A*24	DF	3	DENV4	65	F	2.65E+06	Secondary	D1	N/A	5120	20480	N/A	5120	1280	0	SS2,10X
I109	16	A*24	DF	5	DENV1	38	M	3.52E+02	Secondary	D1	N/A	10240	20480	N/A	10240	N/A	0	SS2,10X
I085	17	A*11	DHF	3	DENV3	17	M	6.87E+03	Secondary	D1	N/A	10240	1520	N/A	5120	N/A	0	SS2,10X
Y078	18	A*24 & A*11	DHF	4	DENV3	23	F	3.35E+04	Secondary	D1	N/A	1280	2560	N/A	5120	1280	0	SS2,10X
I010	19	A*02	DHF	4	DENV3	26	M	2.03E+07	Secondary	D1	N/A	1280	1280	N/A	1280	N/A	0	10X
I016	20	A*02	DHF	5	DENV4	34	F	5.79E+06	Secondary	D1	N/A	20480	20480	N/A	10240	N/A	80	10X
I159	21	A*11	DHF	3	DENV4	38	M	1.41E+07	Secondary	D1	N/A	80	80	N/A	40	N/A	0	SS2,10X
I024	22	A*02	DHF	5	DENV4	33	F	8.61E+05	Secondary	D1	N/A	5120	5120	N/A	1280	N/A	0	10X
I029	23	A*11	DHF	5	DENV4	20	F	1.66E+06	Secondary	D1	N/A	20480	5120	N/A	2560	N/A	0	SS2,10X
I075	24	A*24	DHF	5	DENV1	15	F	7.54E+06	Secondary	D1	N/A	10240	20480	N/A	5120	N/A	80	SS2,10X

N/A: Data is not available

In response to the reviewer's concern, we have revised the manuscript to acknowledge the limitations of our serology-based infection history inference, clarify the definition of serologically

inferred previously infecting serotypes, and include an example illustrating the potential for misclassification. These points have been added to the Discussion.

Original Text (Discussion):
Line 566: Additionally, although primary or secondary infection status was generally determined using antibody-based assays such as PRNT or HI[65], we found one AD case previously classified as primary infection based on antibody assay may actually represent a secondary infection, as indicated by DENV-specific T cell responses to previously encountered serotypes. This observation highlights a potential limitation of antibody-based classification in AD, which is characterized by predominant T cell response and low antibody one.

Revised Text (Discussion):
Line 555: Another limitation relates to the determination of infection history. Primary versus secondary infection status was determined using antibody-based assays such as PRNT or hemagglutination inhibition (HI) assay[65], which may not perfectly reconstruct historical infecting serotypes, particularly in endemic settings. To reflect this limitation, the relevant serotypes assigned to these samples are described as serologically inferred dominant previously infecting serotypes, defined operationally based on neutralization titers rather than definitive virological confirmation historically. Consistent with this limitation, we identified one AD case initially classified as primary based on serology that may instead represent a secondary infection, as suggested by detectable DENV-specific T-cell responses to inferred previously encountered serotypes. This observation highlights a potential limitation of antibody-based determination of previously infecting serotypes, particularly in AD, which is characterized by substantial T cell responses and relatively lower antibody ones[18].

While more definitive approaches to reconstructing infection history, particularly for asymptomatic infections, would be valuable and may further refine such classifications in the future, the serological stratification strategy used here aligns with current standard practices in the field and provides a practical and biologically meaningful framework to characterize antigen-specific CD8+ T cell responses in relation to dengue disease severity that includes asymptomatic cases.

3. To strengthen these findings, the authors should analyze T cell responses to DENV serotypes that participants presumably did not encounter (based on their inferred history). For instance, an individual with current DENV-2 infection and inferred past DENV-1 exposure should show minimal DENV-3 and DENV-4 specific T cells if the serological inference is accurate and/or informative.

We appreciate the reviewer for this suggestion. In this study, the current infecting serotype was determined by qRT-PCR. The previously infecting serotype was then inferred from PRNT results based on the highest neutralizing antibody titer among the remaining serotypes, excluding the current infecting serotype. Both approaches represent standard methodologies widely used in

dengue research[3,8,20,24,26]. It should be noted that in hyperendemic areas, multiple previous infections are common and cross-reactivity between serotypes also exists [29,30].

To more comprehensively evaluate the landscape of CD8+ T-cell specificity across DENV serotypes, we performed additional staining using epitope-defined pHLA tetramers representing all four DENV serotypes, including GTS-restricted (HLA-A*11) and NYA-restricted (HLA-A*24) epitopes. Each tetramer was labeled with a distinct fluorochrome to enable simultaneous detection. Because some D-1 samples used in the original analyses were depleted, these experiments were performed using samples collected at defervescence (D0), for which material was available from all donors included in the original experiments.

As Tet+ CD8+ T cells were present at low frequencies during the acute phase (as shown in Supplementary Figure 2), we applied PKI treatment to enhance TCR surface expression and improve the frequency of Tet+ populations[36].

Using this approach, we observed both serotype-specific and cross-reactive CD8+ T-cell populations across multiple DENV serotypes, including responses to serotypes not inferred as prior infections by PRNT or HI (Supplementary Table. 2). Notably, PRNT frequently detected neutralizing antibodies against multiple serotypes in several donors (Rebuttal Table 2-3). These patterns likely reflect complex exposure histories, including multiple sequential infections, which are common in highly endemic settings[29,30]. In such contexts, individuals may accumulate heterogeneous antibody titers across serotypes, while simultaneously developing diverse and cross-reactive CD8+ T-cell repertoires. Therefore, CD8+ T-cell responses to serotypes not inferred as the dominant prior infection by PRNT are biologically plausible and expected.

Additionally, in some donors we observed relatively low PRNT titers to certain serotypes despite detectable Tet+ CD8+ T-cell responses. This further supports the concept that neutralizing antibody and CD8+ T-cell responses do not necessarily correlate perfectly and may reflect differential or biased immune recall within distinct immune compartments[31]. These points have been added to the Discussion.

Added Text (Discussion):
Line 564: In addition, serotype-specific neutralizing antibody and CD8+ T-cell responses do not necessarily correlate directly (Supplementary Table 7,8) and may reflect differential or biased immune recall within distinct immune compartments.

To address the reviewer’s concern, we have revised the terminology from “previously infecting serotype” to “serologically inferred dominant previously infecting serotype” to more accurately reflect the serology-based inference used in this study. Specifically, the serologically inferred dominant previously infecting serotype was defined as the non-current DENV serotype with the highest PRNT titer during the acute phase. The PCR-confirmed infecting serotype during the acute episode was still designated as the current serotype, even if it exhibited the highest PRNT titer. This classification reflects the dominant serological imprint excluding the ongoing infection and does not imply definitive virological confirmation of historical infection. This definition has been explicitly stated in the revised manuscript for better clarity.

Original Text (Result):
Line 162: …previously infecting DENV serotypes, as determined via qPCR and plaque reduction neutralization test (PRNT) assays, respectively.

Revised Text (Result):
Line 159: …serologically inferred dominant previously infecting DENV serotypes. In this study, the currently infecting serotype was determined by qPCR, while the serologically inferred dominant previously infecting serotype was defined as the non-current serotype with the highest plaque reduction neutralization test (PRNT) titer (SupplementaryTable 2).

Original Text (Result):
Line 342: …, TCRs targeted serotypes from previous infections, regardless of CD8+ T cell subset (Fig. 5a)

Revised Text (Result):
Line 342: …, TCRs preferentially targeted serologically inferred dominant previously infecting serotypes, regardless of CD8+ T cell subset (Fig. 5a).

Added Texted (Abstract):
Line 62: serologically inferred dominant previous DENV serotypes…

Added Text (Result):
Line 180: …serologically inferred dominant previous serotype
Line 282: …serologically inferred dominant previously infecting serotypes

Added Text (Discussion):
Line 460: …serologically inferred dominant previously infecting serotypes

Added Text (Method):
Line 630: We selected serologically inferred dominant previously infecting serotypes as the non-current DENV serotype with the highest PRNT titer in acute phase.

Rebuttal Table 2: PRNT50 titers against DENV1–4 and distribution of serotype-specific and cross-reactive GTS-specific CD8+ T cells across HLA-A*11 donors

Donor_ID	Severity	PRNT50 titer				
		Time-point	DENV1	DENV2	DENV3	DENV4
I147	DF	D1	2108	13286	2960	2555
		F1	6350	372805	26783	61984
T006	DF	D1	42206	30915	17840	16113
		F1	308937	129567	45104	37607
Y016	DF	D1	N/A	N/A	N/A	N/A
		F1	N/A	N/A	N/A	N/A
I085	DHF	D1	25703	64353	3004	14401
		F1	6365	136271	4443	41112
I159	DHF	D1	9	315	48	40
		F1	7127	51718	4797	25201
Y078	DHF	D1	1558	4923	1582	6366
		F1	N/A	N/A	N/A	N/A
I029	DHF	D1	6148	11956	6739	4291
		F1	N/A	N/A	N/A	N/A

High

Low

Current
Inferred previous

Sample_ID	Percent of CD8 T cells					
	Serotype-specific			Cross-reactive		
	Tet-GTS1	Tet-GTS2	Tet-GTS3/4	Tet-GTS1|2	Tet-GTS1|3/4	Tet-GTS2|3/4
I147D0	0.387	0.035	0.228	0.121	0.314	0.125
T006D0	0.65	0.225	1.397	1.158	1.437	0.467
Y016D0	0.71	0.098	0.177	0.095	0.212	0.084
I085D0	0.215	0.224	0.753	0.633	1.272	0.615
I159D0	1.74	0.185	0.188	0.065	0.105	0.062
Y078D0	0.316	0.066	0.199	0.036	0.081	0.01
I029D0	0.169	0.041	0.813	0.503	1.152	0.498

Rebuttal Table 3: PRNT50 titers against DENV1–4 and distribution of serotype-specific and cross-reactive NYA-specific CD8+ T cells across HLA-A*24 donors

Donor_ID	Severity	PRNT50 titer				
		Time-point	DENV1	DENV2	DENV3	DENV4
IH023	AD	D1	404	1713	1399	1254
		F1	N/A	N/A	N/A	N/A
T010	DF	D1	3590	7424	1652	54194
		F1	5944	16706	7618	38598
Y029	DF	D1	748	43559	3928	4632
		F1	41244	20879	28983	38806
I109	DF	D1	4947	3646	33863	23419
		F1	8439	14483	26914	45461
I171	DF	D1	8140	20770	27268	13709
		F1	42881	29865	38709	86973
I075	DHF	D1	3423	41067	4533	340
		F1	19546	17044	142196	26523

High

Low

Current
Inferred previous

Sample_ID	Percent of CD8 T cells									
	Serotype-specific				Cross-reactive					
	Tet-NYA1+	Tet-NYA2+	Tet-NYA3+	Tet-NYA4+	Tet-NYA1|2	Tet-NYA1|3	Tet-NYA1|4	Tet-NYA2|3	Tet-NYA2|4	Tet-NYA3|4
IH023	0.011	0.359	0.378	0.452	0.005	0.005	0.005	0.183	0.21	0.279
T010D0	0	0.234	0.315	3.098	0.019	0.019	0.017	2.895	3.358	3.342
Y029D0	0	0.185	0.539	2.797	0.002	0.002	0.002	0.487	0.613	0.913
I109D0	0	0.211	0.369	3.121	0.018	0.019	0.019	2.448	2.994	2.8
I171D0	0.012	2.08	0.844	1.024	0.009	0.009	0.009	3.696	0.691	0.685
I075D0	0	0.179	0.28	1.224	0.016	0.006	0.003	0.58	0.726	0.721

We have also added the tetramer binding landscape analysis for the four dengue serotypes to the Methods section. Rebuttal Tables 2-3 have been incorporated into the revised manuscript as Supplementary Tables 7-8.

Added Text (Method):

Line 726: To assess tetramer specificity across the four DENV serotypes, PBMC samples collected at defervescence (D0) were first stained with LIVE/DEAD Fixable Green Dead Cell Stain and treated with dasatinib prior to tetramer staining. Cells were then incubated with serotype-specific HLA tetramers labeled with PE, APC, BV421, and PE-Cy7. Tet+ CD8+ T cells were identified based on tetramer fluorescence above FMO controls. Serotype-specific and cross-reactive CD8+ T cells were determined using Boolean gating in FlowJo™ v10.10 software (Supplementary Table 7-8)

4. The manuscript frequently cites "Sungnak et al. under revision" for foundational observations. While this manuscript was included with the submission, the current study's ability to stand independently is questionable given its reliance on unpublished findings critical to the analysis.

We thank the reviewer for raising this point so we could have an opportunity to clarify the relationship between the present study and *Sungnak et al.*, which has now been published in *Science Translational Medicine* (DOI: 10.1126/scitranslmed.ads5932)[32]. While insights from our broader research program informed the scientific context of this work, the present manuscript was designed, executed, and analyzed as a distinct follow-up project and is conceptually, experimentally, and analytically independent, with its own primary biological questions and experimental strategy.

Sungnak et al. focuses on global PBMC-level immune dynamics during dengue infection, characterizing broad changes in immune cell composition and patterns of total CD8+ T cell clonal expansion across disease severities. In contrast, the current manuscript directly examines the temporal dynamics of dengue-specific CD8+ T cells identified using epitope-defined pHLA tetramers and provides a high-resolution dissection of their heterogeneity by integrating (i) antigen specificity, (ii) TCR gene usage and clonotype structure, and (iii) functional phenotypes across clinical severity groups.

Importantly, the primary analyses and our conclusions of the present study rely on experimental readouts unique to this work, including quantitative tetramer fluorescence intensity as a proxy for pHLA-TCR binding strength, enabling direct linkage between antigen reactivity, binding strength, and CD8+ T cell phenotypes. The resulting biological insights, particularly regarding heterogeneity within antigen-specific CD8+ T cells and their association with disease outcome, are supported directly by the data generated in this study and constitute a substantial body of findings in their own right.

We explicitly integrated and cross-referenced the PBMC dataset from *Sungnak et al.* to provide systemic context and to situate antigen-specific observations within broader immune landscapes during dengue infection. This integration is stated upfront and is intended to build upon a well-characterized framework of systemic immune responses. Importantly, however, the central conclusions of the present manuscript are driven by antigen-specific measurements and analyses, most notably tetramer-defined specificity and quantitative binding-associated readouts, that cannot be derived from PBMC-level profiling alone.

We would also like to clarify the timing of the two studies, as the publication timeline may give the impression of overlap or dependency. The work reported in *Sungnak et al.* was being wrapped up when the present study was initiated, and that manuscript was first submitted in early 2024 before undergoing a prolonged review process across multiple journals, with final acceptance in December 2025. The present study was initiated independently as a complementary line of investigation, with a distinct experimental workflow and a different primary biological objective. Accordingly, the apparent temporal overlap between the two manuscripts reflects the extended review timeline of the earlier study rather than an intentional subdivision of a single dataset or analytical effort.

Overall, while *Sungnak et al.*, which is now published, provides important systems-level context, the present manuscript delivers a distinct informative analysis of antigen-specific CD8+ T cell responses in dengue infection. We therefore believe that the biological findings presented here are sufficiently comprehensive and meaningful to warrant this study standing independently as its own contribution to the field.

Minor Comments:

The introduction contains several imprecise statements requiring revision:

5. Mouse model limitations: References to mouse studies regarding T cell roles in dengue severity are problematic, as DENV neither naturally infects mice nor causes disease in immunocompetent animals. These citations do not adequately support the claims made.

We thank the reviewer for raising this point and agree that mouse models have fundamental limitations in the context of dengue, as DENV does not naturally infect mice and immunocompetent animals do not recapitulate human dengue disease. We therefore agree that observations from mouse studies cannot be used as definitive evidence to support claims regarding T cell-mediated mechanisms underlying dengue severity in humans.

In response to this concern, we have revised the manuscript to soften the statement referencing murine studies and to avoid implying direct evidence for human dengue pathogenesis

Original Text (Introduction):
Line 94: On the one hand, DENV-specific CD8+ T cells have been linked to protective immunity in murine models.

Revised Text (Introduction):
Line 93: On the one hand, DENV-specific CD8+ T cells have been shown to play a role in controlling dengue virus infection in murine models.

6. CYD-TDV and ADE claim: The statement that "in dengue-naive children, CYD-TDV has been linked to an increased risk of hospitalization after primary infection due to ADE" is partially incorrect. While increased hospitalization risk is documented, no mechanistic evidence confirms ADE as the cause.

We thank the reviewer for pointing out the overinterpretation in our original wording. Accordingly, we have revised the manuscript to remove the mechanistic attribution to ADE.

Original Text (Introduction):
Line 86: For instance, in dengue-naive children, CYD-TDV has been linked to an increased risk of hospitalization after primary infection due to ADE.

Revised Text (Introduction):
Line 86: For instance, in dengue-naive children, CYD-TDV has been linked to an increased risk of hospitalization after primary infection.

Reviewer #2 (Remarks to the Author):
This manuscript presents an interesting and technically well-executed study investigating the heterogeneity of DENV-specific CD8+ T cell responses across different disease severity. Using plate-based single-cell RNA sequencing of sorted Tet+ CD8 T cells reactive to both current and prior DENV serotypes, the authors identified distinct subsets characterized by differences in avidity, cytotoxic gene expression, and TCR repertoire. Integration of these data with PBMC and plasma cytokine profiles (from previous under review publication) revealed that clonally expanded CX3CR1+ CD8 T cells are enriched in severe disease and correlate with systemic inflammatory signatures. The study provides valuable insight into the complexity of DENV-specific T cell responses and their potential contribution to either protective or pathogenic immune outcomes. This is a well-written and thoughtfully designed manuscript with a clear biological question and solid technical execution. The conceptual framework linking antigen specificity, TCR avidity, and disease severity is strong and of potential relevance for vaccine development and immune monitoring. The figures are clearly and nicely presented, and the overall flow of the manuscript is good.

We thank the reviewer for the constructive assessment of our work and for recognizing the study as a technically well-executed investigation with insight into the complexity of DENV-specific CD8+ T-cell responses and their potential roles in protective versus pathogenic outcomes.

1. While the study is sophisticated, it remains largely descriptive and lacks the statistical robustness necessary to support its conclusions as a standalone paper. The major limitation is the small sample size, which prevents meaningful comparative analyses between disease groups.

We appreciate the reviewer's concern regarding sample size and statistical robustness. We acknowledge that the cohort size is limited. However, this study was designed to enable high-resolution characterization of antigen-specific CD8+ T cells at the single-cell and clonotype levels rather than broad comparisons across total circulating CD8+ T-cell populations. Such analyses are inherently constrained by the low frequency of epitope-defined dengue-specific CD8+ T cells in PBMC and the requirement for HLA-matched donors. Importantly, sample acquisition represents a major challenge: within five years of household surveillance in the DENFREE cohort, only eight viremic AD cases were identified, and only four of these carried HLA-A*11 or HLA-A*24, which were required for tetramer-based analyses. These factors substantially limit cohort size despite extensive recruitment efforts.

Despite these constraints, many key observations are supported by statistically significant differences with consistent effect directions across analyses (Fig. 3). Within this framework, we identified (i) distinct dengue-specific CD8+ T-cell phenotypes across disease severities, (ii) reproducible TCR features compared with matched total CD8+ T-cell populations, and (iii) differences in TCR gene usage and clonotype structure across disease severities. These findings were supported across multiple analytical layers, including integration with independent 10X datasets and flow cytometry validation, indicating that the study provides potential mechanistic insight rather than just being descriptive. In addition, we performed statistical

analyses with careful consideration of study design, including donor-level aggregation, use of non-parametric tests where appropriate, correction for multiple comparisons, and additional analyses suggested by the reviewer (addressed in the next point).

While larger cohorts will be important for future studies, we believe that this work represents a technically and conceptually substantial contribution, as few studies have achieved this level of antigen-specific, single-cell, and TCR-resolved analysis of dengue-specific CD8+ T cells across clinical severity.

2. Most figures, including the flow cytometry (Fig. 1), single-cell clustering (Figure 2), TCR features (Fig. 4), and clonality analyses (Fig. 5), rely on descriptive visualization with insufficient statistical testing. As a result, although the trends are interesting, it is difficult to determine whether they reflect true biological differences or sampling variability. This limitation substantially weakens the impact and interpretability of the findings to a larger cohort of DENV+ patients.

We appreciate the reviewer's concern that several figures rely primarily on descriptive visualization. In the original analysis, quantitative comparisons were already conducted using donor-level statistical tests where appropriate. In response to the reviewer's suggestion, we have now clarified these analyses and performed additional statistical analyses to further examine the observed trends. The results have been incorporated into the revised manuscript and Supplementary Figures.

Figure 1 (flow cytometry)

Figure 1 is intended to provide an overview of the heterogeneity of dengue-specific CD8+ T-cell reactivity across disease severities and time points, rather than to test predefined group differences. Nonetheless, we performed statistical analyses and did not observe significant differences in dengue-specific CD8+ T-cell frequencies or epitope reactivity across disease severity or over the time course (Rebuttal Figure. 7).

Given the limited cohort size and donor heterogeneity, we therefore deliberately avoided overinterpreting these distributions. The absence of differences at the level of epitope reactivity warranted us to investigate whether heterogeneity instead emerged at the transcriptional level at single-cell resolution. Indeed, our subsequent analyses revealed clear differences in dengue-specific CD8+ T-cell types/states across disease severities (Fig. 3).

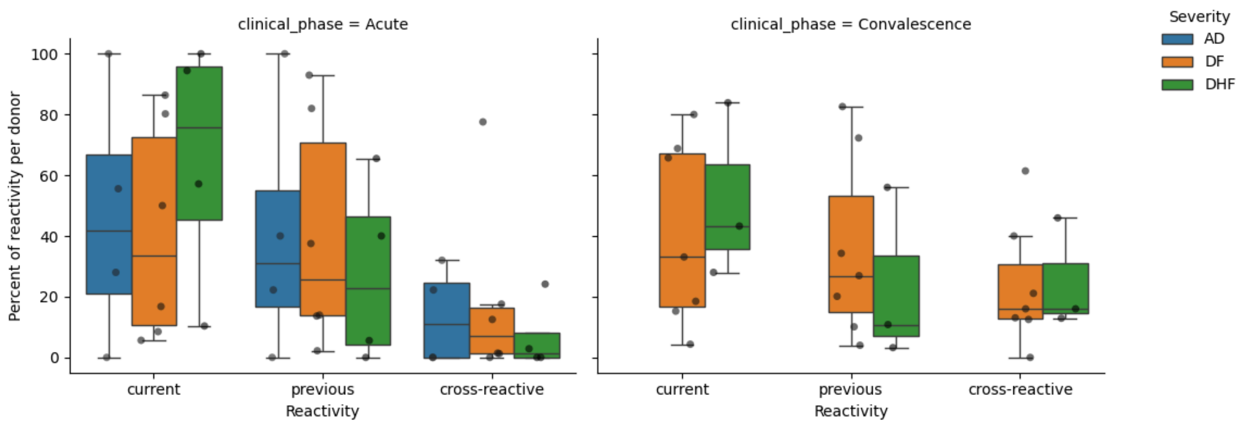

Rebuttal Figure 7. Flow cytometry analysis of dengue-specific CD8+ T-cell epitope reactivity (current, previous, cross-reactive) across severity groups during acute and convalescent phases. No significant differences in overall epitope frequencies were observed across severity or time points.

Figures 2-3 (single-cell clustering and differential cell type abundance analysis)

The clustering in Figure 2 follows a standard unsupervised scRNA-seq workflow aimed at identifying transcriptionally defined CD8+ T-cell subsets/states rather than testing differential abundance[33,34].

Because individual cells are not statistically independent within donors, downstream quantitative comparisons were performed at the donor level after cell-type assignment.

Specifically, after defining cell types, we assess cell-type abundance across disease severity at the donor level (Fig. 3a) and perform non-parametric statistical testing using individual donors as the unit of analysis (Fig. 3b), thereby avoiding inflated significance from a large number of cell counts.

While Figures 4 and 5 initially emphasized descriptive visualization of TCR features and clonality patterns, we agree that formal statistical testing is important to determine whether the observed trends reflect true biological differences rather than sampling variability. We therefore conducted the following additional analyses.

Figure 4 (tetramer fluorescence intensity)

Figure 4 links quantitative tetramer fluorescence intensity to defined CD8+ T-cell phenotypes. To avoid bias toward a single fluorochrome, we first integrated PE-A and APC-A tetramer signals into a composite tetramer fluorescence index (CTFI), calculated as the Euclidean norm:

$$CTFI = \sqrt{(PE - A)2 + (APC - A)2}$$

This metric captures overall tetramer staining intensity across channels and provides a more robust measure of binding strength.

After filtering donor–cluster combinations with fewer than four cells, differences in tetramer fluorescence intensity were evaluated using donor-aware linear mixed-effects regression models[35] , with cluster identity as a fixed effect and donor as a random effect.

The model was specified as:

$$CTFI_{logij} = \beta_0 + \beta_k(\text{Cell cluster}_k) + u_j + \varepsilon_{ij}$$

where

- i indexes individual cells
- j indexes donors.
- Leiden cluster was included as a fixed effect
- Donor ID was included as a random intercept.
- The random effect $u_j \sim N(0, \sigma^2_{donor})$ accounts for inter-donor variation
- $\varepsilon_{ij} \sim N(0, \sigma^2)$ represents residual error.

Mixed-effects modeling demonstrated that CX3CR1 CD8 exhibited significantly higher composite tetramer fluorescence compared to Prolif CD8, Naive/CM CD8, and Int CD8, (Prolif CD8 vs CX3CR1 CD8: $p < 0.001$; Naive/CM vs CX3CR1 CD8: $p = 0.002$; Int CD8 vs CX3CR1 CD8: $p = 0.023$). In contrast, CXCR6 CD8 did not differ significantly from CX3CR1 CD8 ($p = 0.506$) (Rebuttal Figure. 8). Importantly, these differences remained significant after accounting for donor-level variability, indicating that the observed phenotype-specific differences in tetramer staining intensity are not driven by unequal sampling across donors.

Together, these statistically significant differences indicate that CD8+ T-cell clusters defined by transcriptional profiles also exhibit systematic differences in tetramer staining intensity, supporting the existence of distinct TCR-associated phenotypes. This result has now been incorporated into the Results section and Supplementary Figure 6 in the revised manuscript.

Original Text (Result):
Line 296: …we found that during acute infection, high-avidity CD8+ T cells were enriched in CXCR6+ and, to a lesser extent, Prolif CD8 and CX3CR1+ CD8 subsets, while Naive/CM and Int CD8 subsets exhibited lower avidity (Fig. 4a).

Revised Text (Result):
Line 293: …we found that during acute infection, high-avidity CD8+ T cells were significantly enriched in CXCR6+ and CX3CR1+ CD8 subsets, compared to other subsets (Fig. 4a, Supplementary Figure 6)

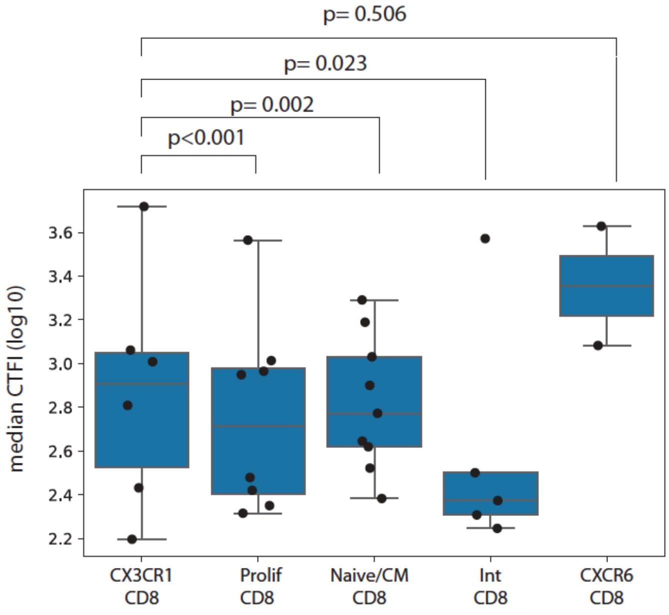

Rebuttal Figure 8. Donor-level median composite tetramer fluorescence index across CD8+ T-cell transcriptional clusters. Each dot represents one donor; box plots summarize cluster-level distributions. Mixed-effects modeling accounting for donor variability confirmed significant differences in tetramer staining intensity across clusters.

Rebuttal Table 4 Linear mixed-effects regression model for log-transformed composite tetramer fluorescence index

Cell cluster (reference = CX3CR1+ CD8)	Estimate (β)	Std. Error	z value	p-value	95% Confidence Interval
**Intercept (CX3CR1+ CD8)**	2.856	0.098	29.065	<0.001	2.663 to 3.049
Proliferating CD8	-0.125	0.033	-3.813	<0.001	-0.190 to -0.061
Naive/CM CD8	-0.117	0.038	-3.043	0.002	-0.192 to -0.042
Intermediate CD8	-0.106	0.047	-2.272	0.023	-0.198 to -0.015
CXCR6+ CD8	-0.027	0.040	-0.664	0.506	-0.105 to 0.052

Dependent variable: CTFI_log
Model: CTFI_log ~ Cell cluster + (1 | Donor)
Number of observations: 776 cells
Number of donors: 14
Estimation method: Maximum likelihood

Figure 5 (TCR sequences and repertoire analysis)

Figure 5 investigates whether the TCR repertoire of GTS-specific CD8+ T cells differs between clinical severity groups. To address the reviewer on statistical analyses, we additionally performed hierarchical clustering on paired VJ gene usage (TRA_V-TRA_J and TRB_V-TRB_J) derived from single-cell TCR data. TCR_VJ clusters were defined based on separation of major branches in the hierarchical dendrogram, reflecting discrete groupings of related paired VJ gene usage patterns.

In the acute phase, several VJ clusters were severity-specific, whereas one VJ cluster showed partial overlap across severity groups, suggesting shared repertoire usage. To statistically validate these observations, we quantified the number of cells within each zone across severity outcomes and performed Fisher-Freeman-Halton's exact test using Monte Carlo simulation (20,000 replicates). Fisher-Freeman-Halton's exact test was used to assess the overall association between VJ cluster distribution and severity. The analysis demonstrated a significant association between VJ cluster distribution and severity in the acute phase ($p = 4.99 \times 10^{-5}$), indicating differential VJ gene usage across disease outcomes (Rebuttal Figure 9).

To determine which TCR cluster contributed to this association, we examined standardized residuals from the chi-square test (Rebuttal Figure 10). Standardized residuals were used to identify enrichment or depletion patterns across individual VJ cluster-severity combinations, whereas chi-square test was used to evaluate the overall association between VJ cluster distribution and disease severity. This analysis showed that VJ cluster 2 was enriched in DF, VJ cluster 3 was enriched in AD, and VJ cluster 4 was enriched in DHF, while VJ cluster 1 showed weaker differentiation, particularly in TRB_VJ, suggesting partial sharing of TRB repertoire features between DF and DHF. Effect-size analysis using Cramer's V demonstrated strong non-random associations between VJ zone and severity (TRA_VJ = 0.95; TRB_VJ = 0.72).

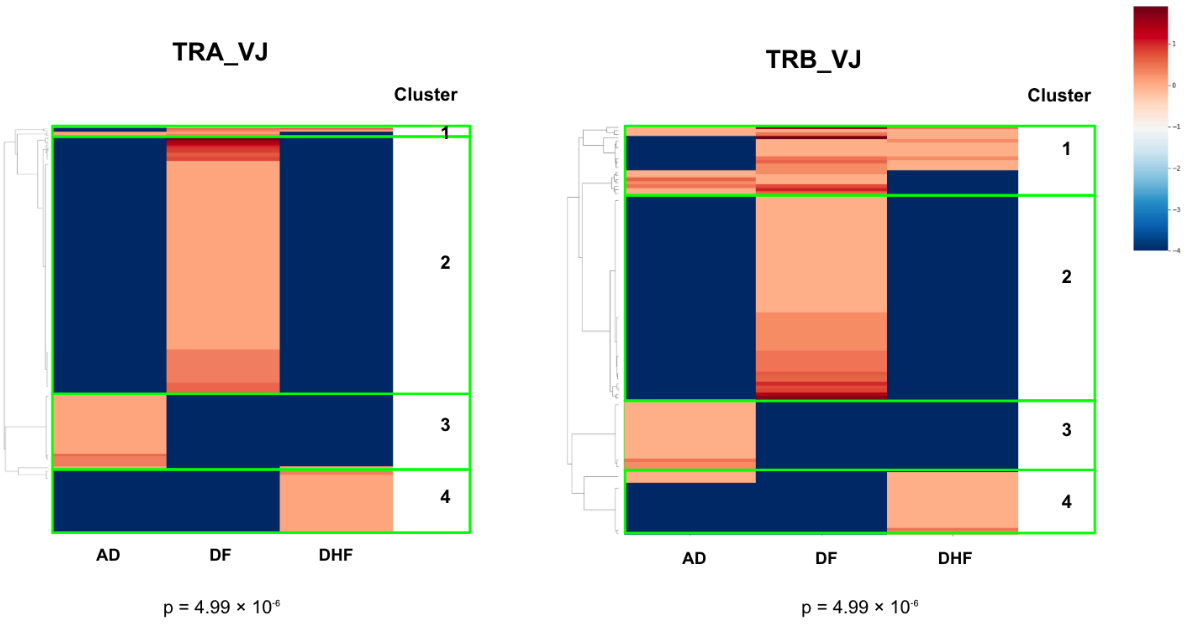

Rebuttal Figure 9. Hierarchical clustering heatmaps of paired VJ gene usage (TRA_VJ and TRB_VJ) in GTS-specific CD8+ T cells across disease severity groups (AD, DF, DHF) during the acute phase. Distinct TCR clusters (1-4), defined by separation of major dendrogram branches, exhibit significantly different VJ usage patterns associated with clinical severity ($p = 4.99 \times 10^{-5}$).

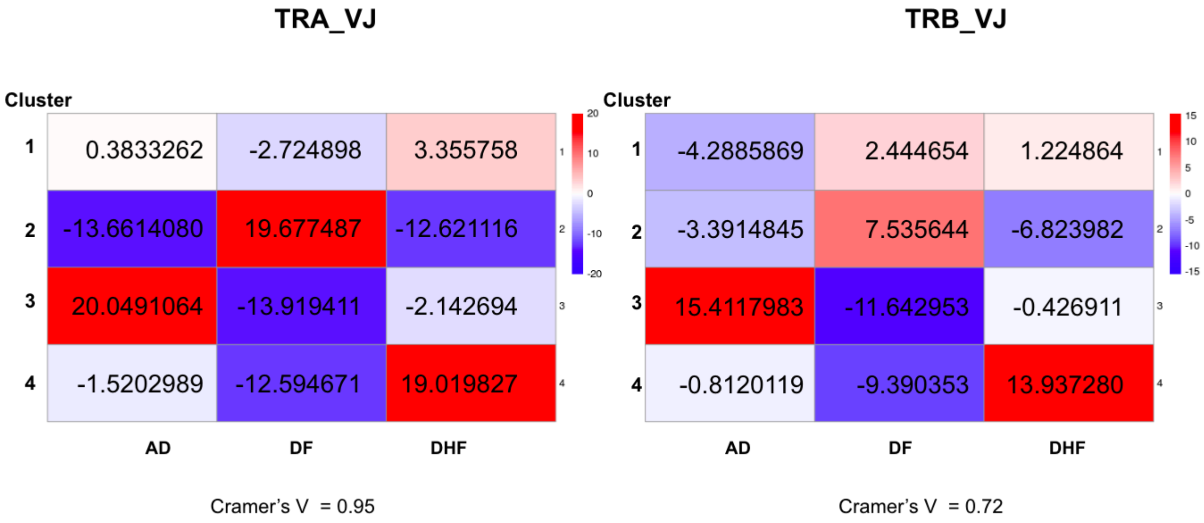

Rebuttal Figure 10. Standardized residual heatmaps showing enrichment (red) or depletion (blue) of TCR clusters across severity groups (AD, DF, DHF) for TRA_VJ and TRB_VJ usage. Cluster 2 was enriched in DF, Cluster 3 in AD, and Cluster 4 in DHF, while Cluster 1 showed

weaker differentiation. Strong associations were observed between TCR cluster distribution and severity (Cramér's V: TRA_VJ = 0.95; TRB_VJ = 0.72).

In the convalescent phase, a similar overall structure was observed, with both shared and severity-specific VJ clusters. Fisher-Freeman-Halton's exact test again showed significant differences in zone distribution across severity groups ($p < 0.05$), indicating repertoire bias in the convalescent phase. Standardized residual analysis showed that, for TRA_VJ, VJ cluster 1 was enriched in DF and VJ cluster 2 in DHF, whereas VJ cluster 3 displayed weaker residual values consistent with shared usage. For TRB_VJ, VJ cluster 1 was enriched in DHF, VJ cluster 2 in DF, and VJ cluster 3 showed weak residuals, suggesting overlap between severity groups. Cramer's V values (TRA_VJ = 0.81; TRB_VJ = 0.57) confirmed continued non-random associations between VJ usage and severity during convalescence

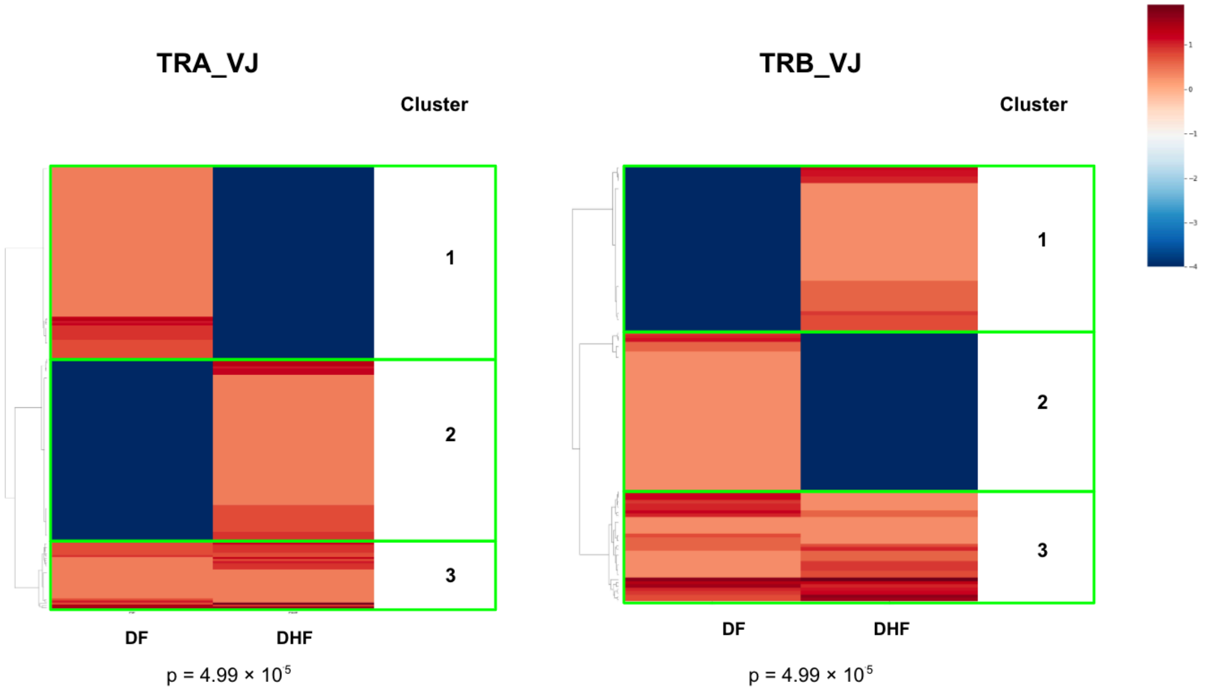

Rebuttal Figure 11. Hierarchical clustering heatmaps of paired VJ gene usage (TRA_VJ and TRB_VJ) in GTS-specific CD8+ T cells across disease severity groups (DF, DHF) during the acute phase. Distinct TCR clusters (1–3), defined by separation of major dendrogram branches, exhibit significantly different VJ usage patterns associated with clinical severity ($p = 4.99 \times 10^{-5}$).

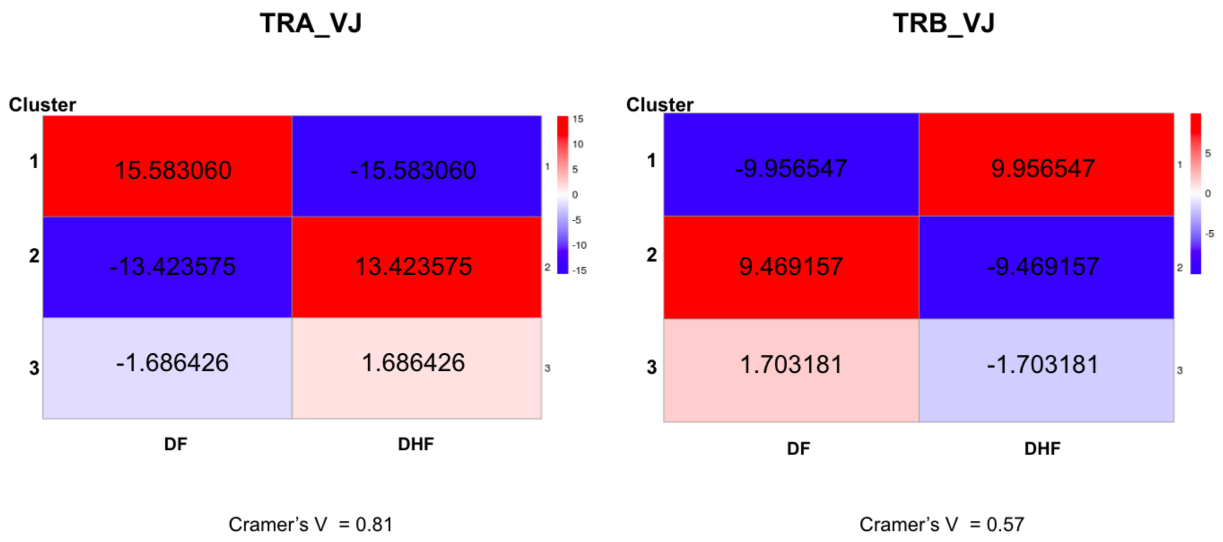

Rebuttal Figure 12. Standardized residual heatmaps showing enrichment (red) or depletion (blue) of TCR clusters across severity groups (DF, DHF) for TRA_VJ and TRB_VJ usage. For TRA_VJ, Cluster 1 was enriched in DF, Cluster 2 in DHF, while Cluster 3 showed weaker differentiation. For TRB_VJ, Cluster 1 was enriched in DHF, Cluster 2 in DF, while Cluster 3 showed weaker differentiation. Strong associations were observed between TCR cluster distribution and severity (Cramér's V: TRA_VJ = 0.81; TRB_VJ = 0.57).

This result has now been incorporated into the Results section and Supplementary Figure 8 in the revised manuscript

Added Text (Result):
Line 332: Notably, VJ cluster distribution was significantly associated with disease severity (Supplementary Figure 8)

To further evaluate global TCR repertoire relationships across disease severity with respect to individual donors at donor level, we performed principal component analysis (PCA) based on VDJ gene usage from both TCRα and TCRβ chains, including samples from acute and convalescent phases. The PCA revealed that AD samples clustered separately from symptomatic dengue (SD) groups (DF and DHF), indicating distinct repertoire composition. In contrast, DF and DHF samples are largely localized near each other, suggesting substantial overlap in VDJ gene usage patterns. Although one DHF outlier was observed, most symptomatic samples showed similar repertoire positioning in PCA space (Rebuttal Figure. 13). Consistent with the PCA distribution, hierarchical clustering based on PC1 and PC2 coordinates identified three major clusters of donors (Rebuttal Figure 14). The distribution of disease severity across these clusters was significantly associated with clinical outcome (Fisher-Freeman-Halton exact test, $p = 0.0083$), supporting distinct TCR repertoire organization between AD and symptomatic dengue donors.

Taken together, these results support the conclusion that the TCR repertoire in asymptomatic infection is distinct, whereas symptomatic dengue cases (DF and DHF) share more similar global VDJ usage profiles across both TCRα and TCRβ chains.

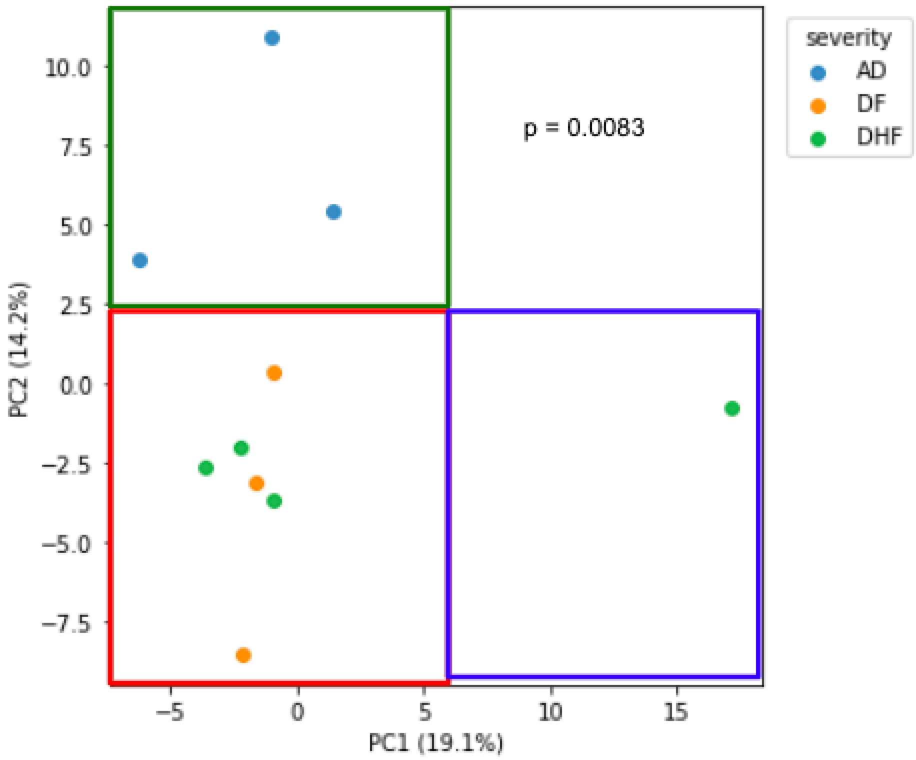

Rebuttal Figure 13. Principal component analysis (PCA) of donor-level TCR VDJ gene usage (TRA and TRB combined) from the integrated dataset combining 10x and SS2 across disease severity groups (AD, DF, DHF). AD samples cluster separately from symptomatic dengue (DF and DHF), which largely overlap in PCA space. Three clusters were defined based on the PCA distribution, and the association between cluster membership and disease severity was evaluated using the Fisher–Freeman–Halton exact test, showing a significant relationship (p = 0.0083).

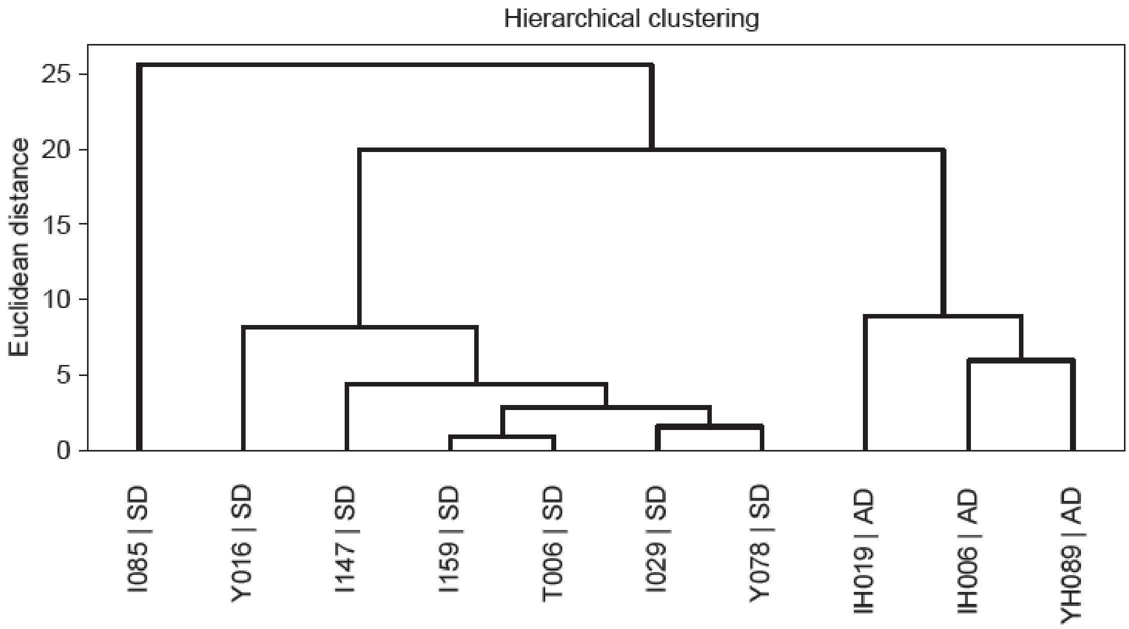

Rebuttal Figure 14. Hierarchical clustering dendrogram of donor-level samples based on Euclidean distances calculated from PC1 and PC2 coordinates derived from PCA of global TCRα and TCRβ V(D)J gene usage. Each leaf represents an individual donor labeled by donor ID and disease group. Clustering reveals separation of AD donors from symptomatic dengue (SD; DF and DHF) samples, consistent with the PCA distribution.

The results have now been incorporated into the Results section and Supplementary Figure 10 in the revised manuscript.

Added Text (Results):
Line 339: …, consistent with principal component analysis (PCA) of global TCR repertoire composition, where resulting clusters showed significant association with AD and SD labels (Supplementary Fig.10)

Finally, as suggested by the reviewer, we have revised the Methods section to describe additional statistical tests used in the analyses.

Added Text (Method):
Line 741: The composite tetramer fluorescence index across CD8+ T-cell clusters was evaluated using a linear mixed-effects regression model with the cell cluster as a fixed effect and donor as a random intercept, implemented in the statsmodels package (Supplementary Methods). Associations between TCR VJ cluster distribution and disease severity were assessed using the Fisher-Freeman-Halton exact test with Monte Carlo simulation (scipy.stats), with effect sizes estimated using Cramer's V (Supplementary Methods). Global differences in TCR repertoire composition were evaluated using PCA of

donor-level V(D)J gene usage, and the association between cluster membership and disease severity was evaluated using the Fisher–Freeman–Halton exact test.

3. As presented, the dataset appears too narrow to represent the full spectrum of DENV-specific immune responses and the conclusions therefore remain speculative.

Dengue immune responses are highly diverse, and no single dataset so far has captured the full spectrum of DENV-specific CD8+ T-cell states. However, the focus on selected epitopes in this study was intentional by design. Specifically, the goal was not to exhaustively characterize all possible epitope-HLA combinations, but rather to provide a high-resolution analysis of naturally circulating DENV-specific CD8+ T cells across disease severities, including asymptomatic ones, and infection phases using immunodominant epitope-HLA combinations that are highly relevant in endemic populations such as Thailand. We believe this targeted strategy provides biologically meaningful insight despite not representing the complete epitope landscape.

Notably, as detailed in our response to Reviewer 1, the selected epitopes, particularly the HLA-A*11-restricted GTS epitopes, represent highly immunodominant targets rather than narrow or minor components of the DENV-specific CD8+ T-cell response. In our cohort, GTS-specific CD8+ T cells were detected in 10 of 10 HLA-A11+ donors across the infection course and accounted for approximately 0.2–30% of total circulating CD8+ T cells during acute infection (Rebuttal Figure. 2). These frequencies are comparable to or exceed those reported for well-established immunodominant epitopes in other viral infections[10-14]. Therefore, the patterns described in this study reflect substantial and biologically meaningful components of the antiviral CD8+ T-cell response rather than rare or narrowly restricted populations.

In addition, the conclusions are not derived from the Smart-seq2 tetramer-sorted dataset alone. Instead, this study integrates multiple orthogonal datasets and experimental platforms, including tetramer-based FACS sorting of CD8+ T cells recognizing paired current and previous DENV serotypes, plate-based (Smart-seq2) single-cell transcriptomics, droplet-based (10x Genomics) single-cell transcriptomics and TCR sequencing on PBMCs based on 24 individuals spanning AD, DF, and DHF, and matched plasma cytokine measurements. This integrated multi-platform approach substantially broadens the biological context beyond a single modality.

Importantly, key observations such as the enrichment of CX3CR1+ CD8+ T cells in DHF and intermediate CD8+ T-cell states in AD were independently reproduced in clonally expanded CD8+ T-cell populations identified in PBMC-level datasets, which likely include antigen-responsive CD8+ T cells beyond tetramer-defined CD8+ T cell. These cellular states also showed consistent associations with cytokine profiles, providing orthogonal support for the observed correlations.

We therefore believe that the key conclusions are supported by the data and extend beyond being merely speculative. While cross-sectional analyses cannot by themselves establish definitive causal mechanisms, the reported findings represent statistically supported associations observed across multiple datasets and analytical approaches. To further clarify

interpretation, we have revised sections of the Discussion to more explicitly distinguish direct observations from proposed biological implications.

Original Text (Discussion):
Line 492: However, the mechanisms driving this phenotype and role in dengue pathogenesis remain to be experimentally validated.

Revised Text (Discussion):
Line 490: However, these observations are based on cross-sectional associations, and further mechanistic studies will be required to determine whether these cellular states play a causal role in dengue disease progression.

Finally, although broader HLA and epitope coverage will be important for future studies, to our knowledge this work represents the first antigen-specific, single-cell transcriptional and TCR-resolved analysis of human DENV-specific CD8+ T cells across clinical severity including AD. Thus, despite inherent limitations in epitope scope, the conclusions are supported by multiple complementary lines of evidence rather than isolated descriptive observations.

4. Another concern is novelty relative to previous work. Similar datasets from the same or related groups have described phenotypic and transcriptional heterogeneity of DENV-specific T cells, including signatures of activation, exhaustion, and clonal expansion. This manuscript extends those findings on the Tet+ CD8 T cells but does not introduce a sufficiently distinct mechanistic insight to justify a separate publication.

We thank the reviewer for raising this point and appreciate the opportunity to clarify the relationship between the present study and Sungnak et al., now published in *Science Translational Medicine* (DOI: 10.1126/scitranslmed.ads5932)[32]. While both studies arise from the same broader research program, the present manuscript was designed, executed, and analyzed as a distinct follow-up project and is conceptually, experimentally, and analytically independent, with its own primary biological questions and experimental strategies.

Sungnak et al. focuses on global PBMC-level immune dynamics during dengue infection, characterizing broad changes in immune cell composition and total CD8+ T-cell clonal expansion across disease severities. In contrast, the current study directly interrogates epitope-defined, pHLA tetramer-identified dengue-specific CD8+ T cells, enabling precise examination of bona fide antigen-reactive populations while excluding bystander-activated cells. This approach allows discrimination between CD8+ T cells reactive to the current versus serologically inferred previous infecting serotypes and reveals phenotypic heterogeneity beyond conventional CD8+ T cell classifications.

Importantly, the primary analyses and conclusions of the present study rely on experimental readouts unique to this work, most notably quantitative tetramer fluorescence intensity as a proxy for relative pHLA-TCR binding strength. By integrating antigen specificity, TCR gene usage, clonotype architecture, avidity-related metrics, and transcriptional phenotypes across

severity groups, we directly link TCR-pHLA interaction features to functional CD8+ T-cell states and clinical outcomes. These biological insights, particularly regarding heterogeneity within antigen-specific CD8+ T cells and their association with disease severity, are supported directly by data generated in this study and represent findings not demonstrated in the prior PBMC-level analysis.Although we integrated and cross-referenced the PBMC dataset from Sungnak et al. to provide systemic context, the central conclusions of this manuscript are driven by antigen-specific and binding-associated measurements that cannot be derived from PBMC profiling alone.

Finally, we would also like to clarify a point related to publication timing that may give the impression of overlap between the two studies. Sungnak et al. was submitted in early 2024 and underwent a prolonged multi-journal review process before final acceptance in December 2025. The present study was initiated independently as a complementary but distinct investigation, with a separate experimental workflow and analytical framework. The apparent temporal overlap reflects publication timelines rather than subdivision of a single integrated study.

Overall, while *Sungnak et al.*, which is now published, provides important systems-level context, the present manuscript delivers a distinct informative analysis of antigen-specific CD8+ T cell responses in dengue infection. We therefore believe that the biological findings presented here are sufficiently comprehensive and meaningful to warrant this study standing independently as its own contribution to the field.

5. The work would be more impactful if positioned as a focused report or as part of a broader cohort analysis integrating additional immunological parameters or larger cohorts. Expanding the sample size, improving the statistical analyses, and including confirmatory experiments would significantly enhance the robustness and translational relevance of the study.

We thank the reviewer for the suggestion regarding study positioning, cohort size, and robustness. We acknowledge that expanding cohort size, epitope coverage, and additional functional validation would further strengthen translational scope of the work. However, the current sample size reflects practical and biological constraints associated with capturing acute-phase, Tet+ DENV-specific CD8+ T cells, particularly from AD cases, which are extremely rare. Identifying such cases require long-term longitudinal household surveillance, and only a limited number of HLA-matched individuals were eligible for tetramer-based analyses. Given these constraints, careful experimental design and execution were prioritized, including standardized sample processing and donor selection from a well-characterized cohort with matched clinical parameters (including age, sex, and viremia) to minimize confounding effects.

Despite these constraints, the conclusions do not rely on the tetramer-sorted dataset alone. Key findings derived from the profiled antigen-specific CD8+ T-cell populations were extended and evaluated in larger PBMC-level datasets, where statistically significant associations were observed across broader cohorts. In addition, confirmatory experiments, including

flow-cytometric validation of CX3CR1-associated CD8+ T-cell states, provided independent experimental support for major conclusions.

In the revised manuscript, we further strengthened statistical rigor by applying donor-aware analyses, mixed-effects modeling, and conservative multiple-testing corrections, and by ensuring that conclusions remain restricted to statistically supported observations. Moreover, multiple orthogonal immunological parameters, including serological infection history (PRNT, HI, ELISA), plasma cytokine measurements, flow-cytometric validation, and integration with matched 10x datasets, are explicitly incorporated into the analysis framework (main text and Supplementary Information).

We have clarified this positioning in the revised Discussion, framing the present work as a focused antigen-specific analysis that complements larger cohort studies, with future work needed to extend these findings to broader cohorts and epitope coverage.

Original Text (Discussion):
Line 542: The relatively small cohort size and the limited range of HLAs and epitopes analyzed may restrict the generalizability of our results. Further studies with broader pHLA and larger sample size are warranted.

Revised Text (Discussion):
Line 542: The relatively small cohort size and number of Tet+ cells in certain donors, together with the restricted range of HLAs and epitopes analyzed may restrict the generalizability of our results. Further studies with broader pHLA and larger sample size, and more efficient antigen-specific T-cell capture techniques are warranted.

Reviewer #3 (Remarks to the Author):

The paper assesses an important question in the field, shedding light on the roles of DENV-specific CD8+ T cells in dengue disease pathogenesis. The cohort includes asymptomatic infected individuals, which provide unique insights into a protective immune response. In addition, the study focuses on the T cell response during acute (secondary) infection, which is unlike most studies focusing on the convalescent response.

We thank the reviewer for the summary of our work and for noting the inclusion of asymptomatic individuals that provide unique insights into a protective immune response and the focus on acute secondary infection as distinctive features of the study.

1. Even though the research question is relevant, and the cohort and methods used are unique and cutting edge, there remain important limitations with regard to sample size (both in terms of patients included and cells investigated), which limit the validity of the interpretation of the data.

We acknowledge that the cohort size, both in the number of donors and Tet+ cells, is limited. This reflects two biological constraints. First, we deliberately focused on well-characterized immunodominant NS3 epitopes (HLA-A*11-restricted GTS and HLA-A*24-restricted NYA), which account for a substantial fraction of the total DENV-specific CD8+ T-cell response. While this enables more precise antigen-specific analysis within clinically relevant HLA backgrounds, it inherently limits the pool of eligible HLA-matched participants. Second, asymptomatic dengue cases with detectable viremia are rare and can only be identified through intensive prospective household surveillance (8 of 179 cases over 5 years)[28], further restricting sample availability. To reduce potential confounding, donors were carefully matched for sex, age, and viremia level.

Importantly, despite the limited sample size, severity-associated differences, including dengue-specific CD8+ T-cell phenotypes and TCR repertoire features, were evaluated using statistical tests that account for donor-level variation. Key comparisons reached statistical significance, indicating effect sizes sufficiently large to be detectable even within this cohort and supporting the robustness of the observations. Additionally, comparisons that did not reach statistical significance are explicitly indicated.

To further strengthen robustness, we also integrated our data with matched droplet-based PBMC single-cell transcriptomic and TCR datasets, enabling recovery of additional shared clonotypes and inference of putative dengue-specific CD8+ T-cell populations based on TCR similarity. Key findings were also further validated by independent flow cytometry analyses.

To improve clarity, we have revised the manuscript to more clearly describe the statistical testing framework and the interpretation of results given the cohort size.

Original Text (Discussion):

Line 542: The relatively small cohort size and the limited range of HLAs and epitopes analyzed may restrict the generalizability of our results. Further studies with broader pHLA and larger sample size are warranted.

Revised Text (Discussion):
Line 542: The relatively small cohort size and number of Tet+ cells in certain donors, together with the restricted range of HLAs and epitopes analyzed may restrict the generalizability of our results. Further studies with broader pHLA and larger sample size, and more efficient antigen-specific T-cell capture techniques are warranted.

2. Dengue disease is very dynamic, and changes in immune cell composition and activation occur rapidly. Patients are at risk to develop severe disease in a small window after onset of symptoms, usually 3-5 days post onset of symptoms. Here, it is unclear how many days after onset of symptoms the DF and DHF patients were included (this is a better measurement rather than days before defervescence).

We thank the reviewer for raising this point regarding timing of sample collection. In this study, we intentionally used defervescence (D0) as the primary temporal reference point because it represents a well-defined and clinically relevant milestone in dengue progression. In contrast, reported symptom onset may be more subjective and variable across patients, particularly in distinguishing early febrile symptoms from non-specific prodromal signs.

All symptomatic DF and DHF samples in the febrile phase used here were collected 24-48 hours before defervescence. These samples correspond approximately to day 3–5 after symptom onset (Rebuttal Table 5). The exact days post symptom onset (DPO) for each DF and DHF sample are now incorporated in Supplementary Table 1 and fall within a comparable sampling window across patients and between severity groups.

Rebuttal Table 5. Participant demographics, clinical parameters and applied single-cell RNA-seq techniques (corresponding to Supplementary Table 1 in revised manuscript)

Sample ID	ID	HLA	Severity	Days post-onset of symptoms (DPO)	Serotype	Age	Sex	Viral copies/ml (qRT-PCR)	Primary/ Secondary infection HI
IH006	1	A*11	AD	N/A	DENV4	28	F	1.03E+07	Secondary
IH019	2	A*11	AD	N/A	DENV3	63	M	1.64E+06	Secondary
IH023	3	A*24	AD	N/A	DENV3	25	M	1.17E+04	Secondary
YH045	4	A*02	AD	N/A	DENV4	19	M	2.19E+06	Unknown
YH049	5	A*11	AD	N/A	DENV4	34	F	5.37E+06	Secondary
YH089	6	A*11	AD	N/A	DENV1	38	F	1.06E+03	Primary
H001	7	A*02	AD	N/A	DENV3,4	18	F	3.60E+04, 1.47E+07	Secondary
H030	8	A*02	AD	N/A	DENV3	39	M	5.31E+04	Secondary
I160	9	A*02	DF	3	DENV3	39	M	2.76E+04	Secondary
T010	10	A*24	DF	5	DENV3	22	F	5.34E+03	Secondary
T006	11	A*11	DF	4	DENV4	15	F	1.77E+06	Secondary
Y029	12	A*24	DF	3	DENV4	60	F	7.04E+06	Secondary
Y016	13	A*24 & A*11	DF	N/A	DENV4	46	F	6.99E+08	Secondary
I147	14	A*11	DF	5	DENV3	18	M	1.96E+07	Secondary
I171	15	A*24	DF	3	DENV4	65	F	2.65E+06	Secondary
I109	16	A*24	DF	5	DENV1	38	M	3.52E+02	Secondary
I085	17	A*11	DHF	3	DENV3	17	M	6.87E+03	Secondary
Y078	18	A*24 & A*11	DHF	4	DENV3	23	F	3.35E+04	Secondary
I010	19	A*02	DHF	4	DENV3	26	M	2.03E+07	Secondary
I016	20	A*02	DHF	5	DENV4	34	F	5.79E+06	Secondary
I159	21	A*11	DHF	3	DENV4	38	M	1.41E+07	Secondary
I024	22	A*02	DHF	5	DENV4	33	F	8.61E+05	Secondary
I029	23	A*11	DHF	5	DENV4	20	F	1.66E+06	Secondary
I075	24	A*24	DHF	5	DENV1	15	F	7.54E+06	Secondary

3. A.) In addition, the stage of infection in asymptomatic cases cannot be determined. This is an important limitation and should be mentioned in the paper.

Thank you for pointing this out. We have added text in the Introduction section

Added Text (Introduction):
Line 111: Because symptom onset cannot be defined in these AD individuals, their stage of infection cannot be determined. Nonetheless,...

B.) Maybe all ASD cases are earlier in their infection trajectory and therefore display less CX3CR1+ CD8+ T cells.

We appreciate this alternative interpretation. In our dataset, however, AD donors were matched by viremia level to symptomatic DF/DHF donors sampled during the febrile phase. As viral load varies dynamically during dengue infection, this, arguably, reduces the possibility that one group systematically represents an earlier point in the infection trajectory, although viral load alone does not fully define infection stage. In addition, most AD and SD donors were not recruited from the same household. Only one AD-DF pair originated from a shared household (IH023 in AD and I160 in DF), suggesting that staggered infection timing due to potential household transmission are unlikely to substantially bias the overall comparison.

Furthermore, within symptomatic infection, DF and DHF samples were collected at the same defined clinical time point (Day -1 before defervescence). Under this controlled timing, DHF cases still showed higher frequencies of CX3CR1+ CD8+ T cells. This observation is consistent with a severity-associated difference that is not readily explained by sampling timing alone. Nevertheless, because the infection stage in AD individuals cannot be precisely defined, we acknowledge that the effects of infection timing cannot be fully excluded.

We have revised the manuscript to clarify this limitation and its implications for interpreting the AD/SD comparison.

Added Text (Discussion):
Line 528: Nevertheless, because the precise infection stage in AD individuals cannot be definitively determined, the potential influence of infection timing on these observations cannot be fully excluded.

4. During secondary infection, usage of PRNT levels to evaluate previous infecting serotypes should be done with caution, especially when only the acute phase timepoint is available. This should be clearly stated in the manuscript.

We agree that inference of previous infecting serotypes based on PRNT titres during secondary dengue infection should be interpreted with caution, particularly when only acute-phase samples are available.

To address the reviewer's concern, we have revised the terminology from "previously infecting serotype" to "serologically inferred dominant previously infecting serotype" to more accurately reflect the serology-based inference used in this study. Specifically, the serologically inferred dominant previously infecting serotype was defined as the non-current DENV serotype with the highest PRNT titer during the acute phase. The PCR-confirmed infecting serotype during the acute episode was still designated as the current serotype, even if it exhibited the highest PRNT titer. This classification reflects the dominant serological imprint excluding the ongoing infection and does not imply definitive virological confirmation of historical infection. For simplicity in figures, we now refer to this as the "inferred previous serotype". We clearly state in the revised Abstract, Result, Methods and Discussion that this designation represents serological inference rather than definitive assignment of prior infecting serotypes.

Original Text (Result):
Line 162: …previously infecting DENV serotypes, as determined via qPCR and plaque reduction neutralization test (PRNT) assays, respectively.

Revised Text (Result):
Line 159: …serologically inferred dominant previously infecting DENV serotypes. In this study, the currently infecting serotype was determined by qPCR, while the serologically inferred dominant previously infecting serotype was defined as the non-current serotype with the highest plaque reduction neutralization test (PRNT) titer (SupplementaryTable 2).

Original Text (Result):
Line 343: …, TCRs targeted serotypes from previous infections, regardless of CD8+ T cell subset (Fig. 5a)

Revised Text (Result):
Line 342: …, TCRs preferentially targeted serologically inferred dominant previously infecting serotypes, regardless of CD8+ T cell subset (Fig. 5a).

Added Texted (Abstract):
Line 62: serologically inferred dominant previous DENV serotypes…

Added Text (Result):
Line 180: …serologically inferred dominant previous serotype
Line 282: …serologically inferred dominant previously infecting serotypes

Added Text (Method):
Line 630: We selected serologically inferred dominant previously infecting serotypes as the non-current DENV serotype with the highest PRNT titer in acute phase.

Added Text (Discussion):
Line 460: …serologically inferred dominant previously infecting serotypes

We have also revised the manuscript to acknowledge the limitations of our serology-based infection history inference, clarify the definition of serologically inferred previously infecting serotypes, and include an example illustrating the potential for misclassification. These points have been added to the Discussion.

Original Text (Discussion):
Line 566: Additionally, although primary or secondary infection status was generally determined using antibody-based assays such as PRNT or HI[65], we found one AD case previously classified as primary infection based on antibody assay may actually represent a secondary infection, as indicated by DENV-specific T cell responses to previously encountered serotypes. This observation highlights a potential limitation of antibody-based classification in AD, which is characterized by predominant T cell response and low antibody one.

Revised Text (Discussion):
Line 555: Another limitation relates to the determination of infection history. Primary versus secondary infection status was determined using antibody-based assays such as PRNT or hemagglutination inhibition (HI) assay[65], which may not perfectly reconstruct historical infecting serotypes, particularly in endemic settings. To reflect this limitation, the relevant serotypes assigned to these samples are described as serologically inferred dominant previously infecting serotypes, defined operationally based on neutralization titers rather than definitive virological confirmation historically. Consistent with this limitation, we identified one AD case initially classified as primary based on serology that

may instead represent a secondary infection, as suggested by detectable DENV-specific T-cell responses to inferred previously encountered serotypes. This observation highlights a potential limitation of antibody-based determination of previously infecting serotypes, particularly in AD, which is characterized by substantial T cell responses and relatively lower antibody ones[18]. In addition, serotype-specific neutralizing antibody and CD8+ T-cell responses do not necessarily correlate directly (Supplementary Table 7,8) and may reflect differential or biased immune recall within distinct immune compartments.

5. "biased reactivity to current versus previous serotypes": the data (line 262-276) show a trend, not a statistically significant difference. Pls amend abstract and discussion where needed (eg. Line 61, line 440, etc)

We thank the reviewer for pointing this out. The Abstract and Discussion have been revised to explicitly describe this observation as a trend.

Added Text (Abstract):
Line 65: …, a tendency of biased reactivity to current versus previous serotypes, and specific TCR-related features.

Added Text (Discussion):
Line 460: …, tendency of biased reactivity toward serologically inferred dominant previously infecting serotypes, and elevated expression of genes associated with TCR signaling and cytotoxicity, were expanded in SD cases.

6. How is the serotype specificity of the peptides evaluated? Can the authors be sure that the peptide-specific CD8+ T cells are specific for the current-previous infecting serotype? Data should be included of patients in the convalescent phase after RT-qPCR serotype confirmed primary infection, where serotype-specific reactivity of the used peptides is confirmed.

The NS3 peptides used in our study represent defined serotype-variant epitopes that differ by specific amino acid substitutions corresponding to each DENV serotype. Due to sequence similarity and structural conservation within the HLA-binding groove, some degree of cross-reactivity is expected[3-5,8,20] .

As detailed in our response to **Reviewer 1 point 3**, we performed additional multi-serotype tetramer staining using all four DENV serotype variants with PKI pretreatment in the original cohort. In most donors, the dominant tetramer-binding pattern corresponded to the highest PRNT titer, although cross-reactive populations were also detected (Rebuttal Tables 1 and 2). This pattern supports serotype-preferential recognition with biologically expected cross-reactivity. We also included an attempt to clarify tetramer specificity in the Methods section

Added Text (Method):

Line 726: To assess tetramer specificity across the four DENV serotypes, PBMC samples collected at defervescence (D0) were first stained with LIVE/DEAD Fixable Green Dead Cell Stain (Thermo Fisher Scientific, USA) and treated with dasatinib prior to tetramer staining. Cells were then incubated with serotype-specific HLA tetramers labeled with PE, APC, BV421, and PE-Cy7. Tet+ CD8+ T cells were identified based on tetramer fluorescence above FMO controls. Serotype-specific and cross-reactive CD8+ T cells were determined using Boolean gating in FlowJo™ v10.10 software (shown in Supplementary Table 7-8)

Rebuttal Table 1: PRNT50 titers against DENV1–4 and distribution of serotype-specific and cross-reactive GTS-specific CD8+ T cells across HLA-A*11 donors

Donor_ID	Severity	PRNT50 titer				
		Time-point	DENV1	DENV2	DENV3	DENV4
I147	DF	D1	2108	13286	2960	2555
		F1	6350	372805	26783	61984
T006	DF	D1	42206	30915	17840	16113
		F1	308937	129567	45104	37607
Y016	DF	D1	N/A	N/A	N/A	N/A
		F1	N/A	N/A	N/A	N/A
I085	DHF	D1	25703	64353	3004	14401
		F1	6365	136271	4443	41112
I159	DHF	D1	9	315	48	40
		F1	7127	51718	4797	25201
Y078	DHF	D1	1558	4923	1582	6366
		F1	N/A	N/A	N/A	N/A
I029	DHF	D1	6148	11956	6739	4291
		F1	N/A	N/A	N/A	N/A

High

Low

Current
Inferred previous

Sample_ID	Percent of CD8 T cells					
	Serotype-specific			Cross-reactive		
	Tet-GTS1	Tet-GTS2	Tet-GTS3/4	Tet-GTS1|2	Tet-GTS1|3/4	Tet-GTS2|3/4
I147D0	0.387	0.035	0.228	0.121	0.314	0.125
T006D0	0.65	0.225	1.397	1.158	1.437	0.467
Y016D0	0.71	0.098	0.177	0.095	0.212	0.084
I085D0	0.215	0.224	0.753	0.633	1.272	0.615
I159D0	1.74	0.185	0.188	0.065	0.105	0.062
Y078D0	0.316	0.066	0.199	0.036	0.081	0.01
I029D0	0.169	0.041	0.813	0.503	1.152	0.498

Rebuttal Table 2: PRNT50 titers against DENV1–4 and distribution of serotype-specific and cross-reactive NYA-specific CD8+ T cells across HLA-A*24 donors

Donor_ID	Severity	PRNT50 titer				
		Time-point	DENV1	DENV2	DENV3	DENV4
IH023	AD	D1	404	1713	1399	1254
		F1	N/A	N/A	N/A	N/A
T010	DF	D1	3590	7424	1652	54194
		F1	5944	16706	7618	38598
Y029	DF	D1	748	43559	3928	4632
		F1	41244	20879	28983	38806
I109	DF	D1	4947	3646	33863	23419
		F1	8439	14483	26914	45461
I171	DF	D1	8140	20770	27268	13709
		F1	42881	29865	38709	86973
I075	DHF	D1	3423	41067	4533	340
		F1	19546	17044	142196	26523

High

Low

Current

Inferred previous

Sample_ID	Percent of CD8 T cells									
	Serotype-specific				Cross-reactive					
	Tet-NYA1+	Tet-NYA2+	Tet-NYA3+	Tet-NYA4+	Tet-NYA1|2	Tet-NYA1|3	Tet-NYA1|4	Tet-NYA2|3	Tet-NYA2|4	Tet-NYA3|4
IH023	0.011	0.359	0.378	0.452	0.005	0.005	0.005	0.183	0.21	0.279
T010D0	0	0.234	0.315	3.098	0.019	0.019	0.017	2.895	3.358	3.342
Y029D0	0	0.185	0.539	2.797	0.002	0.002	0.002	0.487	0.613	0.913
I109D0	0	0.211	0.369	3.121	0.018	0.019	0.019	2.448	2.994	2.8
I171D0	0.012	2.08	0.844	1.024	0.009	0.009	0.009	3.696	0.691	0.685
I075D0	0	0.179	0.28	1.224	0.016	0.006	0.003	0.58	0.726	0.721

Regarding the suggestion to include convalescent samples from RT-qPCR-confirmed primary infections, this is not feasible in our endemic setting, where true seronegative primary cases are rare. However, previous studies using similar serotype-variant peptides and pHLA tetramers have demonstrated epitope specificity by analyzing CD8+ T-cell clones isolated from RT-qPCR-confirmed primary infections during the convalescent phase. These studies demonstrated stronger tetramer binding to the infecting serotype while also detecting measurable cross-reactivity to heterologous variants[4] (Rebuttal Figure 15).

Taken together, our multi-tetramer analysis and prior validation studies support that the peptides used in this manuscript capture biologically meaningful serotype-preferential CD8+ T-cell responses alongside cross-reactivity characteristic of dengue infection

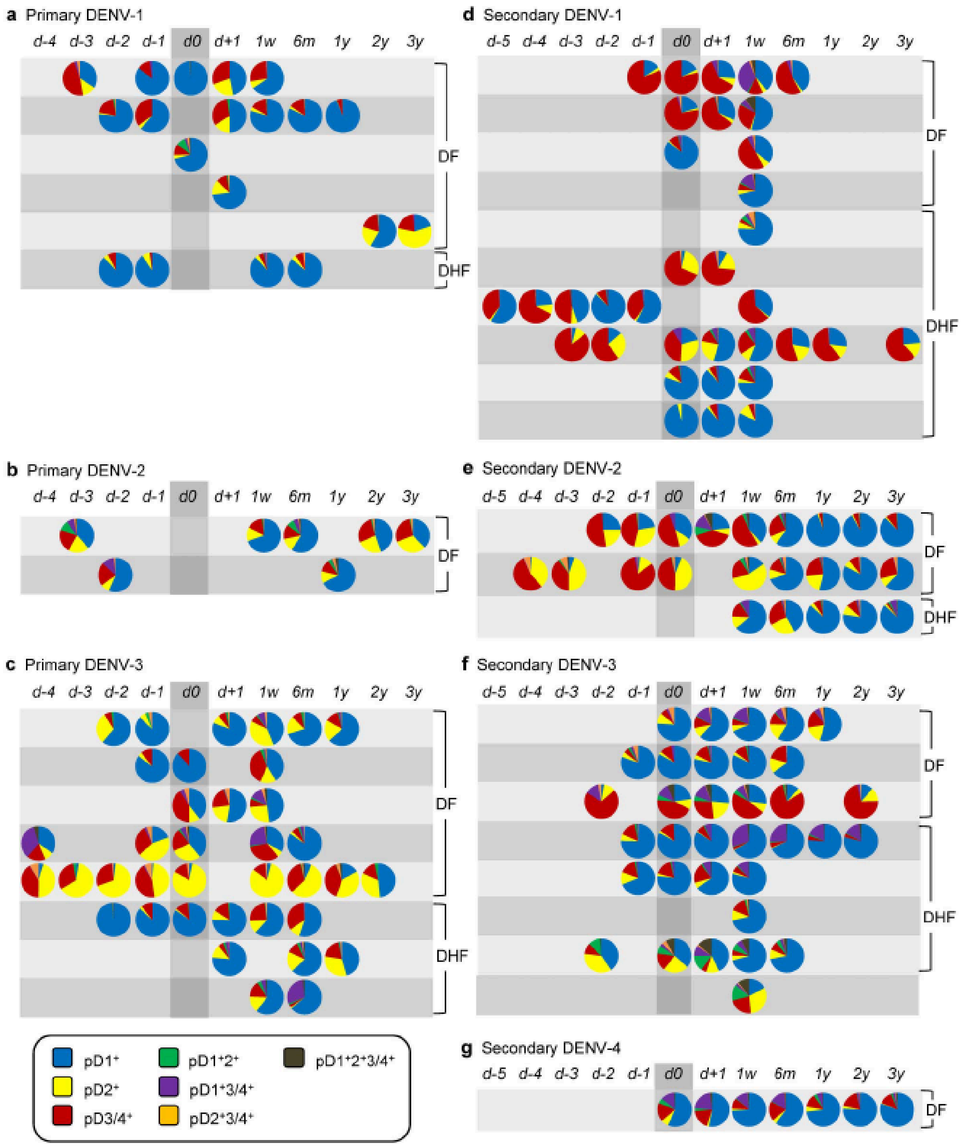

Rebuttal Figure 15. Tet+ CD8 T cells are highly serotype-cross-reactive in subjects with primary or secondary infection. The distribution of subpopulations within the whole population of A*11-$NS3_{133}$ Tet+ CD8+ T cells is depicted using pie charts. Tet+ samples from subjects with a (a–c) primary or (d–g) secondary infection with (a, d) DENV-1, (b, e) DENV-2, (c, f) DENV-3 or (g) DENV-4 are shown. No subjects with primary DENV-4 infection were available. Each row represents data from a single donor over time. Time points shown are relative to the day of defervescence (d0), d = day, w=week, m= month, y= year (Friberge et al, 2011)[4]

7. In addition, tet staining should be performed on HC to evaluate specificity of the staining (Figure S2 shows the FMO for the HC, so there a specific binding cannot be evaluated)

To further evaluate the specificity of tetramer staining beyond the FMO controls shown in Figure S2, we refer to prior validation studies of these tetramers and present additional control analyses performed in this study.

First, the DENV NS3 pHLA tetramers used in this study (GTS-HLA-A*11 and NYA-HLA-A*24 variants) have been extensively characterized in prior works[3,20] . These studies showed that Tet+ populations are detected in HLA-matched dengue-immune donors but are absent or minimal in HLA-matched dengue-naïve or HLA-mismatched controls (Rebuttal Figure 16). In addition, epitope specificity has been validated using epitope-specific CD8+ T-cell clones, competitive binding assays, structural pHLA-TCR analyses, and functional assays[3,8,20]. Collectively, these data support antigen-specific binding of these pHLA reagents, although low-level background cannot be entirely excluded, as is typical for any tetramer-based assay[36] .

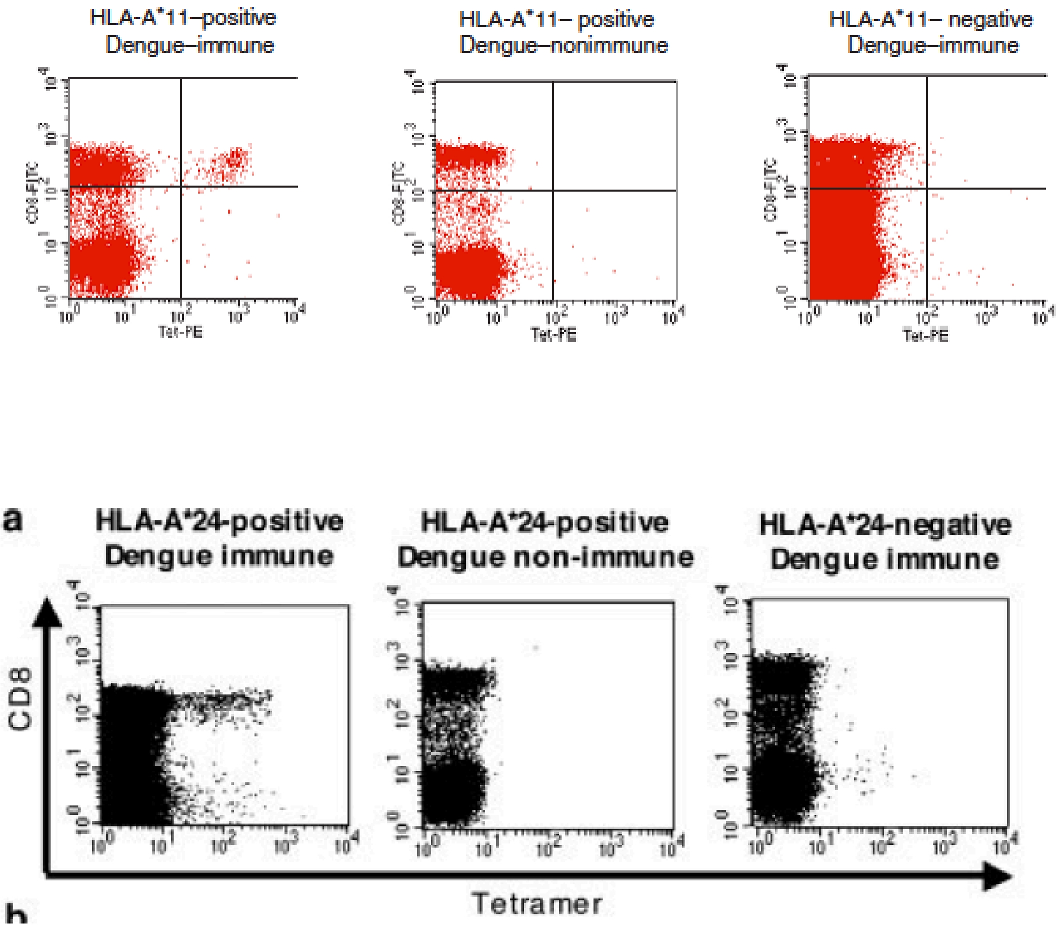

Rebuttal Figure 16. Representative flow cytometry plots demonstrating specificity of GTS–HLA-A*11 (above, Mongkolsapaya et al, 2003) and NYA–HLA-A*24 (below, Mongkolsapaya et al, 2006) pHLA tetramer staining. Clear Tet+ CD8+ T-cell populations are observed in HLA-matched dengue-immune donors, whereas minimal background staining is detected in HLA-matched dengue non-immune and HLA-mismatched dengue-immune controls. These comparisons support the specificity of tetramer binding.

Second, we reanalyzed tetramer staining data from the original sorting experiments, which included HLA-mismatched healthy controls alongside HLA-matched dengue patients. For both epitopes (GTS-HLA-A11 and NYA-HLA-A24), Tet+ events were rare and of low fluorescence intensity in the HLA-mismatched controls, whereas HLA-matched dengue patients displayed a distinct Tet+ CD8+ T-cell population (Rebuttal Figure 17A–B), consistent with HLA-restricted antigen-specific binding. We also incorporate these figures to Supplementary Figure 3a and b in revised manuscript

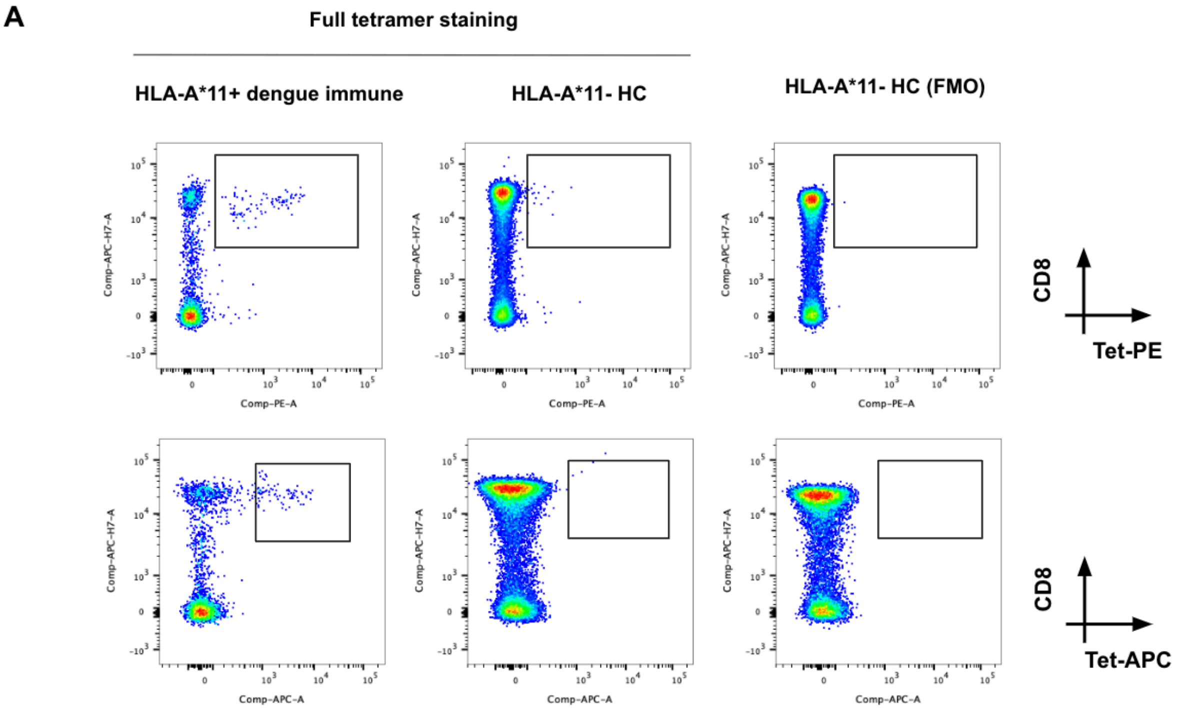

B

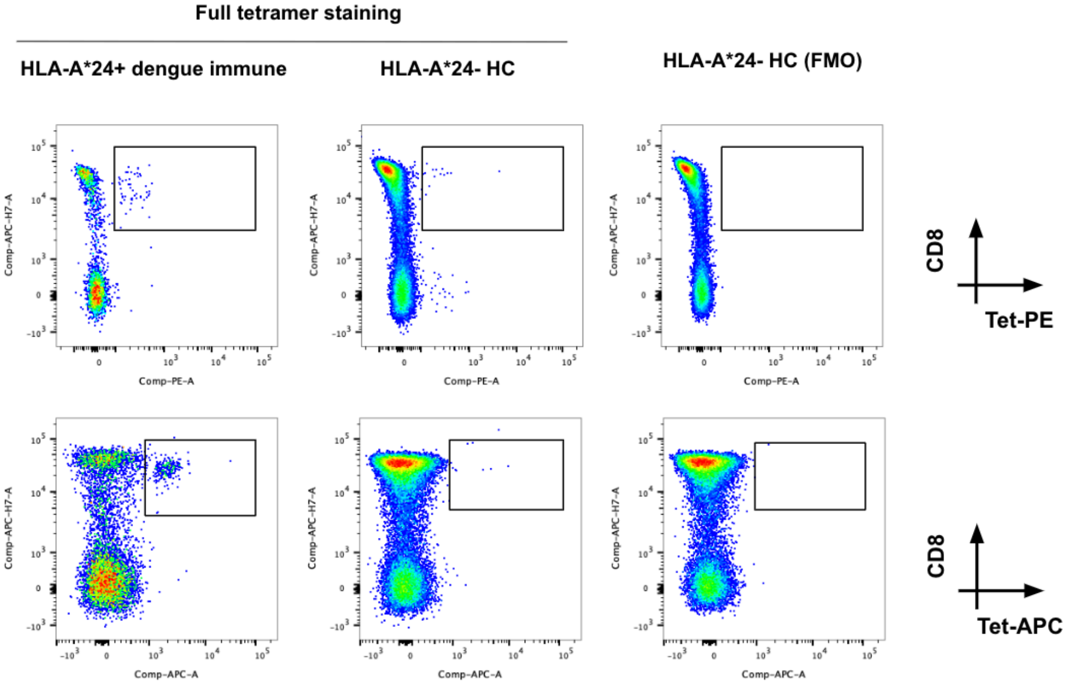

Rebuttal Figure 17. Tetramer specificity and gating strategy. Representative flow cytometry plots of Tet-PE (top) and Tet-APC (bottom) staining using GTS–HLA-A11 tetramers (A) and NYA–HLA-A24 tetramers (B). Boxed regions indicate Tet+ CD8+ T cells. A dengue-immune donor shows a distinct Tet+ CD8+ T cell population (left), whereas HLA-matched healthy controls show minimal background events (middle). Fluorescence-minus-one (FMO) controls (right) overlap with the tetramer-negative population observed in fully stained samples. Gating cutoffs were therefore established based on the FMO controls.

Third, we performed additional staining in HLA-matched healthy donors using PKI pretreatment to enhance TCR surface stability and tetramer binding[36]. Although PKI can increase overall tetramer signal and background, HLA-matched dengue patients still showed dominant Tet+ CD8+ T-cell populations compared with healthy controls (Rebuttal Figure 18). As expected in this dengue-endemic setting, some healthy donors exhibited low-frequency Tet+ events, likely reflecting prior exposure.

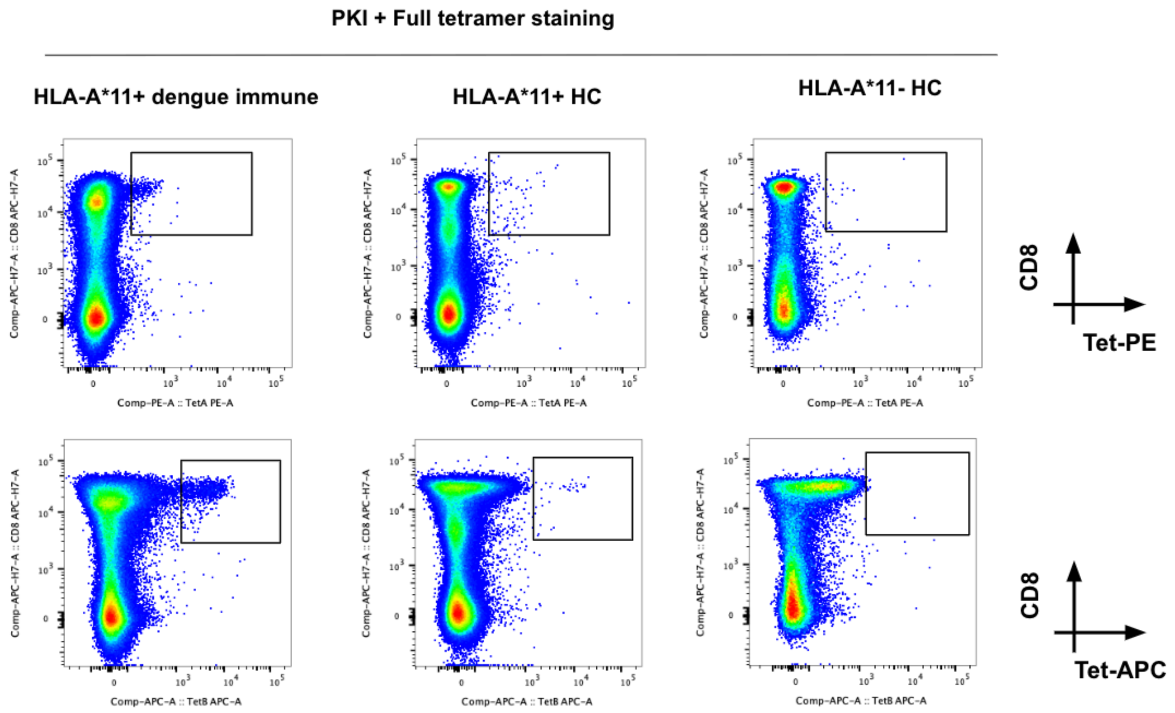

Rebuttal Figure 18. Tetramer specificity under PKI-enhanced staining. Representative flow cytometry plots of Tet-PE (top) and Tet-APC (bottom) with PKI pretreatment. Boxed regions indicate Tet+ CD8+ T cells. HLA-A*11+ dengue-immune donors show clearly separable Tet+ CD8+ T cell populations, whereas HLA-matched healthy controls and HLA-mismatched controls display only rare, weakly stained events.

Fourth, in a small number of samples, such as YH089 and IH006, the number of sorted Tet+ cells approached background levels. However, analysis of the inferred TCR sequences identified clonotypes that were also detected within expanded clonotype networks in the corresponding 10x single-cell dataset (Rebuttal Figure 19), supporting that these cells represent bona fide antigen-specific CD8+ T cells rather than nonspecific tetramer binding.

b

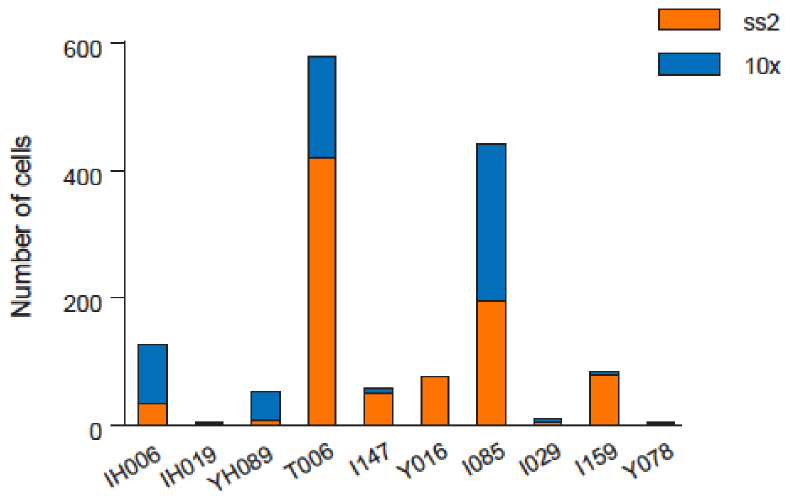

Rebuttal Figure 19. Integration of GTS-specific CD8+ T cells from matched HLA-A*11+ donors across two scRNA-seq technologies. Stack bar plot showing the number of GTS-specific CD8+ T cells derived from each donor, stratified by scRNA-seq technology (corresponding to Supplementary Figure 11b).

Taken together, prior validation of these pHLA tetramers, the minimal background observed in HLA-mismatched controls, the dominance of Tet+ populations in HLA-matched dengue patients under PKI-enhanced conditions, and the concordant clonotypic evidence from TCR analyses collectively support that the Tet+ populations identified here represent dengue-specific CD8+ T cells.

8. How sure are the authors that pHLA tetramer fluorescence intensity can be used as a proxy for TCR avidity? Is a higher MFI not associated with higher expression of TCR on the surface.

We thank the reviewer for raising this point regarding the interpretation of pHLA tetramer fluorescence intensity. We recognize that the term "TCR avidity" can be defined in different ways across studies. In this work, we use tetramer mean fluorescence intensity (MFI) as a relative proxy for TCR avidity under controlled staining conditions, consistent with prior studies where tetramer MFI has been used to reflect the effective strength of pMHC-TCR engagement at the cell surface[37-39].

We agree that higher tetramer MFI could, in principle, be influenced by higher surface TCR expression. To address this possibility, we examined TCR constant region (*TCRC*) expression across CD8+ T-cell subsets and did not observe a direct correspondence between inferred TCR abundance and tetramer MFI. For example, CX3CR1+ CD8+ T cells displayed relatively lower *TCRC* transcript levels yet exhibited higher tetramer MFI, whereas intermediate CD8+ T cells showed higher *TCRC* transcript levels but lower tetramer MFI. While transcript levels do not

provide a direct measurement of surface TCR protein, these observations argue against TCR abundance alone explaining the observed differences in tetramer intensity (Rebuttal Figure 20).

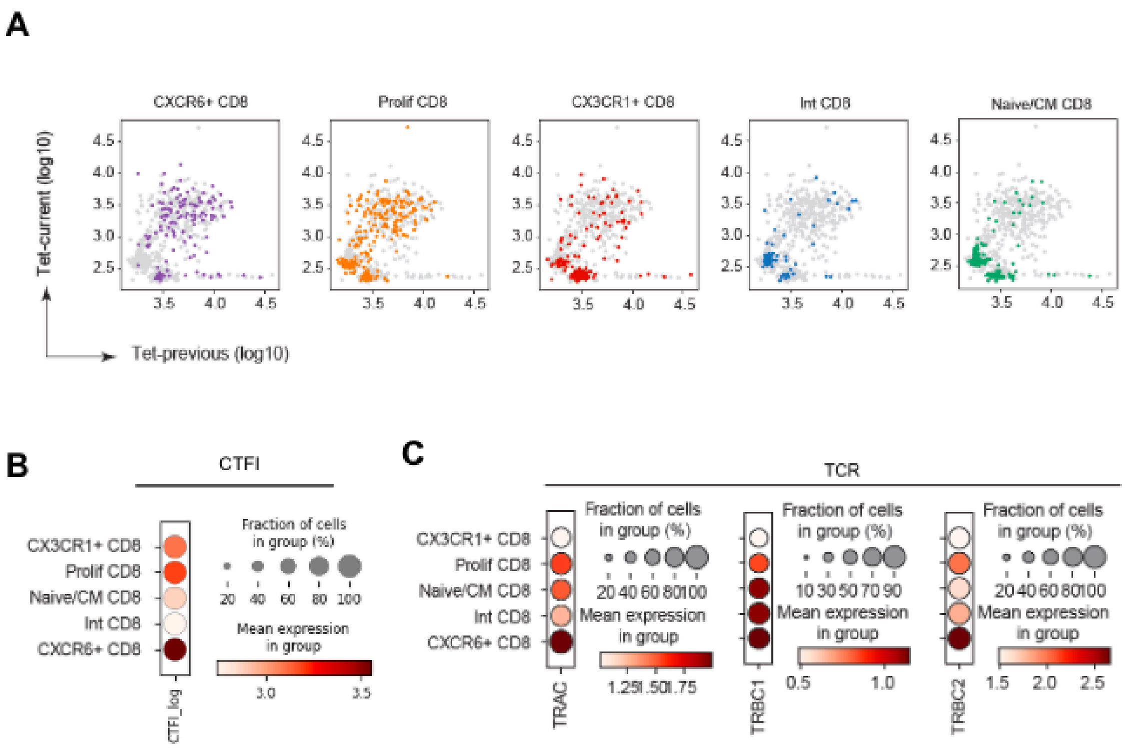

Rebuttal Figure 20. Relationship between tetramer fluorescence intensity (MFI)and TCR transcript abundance. A) Scatter plots showing MFI across CD8+ T-cell subsets (Figure 4a in the revised manuscript). (B) Composite tetramer fluorescence index (CTFI) across CD8+ T-cell subsets, calculated using the Euclidean norm of PE and APC signals, as described in the response to Reviewer 2. (C) Expression of TCR constant-region transcripts (TRAC, TRBC1, TRBC2) across CD8+ T-cell subsets (Figure 4e in the revised manuscript)

To avoid ambiguity, we have revised the manuscript to clarify that tetramer MFI is used here as a relative proxy for TCR avidity under controlled staining conditions.

Original Text (Result):
Line 163: Individual Tet+ cells were index-sorted into 96-well plates for subsequent Smart-seq2-based scRNA-seq (SS2), with fluorescent intensity as a proxy for TCR avidity.

Revised Text (Result):
Line 163: Individual Tet+ cells were index-sorted into 96-well plates for subsequent Smart-seq2-based scRNA-seq (SS2), with fluorescent intensity as a proxy for TCR avidity, as tetramer staining intensity has previously been shown to reflect the overall strength of TCR-pMHC interactions and CD8 co-receptor engagement[27-29].

Original Text (Result):
Line 293: Using pHLA tetramer fluorescence intensity as a proxy for TCR avidity,....

Revised Text (Result):
Line 293: Using pHLA tetramer fluorescence intensity as a proxy for TCR avidity, consistent with previous studies[27-29]

9. The authors mention themselves somewhere that T cell activation leads to downregulation of TCR and hence less pHLA+ cells. Do the authors have any data to support this interpretation?

The concept of activation-induced TCR downregulation is supported both by the literature and by observations, as discussed in the original manuscript in the Discussion. In multiple samples in this study, particularly during the acute phase, the number of tetramer-captured and FACS-sorted DENV-specific CD8+ T cells was relatively low. This observation was interpreted in light of the known phenomenon that strong TCR activation can lead to transient TCR downregulation and reduced pMHC tetramer binding[40] . To address this possibility, we integrated the tetramer-sorted data with total CD8+ T cells from a partially matched droplet-based dataset. In this analysis, we identified a substantially higher number of cells within the same clonotype networks, defined by shared V(D)J gene usage and CDR3 sequences, than were detected by tetramer sorting alone (Supplementary Figures 11a-b in revised manuscript). The results suggest that tetramer-based isolation may underestimate antigen-specific populations during periods of strong activation.

This interpretation was also consistent with the transcriptional profiles of the most activated effector population (CX3CR1+ CD8+ T cells). This population showed strong enrichment of activation- and TCR-signaling-associated transcriptional programs, including NFAT/TOX-related signatures, together with reduced expression of TCR constant-region transcripts (*TRAC*, *TRBC1*, *TRBC2*) compared with less activated subsets (Fig. 4e). Although transcript abundance does not directly reflect surface TCR protein levels, this pattern is consistent with activation-associated TCR downregulation reported in prior studies[40,41].

Finally, we directly tested whether TCR internalization could influence tetramer detection. PKI treatment, which inhibits activation-induced TCR internalization[36,42], resulted in enrichment of Tet+ CD8+ T cells in convalescence samples (Rebuttal Figure 21). This observation is consistent with the interpretation that activation-induced TCR downregulation reduces detectable pHLA tetramer binding during peak activation.

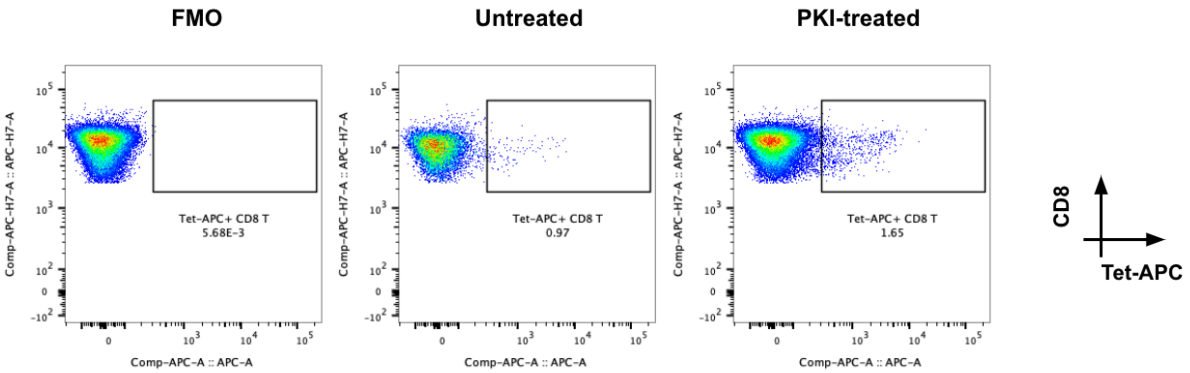

Rebuttal Figure 21. Effect of PKI treatment on tetramer detection. Representative flow cytometry plots showing GTS tetramer staining of CD8+ T cells under FMO control, untreated, and PKI-treated conditions in a convalescent dengue sample (F1). Percentages indicate Tet-positive CD8+ T cells among total CD8+ T cells.

Taken together, prior literature, activation-associated transcriptional signatures, clonotype-based recovery of inferred antigen-specific cells in the total PBMC dataset, and PKI rescue experiments support the interpretation that TCR activation during acute infection may lead to TCR downregulation, resulting in reduced detection of pHLA Tet+ CD8+ T cells.

10. In several instances, CD3CR1+ CD8+ T cells are called "potentially associated with vascular leakage in DHF". What is the rationale for this?

The rationale for this interpretation is based on both observations from our dataset and established CX3CR1-CX3CL1 endothelial biology. CX3CR1+ cytotoxic CD8+ T cells were preferentially enriched in DHF compared with DF/AD during the acute phase (Fig. 3a-b), indicating an association with severe disease states in which vascular leakage occurs. This subset also exhibits a strong cytotoxic and inflammatory effector program, including high expression of *GZMB*, *PRF1*, *GNLY*, and inflammatory chemokines such as *CCL3* and *CCL4* (Fig. 2d), consistent with the capacity to amplify local tissue inflammation. In addition, CX3CR1 mediates interactions with its ligand CX3CL1 (fractalkine), which is expressed by activated endothelial cells and has been implicated in the recruitment and retention of CX3CR1+ effector T cells at sites of vascular inflammation[43-45].

Consistent with this biology, the abundance and clonal expansion of CX3CR1+ CD8+ T cells in our dataset correlate with inflammatory cytokines previously linked to severe dengue and vascular permeability (Fig. 6d). Together, these observations support describing this subset as potentially associated with vascular inflammation relevant to DHF pathophysiology, while not implying a direct causal role in vascular leakage without further evidence.

11. On how many Tet+ CD8+ T cells were the scRNAseq analysis done for each individual? It would be good to add these numbers, as I suspect these are very small populations.

Similarly, how many cells of each individual was the TCR repertoire analysis done? Was this not biased towards a few individuals with more cells?

The exact numbers of Tet+ CD8+ T cells subjected to scRNA-seq for each donor, as well as those analyzed in the TCR repertoire analyses after inferred data integration, are now provided in Rebuttal Table 6-7, which have been incorporated into the revised manuscript as Supplementary Tables 4–5. As expected, these populations are small in some individuals, reflecting the intrinsic rarity of antigen-specific CD8+ T cells during acute dengue infection.

Rebuttal Table 6. Number of antigen-specific CD8+ T cells included in TCR repertoire analysis by donor.

donor_id	severity	HLA	clinical_phase	cell_count
IH006	AD	A*11	Acute	51
IH023	AD	A*24	Acute	14
YH089	AD	A*11	Acute	9
I109	DF	A*24	Acute	12
I147	DF	A*11	Acute	3
I171	DF	A*24	Acute	154
T006	DF	A*11	Acute	382
T010	DF	A*24	Acute	10
Y029	DF	A*24	Acute	70
I029	DHF	A*11	Acute	9
I075	DHF	A*24	Acute	22
I085	DHF	A*11	Acute	38
I159	DHF	A*11	Acute	1
Y078	DHF	A*24 & A*11	Acute	1
I109	DF	A*24	Convalescence	56
I147	DF	A*11	Convalescence	54
I171	DF	A*24	Convalescence	217
T006	DF	A*11	Convalescence	101
T010	DF	A*24	Convalescence	9
Y016	DF	A*24 & A*11	Convalescence	161
Y029	DF	A*24	Convalescence	70
I075	DHF	A*24	Convalescence	23
I085	DHF	A*11	Convalescence	243
I159	DHF	A*11	Convalescence	95

Rebuttal Table 7. Number of sorted and inferred DENV-specific CD8+ T cells included in TCR repertoire analysis across HLA-A*11+ donors.

donor_id	severity	clinical_phase	cell count		
			Sorted CD8+ T cell(SS2)	inferred DENV-specific CD8+ T cell (10x)	Total cell in analysis
IH006	AD	Acute	34	93	127
IH019	AD	Acute	3	1	4
YH089	AD	Acute	8	44	52
I147	DF	Acute	6	9	15
I147	DF	Convalescence	43	0	43
T006	DF	Acute	344	160	504
T006	DF	Convalescence	76	0	76
Y016	DF	Convalescence	76	0	76
I029	DHF	Acute	6	4	10
I085	DHF	Acute	30	244	274
I085	DHF	Convalescence	166	0	166
I159	DHF	Acute	0	5	5
I159	DHF	Convalescence	80	0	80
Y078	DHF	Acute	3	2	5
Total			875	562	1437

We acknowledge that variation in recovered Tet+ cell numbers across donors could introduce bias if analyses were based on pooled cell counts in each severity group. To address this, quantitative comparisons were conducted at the donor level rather than based on raw cell counts, and non-parametric, donor-aware statistical approaches were applied. Findings that did not reach statistical significance were conservatively described as trends.

For TCR repertoire analyses, we further implemented statistical models that explicitly account for donor identity and differences in sampling depth (**as detailed in our response to Reviewer 2**). This framework reduces the influence of unequal Tet+ cell numbers across individuals and helps ensure that observed differences in TCR and TCR repertoire features are not driven by a single or a few donors contributing disproportionately more cells.

To improve transparency, per-donor cell numbers are now provided in the Supplementary Information and the manuscript has been revised to clarify how donor-level analyses account for differences in recovered cell numbers across individuals.

Added Text (Discussion):
Line 552:…Where applicable, quantitative comparisons were performed at the donor level to account for differences in recovered cell numbers across individuals.

12. Important recent publications in the field, such as Gregorova et al, Nat Comm 2025 and Gonnella et al, 2025 should be discussed.

We thank the reviewer for highlighting these recent studies. The manuscript has been revised to incorporate and discuss Gregorova et al. (Nat. Commun. 2025) and Gonnella et al. (2025), which reported early CD8+ T-cell dysregulation in severe dengue based on analyses of total circulating or peptide-stimulated CD8+ T-cell populations. The revised text now summarizes

these findings and clarifies how our antigen-specific tetramer-based analysis complements these observations.

Added Text (Introduction):
Line 118: Consistent with recent studies analyzing total circulating or peptide-stimulated CD8+ T-cell populations, early CD8+ T-cell dysregulation has been reported in SD, including the expansion of activated CD8+ TEM cells with inhibitory features and altered functional profiles

Original Text (Introduction):
Line 122: This robust immune response suggested a potential protective role for CD8+ T cells.

Revised Text (Introduction):
Line 120: These observations suggest that CD8+ T-cell responses characterized by enhanced effector functions and reduced dysfunction, as observed in AD, may contribute to protective immunity.

References

1 Weiskopf, D. *et al.* Comprehensive analysis of dengue virus-specific responses supports an HLA-linked protective role for CD8+ T cells. *Proc Natl Acad Sci U S A* **110**, E2046-2053, doi:10.1073/pnas.1305227110 (2013).
2 Duangchinda, T. *et al.* Immunodominant T-cell responses to dengue virus NS3 are associated with DHF. *Proc Natl Acad Sci U S A* **107**, 16922-16927, doi:10.1073/pnas.1010867107 (2010).
3 Mongkolsapaya, J. *et al.* Original antigenic sin and apoptosis in the pathogenesis of dengue hemorrhagic fever. *Nature Medicine* **9**, 921-927, doi:10.1038/nm887 (2003).
4 Friberg, H. *et al.* Cross-reactivity and expansion of dengue-specific T cells during acute primary and secondary infections in humans. *Sci Rep* **1**, 51, doi:10.1038/srep00051 (2011).
5 Friberg, H. *et al.* Memory CD8+ T cells from naturally acquired primary dengue virus infection are highly cross-reactive. *Immunol Cell Biol* **89**, 122-129, doi:10.1038/icb.2010.61 (2011).
6 Townsley, E. *et al.* Distinct activation phenotype of a highly conserved novel HLA-B57-restricted epitope during dengue virus infection. *Immunology* **141**, 27-38, doi:10.1111/imm.12161 (2014).
7 Rivino, L. *et al.* Virus-specific T lymphocytes home to the skin during natural dengue infection. *Science Translational Medicine* **7**, 278ra235-278ra235, doi:10.1126/scitranslmed.aaa0526 (2015).
8 Culshaw, A. *et al.* Germline bias dictates cross-serotype reactivity in a common dengue-virus-specific CD8(+) T cell response. *Nat Immunol* **18**, 1228-1237, doi:10.1038/ni.3850 (2017).
9 Chng, M. H. Y. *et al.* Large-Scale HLA Tetramer Tracking of T Cells during Dengue Infection Reveals Broad Acute Activation and Differentiation into Two Memory Cell Fates. *Immunity* **51**, 1119-1135 e1115, doi:10.1016/j.immuni.2019.10.007 (2019).
10 Nguyen, T. H. O. *et al.* CD8(+) T cells specific for an immunodominant SARS-CoV-2 nucleocapsid epitope display high naive precursor frequency and TCR promiscuity. *Immunity* **54**, 1066-1082 e1065, doi:10.1016/j.immuni.2021.04.009 (2021).

11 Rowntree, L. C. *et al.* SARS-CoV-2-specific CD8(+) T cells from people with long COVID establish and maintain effector phenotype and key TCR signatures over 2 years. *Proc Natl Acad Sci U S A* **121**, e2411428121, doi:10.1073/pnas.2411428121 (2024).

12 Saini, S. K. *et al.* SARS-CoV-2 genome-wide T cell epitope mapping reveals immunodominance and substantial CD8(+) T cell activation in COVID-19 patients. *Sci Immunol* **6**, doi:10.1126/sciimmunol.abf7550 (2021).

13 Koutsakos, M. *et al.* Human CD8(+) T cell cross-reactivity across influenza A, B and C viruses. *Nat Immunol* **20**, 613-625, doi:10.1038/s41590-019-0320-6 (2019).

14 van de Sandt, C. E. *et al.* Challenging immunodominance of influenza-specific CD8(+) T cell responses restricted by the risk-associated HLA-A*68:01 allomorph. *Nat Commun* **10**, 5579, doi:10.1038/s41467-019-13346-4 (2019).

15 Satapornpong, P. *et al.* Genetic Diversity of HLA Class I and Class II Alleles in Thai Populations: Contribution to Genotype-Guided Therapeutics. *Front Pharmacol* **11**, 78, doi:10.3389/fphar.2020.00078 (2020).

16 Vaughan, K., Greenbaum, J., Blythe, M., Peters, B. & Sette, A. Meta-analysis of all immune epitope data in the Flavivirus genus: inventory of current immune epitope data status in the context of virus immunity and immunopathology. *Viral Immunol* **23**, 259-284, doi:10.1089/vim.2010.0006 (2010).

17 Roth, C. *et al.* A Modified mRNA Vaccine Targeting Immunodominant NS Epitopes Protects Against Dengue Virus Infection in HLA Class I Transgenic Mice. *Front Immunol* **10**, 1424, doi:10.3389/fimmu.2019.01424 (2019).

18 Loke, H. *et al.* Strong HLA class I--restricted T cell responses in dengue hemorrhagic fever: a double-edged sword? *J Infect Dis* **184**, 1369-1373, doi:10.1086/324320 (2001).

19 Simmons, C. P. *et al.* Early T-cell responses to dengue virus epitopes in Vietnamese adults with secondary dengue virus infections. *J Virol* **79**, 5665-5675, doi:10.1128/JVI.79.9.5665-5675.2005 (2005).

20 Mongkolsapaya, J. *et al.* T cell responses in dengue hemorrhagic fever: are cross-reactive T cells suboptimal? *J Immunol* **176**, 3821-3829, doi:10.4049/jimmunol.176.6.3821 (2006).

21 Lim, M. Q. *et al.* Cross-Reactivity and Anti-viral Function of Dengue Capsid and NS3-Specific Memory T Cells Toward Zika Virus. *Front Immunol* **9**, 2225, doi:10.3389/fimmu.2018.02225 (2018).

22 WHO. Guidelines for plaque reduction neutralization testing of human antibodies to dengue viruses. (2007).

23 Roehrig, J. T., Hombach, J. & Barrett, A. D. Guidelines for Plaque-Reduction Neutralization Testing of Human Antibodies to Dengue Viruses. *Viral Immunol* **21**, 123-132, doi:10.1089/vim.2008.0007 (2008).

24 Puschnik, A. *et al.* Correlation between dengue-specific neutralizing antibodies and serum avidity in primary and secondary dengue virus 3 natural infections in humans. *PLoS Negl Trop Dis* **7**, e2274, doi:10.1371/journal.pntd.0002274 (2013).

25 Capao, A. *et al.* Impact in the humoral and cellular immune response to SARS-CoV-2 variants after primary vaccination with AZD1222/COVISHIELD protocol in healthy adults. *Vaccine* **50**, 126785, doi:10.1016/j.vaccine.2025.126785 (2025).

26 de Teive, E. A. A. F. *et al.* High frequency of pre-existing neutralizing antibody responses in patients with dengue during an outbreak in Central Brazil. *BMC Infect Dis* **16**, 546, doi:10.1186/s12879-016-1867-6 (2016).

27 Katzelnick, L. C. *et al.* Antibody-dependent enhancement of severe dengue disease in humans. *Science* **358**, 929-932, doi:10.1126/science.aan6836 (2017).

28 Matangkasombut, P. *et al.* Dengue viremia kinetics in asymptomatic and symptomatic infection. *Int J Infect Dis* **101**, 90-97, doi:10.1016/j.ijid.2020.09.1446 (2020).

29 Indu, P. S. *et al.* The burden of dengue and force of infection among children in Kerala, India; seroprevalence estimates from Government of Kerala-WHO Dengue study. *Lancet Reg Health Southeast Asia* **22**, 100337, doi:10.1016/j.lansea.2023.100337 (2024).
30 Nindee, J. *et al.* Seroepidemiological surveillance of neutralizing antibodies against dengue virus serotypes among healthy adults in Bangkok, Thailand. *One Health* **21**, 101202, doi:10.1016/j.onehlt.2025.101202 (2025).
31 Sridhar, S. *et al.* Cellular immune correlates of protection against symptomatic pandemic influenza. *Nat Med* **19**, 1305-1312, doi:10.1038/nm.3350 (2013).
32 Sungnak, W. *et al.* Distinct systemic immune responses in asymptomatic and symptomatic dengue virus infection. *Sci Transl Med* **17**, eads5932, doi:10.1126/scitranslmed.ads5932 (2025).
33 Kiselev, V. Y., Andrews, T. S. & Hemberg, M. Publisher Correction: Challenges in unsupervised clustering of single-cell RNA-seq data. *Nat Rev Genet* **20**, 310, doi:10.1038/s41576-019-0095-5 (2019).
34 Luecken, M. D. & Theis, F. J. Current best practices in single-cell RNA-seq analysis: a tutorial. *Mol Syst Biol* **15**, e8746, doi:10.15252/msb.20188746 (2019).
35 Oberg, A. L. & Mahoney, D. W. Linear mixed effects models. *Methods Mol Biol* **404**, 213-234, doi:10.1007/978-1-59745-530-5_11 (2007).
36 Dolton, G. *et al.* More tricks with tetramers: a practical guide to staining T cells with peptide-MHC multimers. *Immunology* **146**, 11-22, doi:10.1111/imm.12499 (2015).
37 Mallone, R. *et al.* Functional avidity directs T-cell fate in autoreactive CD4+ T cells. *Blood* **106**, 2798-2805, doi:10.1182/blood-2004-12-4848 (2005).
38 Laugel, B. *et al.* Different T cell receptor affinity thresholds and CD8 coreceptor dependence govern cytotoxic T lymphocyte activation and tetramer binding properties. *J Biol Chem* **282**, 23799-23810, doi:10.1074/jbc.M700976200 (2007).
39 Zhong, S. *et al.* T-cell receptor affinity and avidity defines antitumor response and autoimmunity in T-cell immunotherapy. *Proc Natl Acad Sci U S A* **110**, 6973-6978, doi:10.1073/pnas.1221609110 (2013).
40 Gallegos, A. M. *et al.* Control of T cell antigen reactivity via programmed TCR downregulation. *Nat Immunol* **17**, 379-386, doi:10.1038/ni.3386 (2016).
41 Cemerski, S. *et al.* The balance between T cell receptor signaling and degradation at the center of the immunological synapse is determined by antigen quality. *Immunity* **29**, 414-422, doi:10.1016/j.immuni.2008.06.014 (2008).
42 Lissina, A. *et al.* Protein kinase inhibitors substantially improve the physical detection of T-cells with peptide-MHC tetramers. *J Immunol Methods* **340**, 11-24, doi:10.1016/j.jim.2008.09.014 (2009).
43 Panigrahi, S. *et al.* CX3CL1 and IL-15 Promote CD8 T cell chemoattraction in HIV and in atherosclerosis. *PLoS Pathog* **16**, e1008885, doi:10.1371/journal.ppat.1008885 (2020).
44 Su, X., O'Hare, K. A. & Freeman, M. L. Mechanisms and implications of vascular-homing CD8 T cells in atherosclerosis. *NPJ Cardiovasc Health* **2**, 19, doi:10.1038/s44325-025-00056-8 (2025).
45 von Vietinghoff, S. & Kurts, C. Regulation and function of CX3CR1 and its ligand CX3CL1 in kidney disease. *Cell Tissue Res* **385**, 335-344, doi:10.1007/s00441-021-03473-0 (2021).

Reviewer #1 (Remarks to the Author):
This submission has been significantly improved in revision and most of my prior suggestions/comments have been adequately addressed. However, there remains some points that need to be addressed.
We thank the reviewer for the positive assessment and for recognizing the improvements in the revised manuscript.

1. While the authors have clearly justified their use of NS3-derived peptides for the identification and isolation of DENV-specific T cells, the point remains that it is unclear what fraction of the total DENV-specific T cell response this population represents. Even in the papers cited by the authors to justify their exclusive use of these NS3-derived peptides for this analysis, significant responses to antigens other than NS3 are observed, with the difference between the subdominant and immunodominant antigens differing only by 1-2 fold. The additional bioinformatic analysis provided in the rebuttal doesn't directly alleviate this concern, as up to 98% of expanded/activated T cell clones do not appear to express GTS-specific TCRs. I understand the limitations and challenges associated with this analysis approach - and wholeheartedly agree with the authors that this is the best approach given the limitations of other assays - but I would appreciate the authors being more precise in how the data are framed/presented. If the authors aren't able to clearly define what fraction of the total DENV-specific T cell responses these TET-positive CD8 T cells represent, I would recommend that the conclusions and data clearly state "DENV NS3 specific" or "GTS-specific" T cell responses rather than "DENV-specific T cell responses.

We acknowledge that NS3-derived epitopes represent only a subset of the total DENV-specific CD8+ T cell response, and that contributions from other viral antigens are not captured. As discussed in our previous response, however, these epitopes are among the most well-characterized and immunodominant targets in human dengue infection. We also acknowledge that we cannot define the proportion of the total response represented by the tetramer-positive population.

In response to the reviewer's suggestion, we have revised the manuscript to more precisely describe these populations. Specifically, we now refer to "DENV NS3-specific" or "GTS-specific" CD8+ T cell responses, rather than broadly describing them as "DENV-specific", where appropriate.

2. There remains some cognitive dissonance regarding the relationship between antibody titers, CD8 T cell responses, and inferred infection history. The authors maintain that one of the primary objectives of this analysis is to compare the T cell responses directed against historic and current DENV antigens, with the historic virus inferred by serology. However, in their rebuttal, the authors acknowledge that "in some donors we observed relatively low PRNT titers to certain serotypes despite detectable Tet+ CD8+ T-cell responses. This further supports the concept that neutralizing antibody and CD8+ T-cell responses do not necessarily correlate perfectly and may reflect differential or biased immune recall within distinct immune compartments". Given this discordance, why is prior infection history pinned to the specificity of the antibody responses rather than the T cell response? In light of this - and the clearly acknowledged challenges associated with inferring infection history based on serology - I would strongly recommend paraphrasing/repositioning "inferred prior infection" or "previous infection" to something along the lines of "Predominant seroreactivity" or "putative serologic exposure". Acknowledging these limitations in no way detracts from the

importance or impact of the observations described in this study, but more precisely reflects the data.

First, as observed in our dataset, neutralizing antibody titers and CD8+T cell responses show broad but incomplete concordance. While instances of discordance are present, we argue that these do not preclude the use of serology-based inference. Rather, they reflect differences between humoral and cellular immune compartments. We therefore do not consider this level of discordance sufficient to dismiss the aspect of infection history altogether, as doing so would limit the ability to contextualize our findings within the extensive body of dengue immunology literature, where serology-based frameworks have contributed to the current understanding of dengue immunology in the context of infection history.

Second, in response to the reviewer's concern, we have taken a careful and explicit approach to terminology. Building on the previous revision, we have used the term "serologically inferred dominant previously infecting DENV serotype", which we define as the non-current serotype with the highest PRNT titer. This terminology was deliberately constructed to reflect that the classification is (i) based on serological inference, (ii) restricted to the dominant non-current serotype, and (iii) interpreted in the context of prior infection, while not representing definitive virological confirmation. As a concrete action, we have standardized this terminology throughout the manuscript, applying it consistently and removing alternative phrasings to avoid ambiguity.

We have also already explicitly stated in the Discussion that serology-based inference of infection history is not definitive and may not fully align with T cell responses. We have emphasized that our classification reflects serologically inferred exposure rather than confirmed infection history and acknowledge this as a limitation of the study. We have also further noted that future studies incorporating longitudinal sampling and orthogonal approaches will be required to more precisely define infection history and better resolve concordance and discordance between humoral and cellular immune responses .

Overall, rather than removing the infection history framework, we have taken a balanced approach by retaining it in a carefully defined and qualified form, while clearly articulating its limitations and the need for further investigation. We argue that this approach helps maintain technical precision while still supporting interpretation of our findings in the context of the existing body of dengue immunology literature.

Reviewer #2 (Remarks to the Author):
I appreciate the effort the authors have made in revising the manuscript and responding to the previous comments. The figures are clearer, and some additional statistical analyses have been added.
We thank the reviewer for the positive feedback and for acknowledging the improvements in the revised manuscript.

1. That said, several major concerns remain. First, the cohort is still small and heterogeneous, which limits statistical power, and many interpretations feel overextended relative to the data.

We acknowledge that the cohort size is limited, as explicitly discussed in the Discussion. Nonetheless, we respectfully disagree with the characterization that the interpretations are overextended relative to the data. Key findings are statistically significant and show consistent effect directions across donors, with statistical frameworks that include donor-level aggregation, appropriate non-parametric testing, mixed-effects modeling to account for inter-donor variability, supporting the robustness of the observed effects despite the cohort size. Moreover, some of the key findings are largely consistent across multiple analytical layers, including integration with independent 10x datasets and orthogonal validation by flow cytometry.

Together, these results indicate that the key conclusions are directly supported by statistically significant and consistent effects despite the limited cohort size.

2. My primary concern—that most figures remain largely descriptive with insufficient statistical evaluation persists. I recognize that sampling from this patient population is challenging, but the main conclusions of the manuscript rely on observations that are not fully corroborated.

In the revised manuscript, all key findings have been evaluated using appropriate donor-level statistical analyses, which demonstrate statistically significant differences and form the basis of our main conclusions.

To reiterate, we bring up the details described in our previous response to this point, figure by figure, again below:

Figure 1 (flow cytometry)

Figure 1 is intended to provide an overview of the heterogeneity of dengue-specific CD8+ T-cell reactivity across disease severities and time points, rather than to test predefined group differences. Nonetheless, we performed statistical analyses and did not observe significant differences in dengue-specific CD8+ T-cell frequencies or epitope reactivity across disease severity or over the time course (Rebuttal Figure. 7).

Given the limited cohort size and donor heterogeneity, we therefore deliberately avoided overinterpreting these distributions. The absence of differences at the level of epitope reactivity warranted us to investigate whether heterogeneity instead emerged at the transcriptional level at single-cell resolution. Indeed, our subsequent analyses revealed clear differences in dengue-specific CD8+ T-cell types/states across disease severities (Fig. 3).

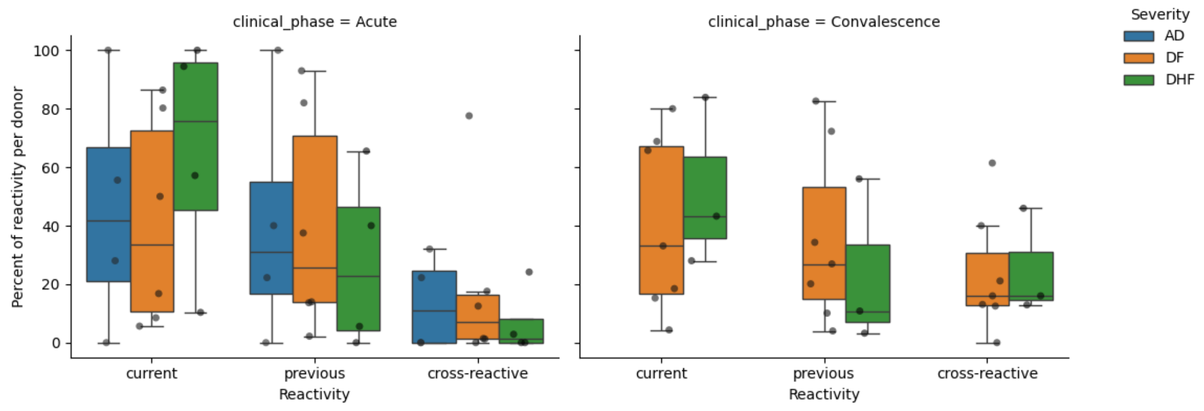

Rebuttal Figure 7. Flow cytometry analysis of dengue-specific CD8+ T-cell epitope reactivity (current, previous, cross-reactive) across severity groups during acute and convalescent phases. No significant differences in overall epitope frequencies were observed across severity or time points.

Figures 2-3 (single-cell clustering and differential cell type abundance analysis)

The clustering in Figure 2 follows a standard unsupervised scRNA-seq workflow aimed at identifying transcriptionally defined CD8+ T-cell subsets/states rather than testing differential abundance[33,34].

Because individual cells are not statistically independent within donors, downstream quantitative comparisons were performed at the donor level after cell-type assignment.

Specifically, after defining cell types, we assess cell-type abundance across disease severity at the donor level (Fig. 3a) and perform non-parametric statistical testing using individual donors as the unit of analysis (Fig. 3b), thereby avoiding inflated significance from a large number of cell counts.

While Figures 4 and 5 initially emphasized descriptive visualization of TCR features and clonality patterns, we agree that formal statistical testing is important to determine whether the observed trends reflect true biological differences rather than sampling variability. We therefore conducted the following additional analyses.

Figure 4 (tetramer fluorescence intensity)

Figure 4 links quantitative tetramer fluorescence intensity to defined CD8+ T-cell phenotypes. To avoid bias toward a single fluorochrome, we first integrated PE-A and APC-A tetramer signals into a composite tetramer fluorescence index (CTFI), calculated as the Euclidean norm:

$$CTFI = \sqrt{(PE - A)2 + (APC - A)2}$$

This metric captures overall tetramer staining intensity across channels and provides a more robust measure of binding strength.

After filtering donor–cluster combinations with fewer than four cells, differences in tetramer fluorescence intensity were evaluated using donor-aware linear mixed-effects regression models[35] , with cluster identity as a fixed effect and donor as a random effect.

The model was specified as:

$$CTFI_{logij} = \beta_0 + \beta_k(\text{Cell cluster}_k) + u_j + \varepsilon_{ij}$$

where

- i indexes individual cells
- j indexes donors.
- Leiden cluster was included as a fixed effect
- Donor ID was included as a random intercept.
- The random effect $u_j \sim N(0, \sigma^2_{donor})$ accounts for inter-donor variation
- $\varepsilon_{ij} \sim N(0, \sigma^2)$ represents residual error.

Mixed-effects modeling demonstrated that CX3CR1 CD8 exhibited significantly higher composite tetramer fluorescence compared to Prolif CD8, Naive/CM CD8, and Int CD8, (Prolif CD8 vs CX3CR1 CD8: $p < 0.001$; Naive/CM vs CX3CR1 CD8: $p = 0.002$; Int CD8 vs CX3CR1 CD8: $p = 0.023$). In contrast, CXCR6 CD8 did not differ significantly from CX3CR1 CD8 ($p = 0.506$) (Rebuttal Figure. 8). Importantly, these differences remained significant after accounting for donor-level variability, indicating that the observed phenotype-specific differences in tetramer staining intensity are not driven by unequal sampling across donors.

Together, these statistically significant differences indicate that CD8+ T-cell clusters defined by transcriptional profiles also exhibit systematic differences in tetramer staining intensity, supporting the existence of distinct TCR-associated phenotypes. This result has now been incorporated into the Results section and Supplementary Figure 6 in the revised manuscript.

Original Text (Result):
Line 296: …we found that during acute infection, high-avidity CD8+ T cells were enriched in CXCR6+ and, to a lesser extent, Prolif CD8 and CX3CR1+ CD8 subsets, while Naive/CM and Int CD8 subsets exhibited lower avidity (Fig. 4a).

Revised Text (Result):
Line 293: …we found that during acute infection, high-avidity CD8+ T cells were significantly enriched in CXCR6+ and CX3CR1+ CD8 subsets, compared to other subsets (Fig. 4a, Supplementary Figure 6)

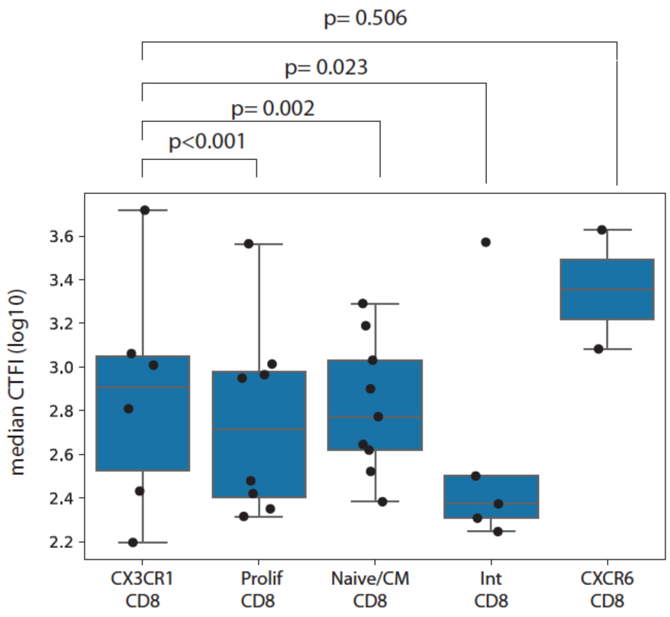

Rebuttal Figure 8. Donor-level median composite tetramer fluorescence index across CD8+ T-cell transcriptional clusters. Each dot represents one donor; box plots summarize cluster-level distributions. Mixed-effects modeling accounting for donor variability confirmed significant differences in tetramer staining intensity across clusters.

Rebuttal Table 4 Linear mixed-effects regression model for log-transformed composite tetramer fluorescence index

Cell cluster (reference = CX3CR1+ CD8)	Estimate (β)	Std. Error	z value	p-value	95% Confidence Interval
**Intercept (CX3CR1+ CD8)**	2.856	0.098	29.065	<0.001	2.663 to 3.049
Proliferating CD8	-0.125	0.033	-3.813	<0.001	-0.190 to -0.061
Naive/CM CD8	-0.117	0.038	-3.043	0.002	-0.192 to -0.042
Intermediate CD8	-0.106	0.047	-2.272	0.023	-0.198 to -0.015
CXCR6+ CD8	-0.027	0.040	-0.664	0.506	-0.105 to 0.052

Dependent variable: CTFI_log
Model: CTFI_log ~ Cell cluster + (1 | Donor)
Number of observations: 776 cells
Number of donors: 14
Estimation method: Maximum likelihood

Figure 5 (TCR sequences and repertoire analysis)

Figure 5 investigates whether the TCR repertoire of GTS-specific CD8+ T cells differs between clinical severity groups. To address the reviewer on statistical analyses, we additionally performed hierarchical clustering on paired VJ gene usage (TRA_V-TRA_J and TRB_V-TRB_J) derived from single-cell TCR data. TCR_VJ clusters were defined based on separation of major branches in the hierarchical dendrogram, reflecting discrete groupings of related paired VJ gene usage patterns.

In the acute phase, several VJ clusters were severity-specific, whereas one VJ cluster showed partial overlap across severity groups, suggesting shared repertoire usage. To statistically validate these observations, we quantified the number of cells within each zone across severity outcomes and performed Fisher-Freeman-Halton's exact test using Monte Carlo simulation (20,000 replicates). Fisher-Freeman-Halton's exact test was used to assess the overall association between VJ cluster distribution and severity. The analysis demonstrated a significant association between VJ cluster distribution and severity in the acute phase ($p = 4.99 \times 10^{-5}$), indicating differential VJ gene usage across disease outcomes (Rebuttal Figure 9).

To determine which TCR cluster contributed to this association, we examined standardized residuals from the chi-square test (Rebuttal Figure 10). Standardized residuals were used to identify enrichment or depletion patterns across individual VJ cluster-severity combinations, whereas chi-square test was used to evaluate the overall association between VJ cluster distribution and disease severity. This analysis showed that VJ cluster 2 was enriched in DF, VJ cluster 3 was enriched in AD, and VJ cluster 4 was enriched in DHF, while VJ cluster 1 showed weaker differentiation, particularly in TRB_VJ, suggesting partial sharing of TRB repertoire features between DF and DHF. Effect-size analysis using Cramer's V demonstrated strong non-random associations between VJ zone and severity (TRA_VJ = 0.95; TRB_VJ = 0.72).

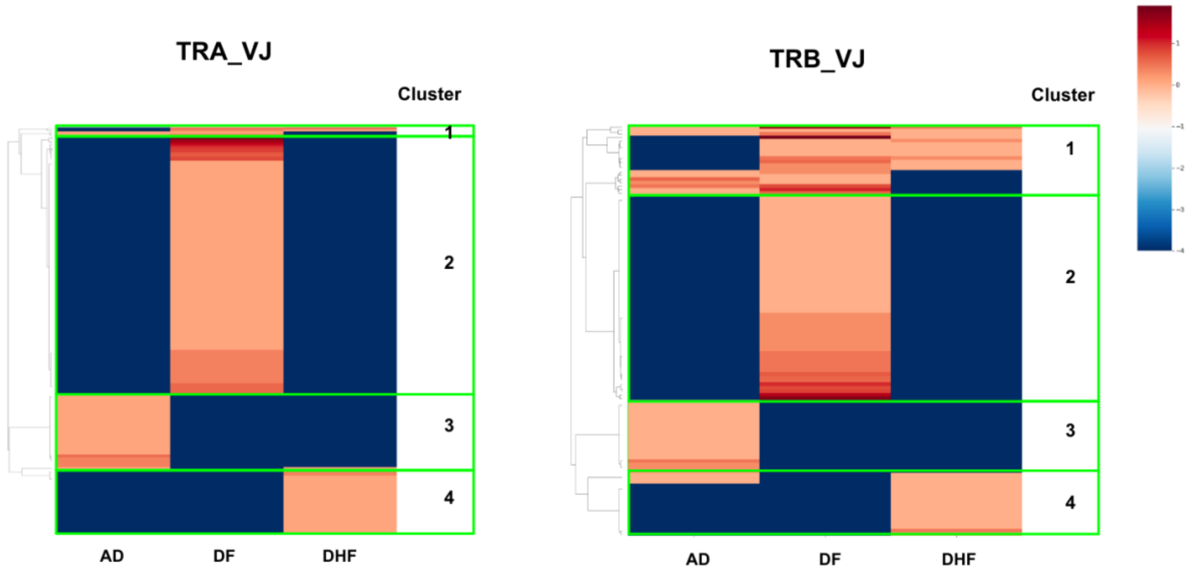

$p = 4.99 \times 10^{-6}$ $p = 4.99 \times 10^{-6}$

Rebuttal Figure 9. Hierarchical clustering heatmaps of paired VJ gene usage (TRA_VJ and TRB_VJ) in GTS-specific CD8+ T cells across disease severity groups (AD, DF, DHF) during the acute phase. Distinct TCR clusters (1-4), defined by separation of major dendrogram branches, exhibit significantly different VJ usage patterns associated with clinical severity ($p = 4.99 \times 10^{-5}$).

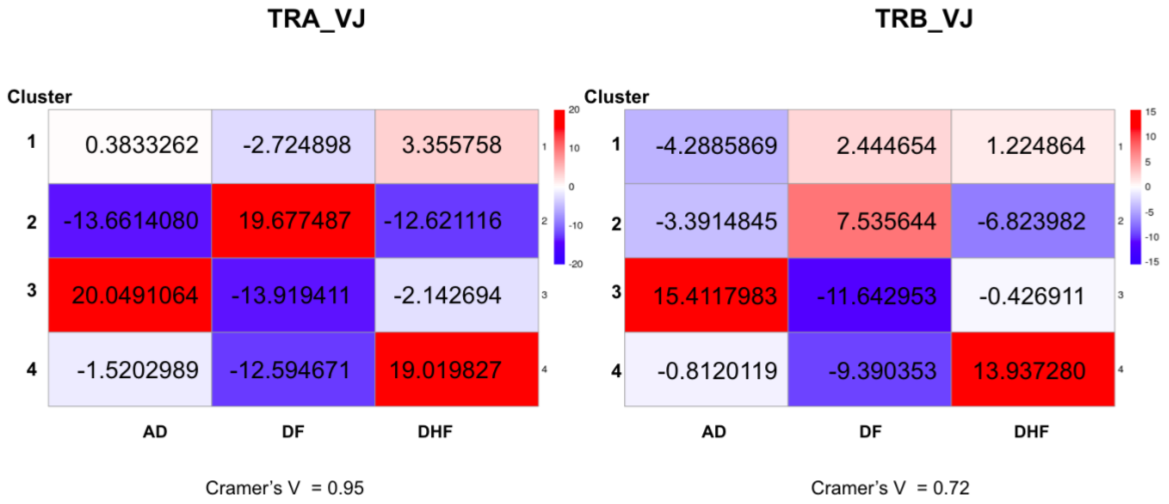

Rebuttal Figure 10. Standardized residual heatmaps showing enrichment (red) or depletion (blue) of TCR clusters across severity groups (AD, DF, DHF) for TRA_VJ and TRB_VJ usage. Cluster 2 was enriched in DF, Cluster 3 in AD, and Cluster 4 in DHF, while Cluster 1 showed weaker differentiation. Strong associations were observed between TCR cluster distribution and severity (Cramér's V: TRA_VJ = 0.95; TRB_VJ = 0.72).

In the convalescent phase, a similar overall structure was observed, with both shared and severity-specific VJ clusters. Fisher-Freeman-Halton's exact test again showed significant differences in zone distribution across severity groups ($p < 0.05$), indicating repertoire bias in the convalescent phase. Standardized residual analysis showed that, for TRA_VJ, VJ cluster 1 was enriched in DF and VJ cluster 2 in DHF, whereas VJ cluster 3 displayed weaker residual values consistent with shared usage. For TRB_VJ, VJ cluster 1 was enriched in DHF, VJ cluster 2 in DF, and VJ cluster 3 showed weak residuals, suggesting overlap between severity groups. Cramer's V values (TRA_VJ = 0.81; TRB_VJ = 0.57) confirmed continued non-random associations between VJ usage and severity during convalescence

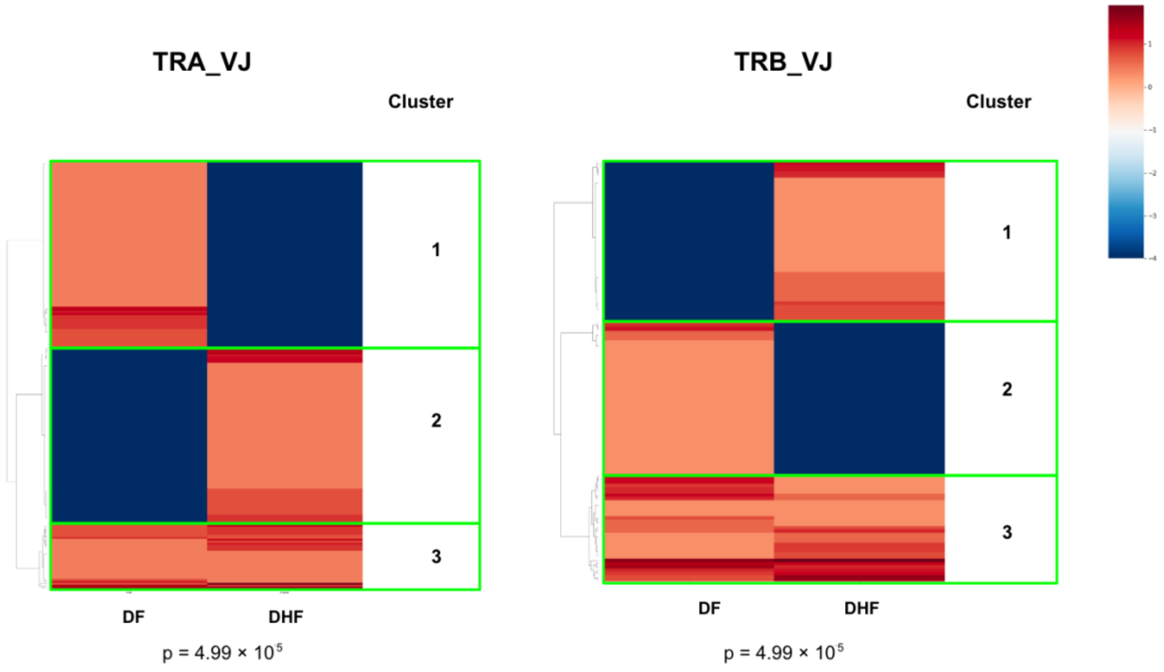

Rebuttal Figure 11. Hierarchical clustering heatmaps of paired VJ gene usage (TRA_VJ and TRB_VJ) in GTS-specific CD8+ T cells across disease severity groups (DF, DHF) during the acute phase. Distinct TCR clusters (1–3), defined by separation of major dendrogram branches, exhibit significantly different VJ usage patterns associated with clinical severity ($p = 4.99 \times 10^{-5}$).

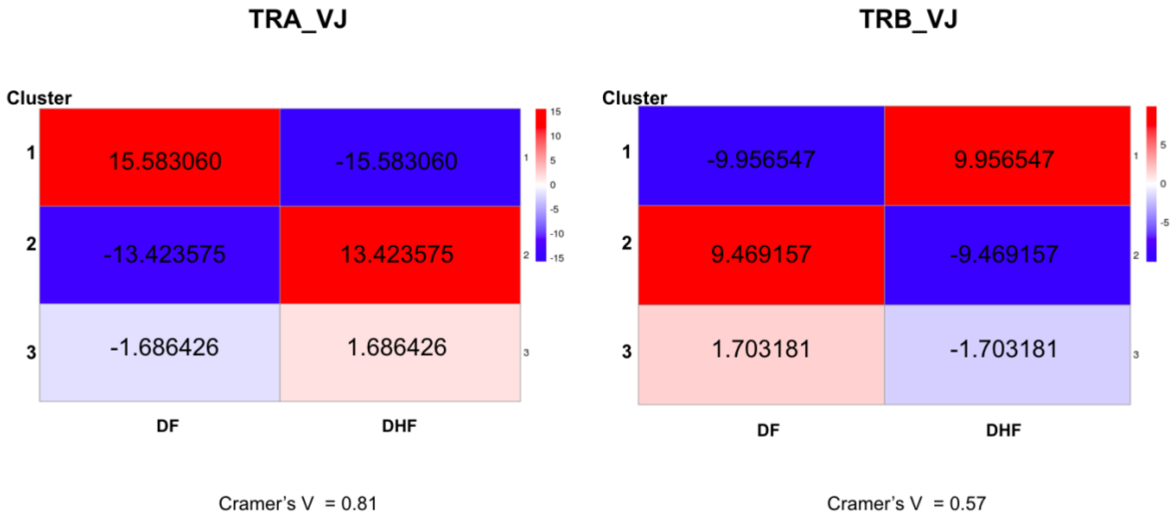

Rebuttal Figure 12. Standardized residual heatmaps showing enrichment (red) or depletion (blue) of TCR clusters across severity groups (DF, DHF) for TRA_VJ and TRB_VJ usage. For TRA_VJ, Cluster 1 was enriched in DF, Cluster 2 in DHF, while Cluster 3 showed weaker differentiation. For TRB_VJ, Cluster 1 was enriched in DHF, Cluster 2 in DF, while Cluster 3 showed weaker differentiation. Strong associations were observed between TCR cluster distribution and severity (Cramér's V: TRA_VJ = 0.81; TRB_VJ = 0.57).

This result has now been incorporated into the Results section and Supplementary Figure 8 in the revised manuscript

Added Text (Result):
Line 332: Notably, VJ cluster distribution was significantly associated with disease severity (Supplementary Figure 8)

To further evaluate global TCR repertoire relationships across disease severity with respect to individual donors at donor level, we performed principal component analysis (PCA) based on VDJ gene usage from both TCRα and TCRβ chains, including samples from acute and convalescent phases. The PCA revealed that AD samples clustered separately from symptomatic dengue (SD) groups (DF and DHF), indicating distinct repertoire composition. In contrast, DF and DHF samples are largely localized near each other, suggesting substantial overlap in VDJ gene usage patterns. Although one DHF outlier was observed, most symptomatic samples showed similar repertoire positioning in PCA space (Rebuttal Figure. 13). Consistent with the PCA distribution, hierarchical clustering based on PC1 and PC2 coordinates identified three major clusters of donors (Rebuttal Figure 14). The distribution of disease severity across these clusters was significantly associated with clinical outcome (Fisher-Freeman-Halton exact test, p = 0.0083), supporting distinct TCR repertoire organization between AD and symptomatic dengue donors.

Taken together, these results support the conclusion that the TCR repertoire in asymptomatic infection is distinct, whereas symptomatic dengue cases (DF and DHF) share more similar global VDJ usage profiles across both TCRα and TCRβ chains.

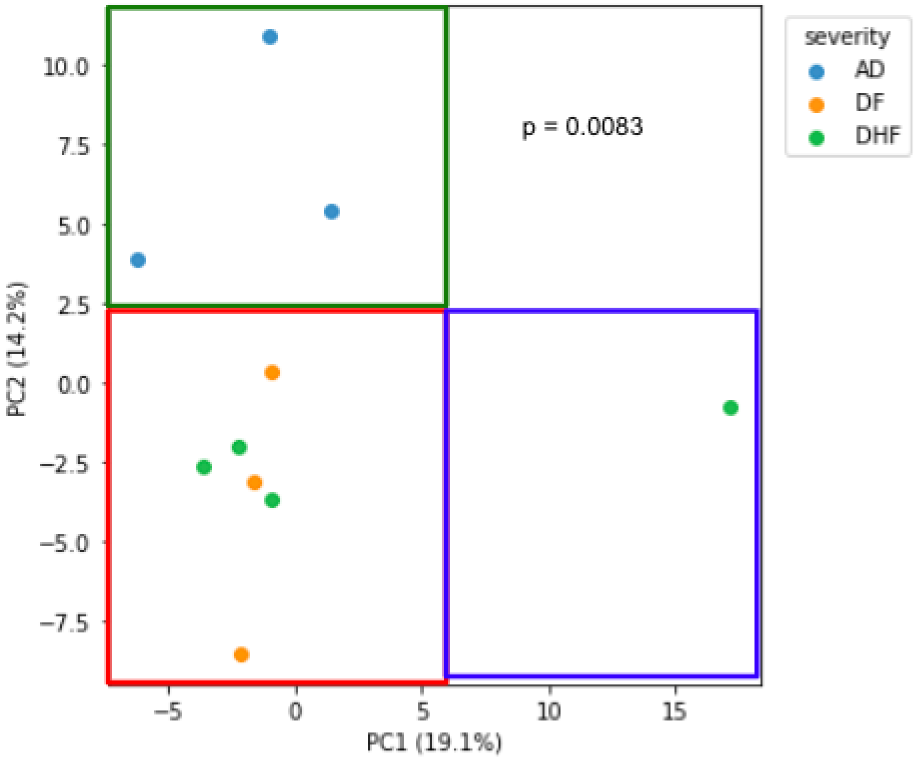

Rebuttal Figure 13. Principal component analysis (PCA) of donor-level TCR VDJ gene usage (TRA and TRB combined) from the integrated dataset combining 10x and SS2 across disease severity groups (AD, DF, DHF). AD samples cluster separately from symptomatic dengue (DF and DHF), which largely overlap in PCA space. Three clusters were defined based on the PCA distribution, and the association between cluster membership and disease severity was evaluated using the Fisher–Freeman–Halton exact test, showing a significant relationship ($p = 0.0083$).

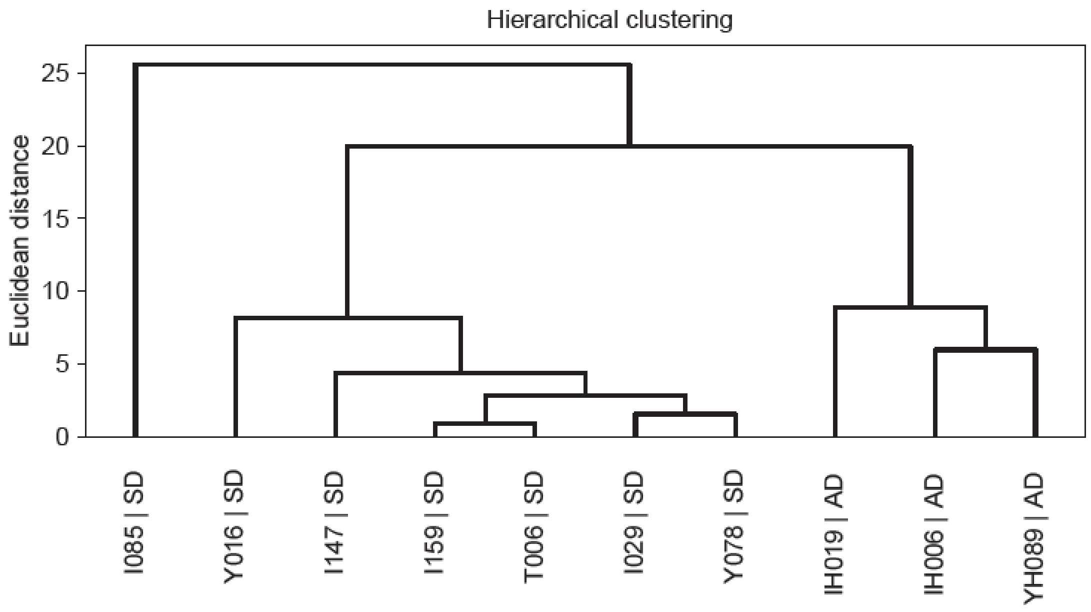

Rebuttal Figure 14. Hierarchical clustering dendrogram of donor-level samples based on Euclidean distances calculated from PC1 and PC2 coordinates derived from PCA of global TCRα and TCRβ V(D)J gene usage. Each leaf represents an individual donor labeled by donor ID and disease group. Clustering reveals separation of AD donors from symptomatic dengue (SD; DF and DHF) samples, consistent with the PCA distribution.

The results have now been incorporated into the Results section and Supplementary Figure 10 in the revised manuscript.

Added Text (Results):
Line 339: …, consistent with principal component analysis (PCA) of global TCR repertoire composition, where resulting clusters showed significant association with AD and SD labels (Supplementary Fig.10)

Finally, as suggested by the reviewer, we have revised the Methods section to describe additional statistical tests used in the analyses.

Added Text (Method):
Line 741: The composite tetramer fluorescence index across CD8+ T-cell clusters was evaluated using a linear mixed-effects regression model with the cell cluster as a fixed effect and donor as a random intercept, implemented in the statsmodels package (Supplementary Methods). Associations between TCR VJ cluster

distribution and disease severity were assessed using the Fisher-Freeman-Halton exact test with Monte Carlo simulation (scipy.stats), with effect sizes estimated using Cramer's V (Supplementary Methods). Global differences in TCR repertoire composition were evaluated using PCA of donor-level V(D)J gene usage, and the association between cluster membership and disease severity was evaluated using the Fisher–Freeman–Halton exact test.

3. Claims regarding TCR avidity and CX3CR1 enrichment continue to depend on indirect measures, such as tetramer MFI proxies, without any direct protein-level or functional validation.

The term "TCR avidity" can be defined differently across studies. In this work, we use tetramer mean fluorescence intensity (MFI) as a relative proxy for TCR–pMHC binding strength under defined staining conditions. This approach is supported by prior studies in which tetramer MFI has been used to reflect the effective strength of TCR–pMHC engagement at the cell surface[1-3]

In our experiments, tetramer staining was performed under saturating conditions, ensuring that differences in signal reflect relative binding properties rather than reagent limitation. We therefore use tetramer MFI as a comparative measure across cells, while recognizing that it does not directly measure functional avidity or signaling capacity. To address this, we already revised the manuscript in the previous response to more precisely define our use of tetramer MFI-based measurements

Line 293: Using pHLA tetramer fluorescence intensity as a proxy for TCR avidity, consistent with previous studies[27-29]

In addition, we have added detailed tetramer staining conditions in the Methods section to improve clarity.

Added Text (Method):
Line 629: Tetramer staining was performed at saturating concentrations (1 µg monomer each) to ensure maximal occupancy of available TCR binding sites.

4. Likewise, the functional significance of the observed phenotypes is not completely demonstrated, and although the limitations of ELISPOT are acknowledged, they remain unaddressed.

We appreciate this important point and agree that functional validation using assays such as ELISPOT or intracellular cytokine staining would further strengthen the study.

However, due to limited sample availability, particularly for the rare AD cases, and the very low frequency of dengue-specific CD8+ T cells in peripheral blood, the number of cells within these antigen-specific subsets is extremely limited. This makes direct functional assessment of these populations technically challenging and was not feasible within the scope of this study.

As clarified in the revised manuscript, we have more explicitly acknowledged this limitation and have framed the observed phenotypes as associations inferred from transcriptional and

cross-sectional associations, rather than directly demonstrated functional outcomes. Future studies incorporating functional assays will be important to validate these findings.

5. Overall, while the manuscript is interesting, it does not provide strong enough evidence to fully support its conclusions in its current form.

As detailed in our previous response, we have revised the manuscript to ensure that all conclusions are supported by appropriate statistical analyses and are aligned with the scope of the data. Specifically, we have strengthened the statistical framework by incorporating additional donor-level analyses, applying appropriate tests, and clearly distinguishing between exploratory observations and statistically supported findings. We have also moderated the interpretation throughout the manuscript to avoid overextension of the conclusions.

In this regard, we would appreciate it if specific analyses or conclusions requiring improvement could be identified, so that we can address the concerns directly, as a broad assessment makes it difficult for us to determine which aspects require further revision, particularly given that we have already sought to demonstrate comprehensively that our key conclusions are supported by appropriate statistical analyses in the previous revision.

Reviewer #3 (Remarks to the Author):
I thank the reviewers for their careful replies to the comments and concerns of the reviewers. However, concerns remain:

1. Sample size: the number of individuals included is limited. The identification of AD is challenging, however, more hospitalized patients could be included to increase robustness of the findings

We acknowledge that the cohort size, both in the number of donors and tetramer-positive cells, is limited. This reflects inherent biological and technical constraints, including the low frequency of antigen-specific CD8+ T cells in peripheral blood and the requirement for HLA-matched donors, as stated in the Discussion. To reduce potential confounding, donors were carefully matched for key variables, including age, sex, and viremia level.

Despite the limited sample size, severity-associated differences, including dengue-specific CD8+ T-cell phenotypes and TCR repertoire features, were evaluated using statistical analyses that account for donor-level variation. Several key comparisons reached statistical significance, indicating effect sizes sufficiently large to be detectable within this cohort. Importantly, comparisons that did not reach statistical significance are explicitly reported as a trend.

To further strengthen robustness, we integrated our data with matched droplet-based PBMC single-cell transcriptomic and TCR datasets, enabling recovery of additional shared clonotypes and inference of putative dengue-specific CD8+ T-cell populations based on TCR similarity. Key findings were also supported by independent flow cytometry validation.

We agree that inclusion of additional hospitalized patients could further improve robustness. However, expanding only the symptomatic cohort without corresponding asymptomatic dengue (AD) cases would introduce imbalance and potential confounding. We have therefore acknowledged this limitation and highlighted the need for larger, more comprehensive cohorts in future studies.

2. Amount of cells analyzed. This information is new and is now provided by the authors (Table S3). For more than half of the individuals, less than 100 cells were analyzed. This raises substantial concerns on the robustness of the findings.

We acknowledge that, for a subset of individuals, the number of tetramer-sorted CD8+ T cells analyzed was limited. Identification of dengue-specific CD8+ T cells via tetramer staining, particularly during the acute phase, is inherently challenging, as these cells are present at low frequencies in peripheral blood, especially when restricted to defined epitopes.

To ensure appropriate interpretation, statistical analyses were performed with careful consideration of study design, including donor-level aggregation to avoid cell-level pseudo-replication, the use of appropriate non-parametric tests, and correction for multiple comparisons. Additional analyses suggested by the reviewer were also incorporated. Despite these constraints, several key observations are supported by statistically significant differences and consistent effect directions across donors, indicating robust effect sizes.

Importantly, these findings are largely consistent across multiple analytical layers, including the recovery of antigen-specific CD8+ T cells from independent 10x PBMC single-cell datasets and orthogonal validation by flow cytometry. Together, these complementary approaches strengthen confidence in the observed patterns and support the conclusion that the findings are biologically meaningful despite the limited number of cells.

3. Concerns on the serotype-specificity of the tetramers used. The authors did not validate their tetramers in convalescent primary cases, as requested by me and another reviewer, so the extent of cross-reactivity remains unknown.

As noted in our previous response, inclusion of convalescent samples from RT-qPCR-confirmed primary infections was rarely feasible in our endemic setting, where truly seronegative primary cases are rare. While prior studies using similar serotype-variant peptides and pHLA tetramers have assessed epitope specificity in such settings, we recognize that the extent of cross-reactivity for the specific tetramers used in our study cannot be fully defined without direct validation.

In light of this, we have revised our interpretation and terminology to avoid overstatement. Importantly, we do not interpret tetramer binding as evidence of true serotype specificity. Instead, we describe our findings in terms of assay-defined peptide–pHLA binding behavior under controlled experimental conditions of tested tetramer staining.

Accordingly, we have removed terminology and context implying definitive serotype specificity.

Original Text (Result; previous revision):
Line 173: Reactivity of total DENV serotype-specific CD8+ T cells exhibited variable patterns of reactivity across disease severity and infection phases
To investigate how serotype-specific CD8+ T cell reactivity is related to disease severity and progression, we sorted dengue-serotype specific CD8+ T cells based on both serologically inferred dominant previous and current serotype-specific epitopes in order to determine their differential avidity, transcriptomic profiles, and TCR sequences in relation to disease severity and disease course. Single-positive staining indicated specificity for either current or inferred previous serotype, while double-positive staining identified cross-reactive cells (Fig. 1b). During the acute phase, no associations between serotype-specific reactivity patterns of total DENV-specific CD8+ T cells and disease severity were observed, with variability across individual samples (Fig. 1c-d left). Notably, one DF case exhibited a dominant cross-reactive population (Fig. 1d). During convalescence in SD samples, we found that DENV-specific CD8+ T cells, both serotype-specific and cross-reactive CD8+ T cells, were generally more abundant than that during acute phase (Fig. 1c-d right). Our findings reveal dynamic changes in total DENV-specific CD8+ T cell reactivity over the course of infection, but no association between the abundance of total DENV-specific CD8+ T cell reactivity and disease severity was observed.

Revised Text (Result; current revised manuscript):
Line 171: CD8+ T cell binding to NS3 peptides from different DENV serotypes across infection phases and disease severity
To investigate patterns of CD8+ T cell binding to NS3 peptides from different DENV serotypes in relation to disease severity and progression, we sorted CD8+ T cells based on binding to HLA tetramers loaded with NS3 peptides corresponding to the current serotype,

as defined by qPCR, and the serologically inferred dominant previously infecting DENV serotype. This enabled us to assess their differential avidity, transcriptomic profiles, and TCR sequences in relation to disease severity and disease course. Single-positive staining indicated CD8+ T cells binding tetramers loaded with NS3 peptides from one DENV serotype, whereas double-positive staining identified cells binding tetramers loaded with NS3 peptides derived from both tested DENV serotypes (Fig. 1b). During the acute phase, no associations between patterns of CD8+ T cell binding to NS3 peptides derived from different DENV serotypes and disease severity were observed, with variability across individual samples (Fig. 1c-d left). Notably, one DF case exhibited a dominant double-positive population (Fig. 1d). During convalescence in SD samples, the overall abundance of DENV NS3-specific CD8+ T cells, including both single-positive and double-positive populations was generally higher than in the acute phase (Fig. 1c-d right). Our findings reveal dynamic changes in total DENV NS3-specific CD8+ T cell binding to NS3 peptides from the current and the serologically inferred dominant previously infecting DENV serotype over the course of infection, with no significant association between the abundance of cells binding these peptides and disease severity.

Original Text (Result; previous revision):
Line 273: Serotype-specific epitope reactivity in DENV-specific CD8+ T cell subsets strongly associated with disease severity
As no significant association of serotype-specific epitope reactivity of total DENV-specific CD8+ T and disease severity outcomes was observed (Fig. 1d), we investigated whether the defined DENV-specific CD8+ T cell subsets are related to their serotype specificity and disease severity. Notably, during the acute phase, the Int CD8 subset in AD cases showed a trend toward reactivity to the currently infecting serotype, although this was not statistically significant (Fig. 3e left). In DHF, the CX3CR1+ CD8 subset was biased toward recognizing serologically inferred dominant previously infecting serotypes,...

Revised Text (Result; current revised manuscript):
Line 275: CD8+ T cell subsets show distinct patterns of binding to NS3 peptides derived from different DENV serotypes across disease severity
As no significant association of total DENV NS3-specific CD8+ T binding to NS3 epitopes from different DENV serotype and disease severity outcomes was observed (Fig. 1d), we investigated whether the defined DENV NS3-specific CD8+ T cell subsets differed in their binding to NS3 peptides from different DENV serotypes in relation to disease severity. Notably, during the acute phase, the Int CD8 subset in AD cases showed a trend toward binding more to NS3 epitopes derived from the currently infecting serotype, although this was not statistically significant (Fig. 3e left). In DHF, the CX3CR1+ CD8 subset was biased toward binding to NS3 epitopes derived from serologically inferred dominant previously infecting DENV serotypes,…

Original Text (Result; previous revision):
Line 285: …exhibited reactivity to both the current serotype and cross-reactive epitopes (Fig. 3e right). Taken together, these findings suggest that serotype reactivity, cytotoxicity magnitude, and potential localization of the DENV-specific CD8+ T cells may contribute to disease severity.

Revised Text (Result; current revised manuscript):
Line 286: …included cells with single-positive binding to the NS3 peptide from the current

infecting serotype, as well as double-positive cells binding both NS3 peptides tested (Fig. 3e right). Taken together, these findings suggest that DENV NS3-specific CD8+ T cell subsets show different patterns of binding to NS3 peptides from different DENV serotypes across disease severity.

Original Text (Discussion; previous revision):
Line 452: This approach enabled high-resolution mapping of DENV serotype-specific CD8+ T cell subsets across donors

Revised Text (Discussion; current revised manuscript):
Line 455: This approach enabled high-resolution mapping of CD8+ T cell subsets bound to different DENV serotype NS3 epitopes across donors,

Original Text (Discussion; previous revision):
Line 458: …with a trend of preferential reactivity to the current infecting serotype,...

Revised Text (Discussion; current revised manuscript):
Line 461: …with a trend of preferential binding to peptides derived from the current infecting serotype,..

Original Text (Discussion; previous revision):
Line 501: …In our study, we found that during the acute phase, DENV-specific CX3CR1+ CD8+ T cells, enriched in DHF, tend to be reactive to inferred previously encountered serotypes, while Int CD8+ T cells, enriched in AD, had a trend to be reactive to the currently infecting ones.

Revised Text (Discussion; current revised manuscript):
Line 505: …In our study, we found that during the acute phase, DENV NS3-specific CX3CR1+ CD8+ T cells, enriched in DHF, tend to bind to peptides derived from serologically inferred dominant previously infecting DENV serotype, while Int CD8+ T cells, enriched in AD, had a trend to bind to peptides derived from the currently infecting ones.

This revision reflects a deliberate shift from inferring biological serotype specificity to describing directly observed binding patterns. These definitions do not imply absolute specificity and do not exclude the possibility of cross-reactivity.

Additionally, we have also included the peptide epitope sequences used for tetramer generation in the manuscript (Supplementary Table 3), corresponding to NS3-derived epitopes restricted by HLA-A*11 and HLA-A*24, and these are now clearly described in the Methods section.

Table S3. Peptide sequences used for pHLA tetramer generation

HLA restriction	Epitope variant	Serotype	Amino acid sequence	Protein	Position
HLA-A*11	GTS1.1	DENV1	GTSGSPIVNR	NS3	133–142
HLA-A*11	GTS2.1	DENV2	GTSGSPIIDK	NS3	133–142
HLA-A*11	GTS3.1	DENV3/4	GTSGSPIINR	NS3	133–142
HLA-A*24	NYA1.1	DENV1	QYSDRRWCF	NS3	556–564

HLA-A*24	NYA2.1	DENV2	NYADRRWCF	NS3	556–564
HLA-A*24	NYA3.1	DENV3	KYTDRKWCF	NS3	556–564
HLA-A*24	NYA4.1	DENV4	SYKDREWCF	NS3	556–564

Added Text (Method):
Line 624: The amino acid sequences of all peptide variants used for tetramer generation are provided in Supplementary Table 3

4. The limitation on knowledge on previous infecting serotype. Point 3 and 4 together make that the claims “a trend of biased reactivity to the infecting serotype” and the designation “serotype specific” and “cross reactive” CD8+ T cells highly uncertain and not supported by sufficient controls and is highly speculative. All analysis regarding this topic should be omitted from the manuscript.

As noted above, we have revised the terminology to avoid implying definitive serotype specificity and instead describe results based on the experimental readout of tetramer binding under controlled conditions. Specifically, terms such as “serotype-specific” and “cross-reactive” have been replaced with binding-based descriptions that directly reflect the assay.

We agree that definitive conclusions regarding serotype specificity cannot be made given the limitations in determining prior infecting serotype and the potential for TCR cross-reactivity. Accordingly, we have removed statements suggesting “biased reactivity to the infecting serotype” and have reframed these analyses to emphasize relative tetramer-binding patterns rather than true serotype-specific immune responses.

While we acknowledge that these limitations preclude strong claims of serotype specificity, we respectfully retain these analyses in a revised and more cautious form, as they provide insight into antigen-specific CD8+ T cell behavior under defined experimental conditions. We have ensured that their interpretation is appropriately constrained and no longer overstated.

References

1. Mallone, R. et al. Functional avidity directs T-cell fate in autoreactive CD4+ T cells. Blood 106, 2798-2805, doi:10.1182/blood-2004-12-4848 (2005).
2. Laugel, B. et al. Different T cell receptor affinity thresholds and CD8 coreceptor dependence govern cytotoxic T lymphocyte activation and tetramer binding properties. J Biol Chem 282, 23799-23810, doi:10.1074/jbc.M700976200 (2007).
3. Zhong, S. et al. T-cell receptor affinity and avidity defines antitumor response and autoimmunity in T-cell immunotherapy. Proc Natl Acad Sci U S A 110, 6973-6978, doi:10.1073/pnas.1221609110 (2013).
